# SPIRIT 2025 explanation and elaboration: updated guideline for protocols of randomised trials

**DOI:** 10.1136/bmj-2024-081660

**Published:** 2025-04-28

**Authors:** Asbjørn Hróbjartsson, Isabelle Boutron, Sally Hopewell, David Moher, Kenneth F Schulz, Gary S Collins, Ruth Tunn, Rakesh Aggarwal, Michael Berkwits, Jesse A Berlin, Nita Bhandari, Nancy J Butcher, Marion K Campbell, Runcie C W Chidebe, Diana R Elbourne, Andrew J Farmer, Dean A Fergusson, Robert M Golub, Steven N Goodman, Tammy C Hoffmann, John P A Ioannidis, Brennan C Kahan, Rachel L Knowles, Sarah E Lamb, Steff Lewis, Elizabeth Loder, Martin Offringa, Philippe Ravaud, Dawn P Richards, Frank W Rockhold, David L Schriger, Nandi L Siegfried, Sophie Staniszewska, Rod S Taylor, Lehana Thabane, David J Torgerson, Sunita Vohra, Ian R White, An-Wen Chan

**Affiliations:** 1 Centre for Evidence-Based Medicine Odense and Cochrane Denmark, Department of Clinical Research, University of Southern Denmark, Odense, Denmark; 2 Open Patient data Explorative Network, Odense University Hospital, Odense, Denmark; 3 Université Paris Cité and Université Sorbonne Paris Nord, Inserm, INRAE, Centre for Research in Epidemiology and Statistics (CRESS), Paris, France; 4 Centre d’Epidémiologie Clinique, Hôpital Hôtel Dieu, AP-HP, Paris, France; 5 Oxford Clinical Trials Research Unit, Centre for Statistics in Medicine, University of Oxford, Oxford, UK; 6 Centre for Journalology, Clinical Epidemiology Programme, Ottawa Hospital Research Institute, Ottawa, ON, Canada; 7 Department of Obstetrics and Gynaecology, School of Medicine, University of North Carolina at Chapel Hill, Chapel Hill, NC, USA; 8 UK EQUATOR Centre, Centre for Statistics in Medicine, University of Oxford, Oxford, UK; 9 Jawaharlal Institute of Postgraduate Medical Education and Research, Puducherry, India; 10 *JAMA* and the JAMA Network, Chicago, IL, USA; 11 Department of Biostatistics and Epidemiology, School of Public Health, Centre for Pharmacoepidemiology and Treatment Science, Rutgers University, New Brunswick, NJ, USA; 12 *JAMA Network Open*, Chicago, IL, USA; 13 Centre for Health Research and Development, Society for Applied Studies, New Delhi, India; 14 Child Health Evaluation Services, The Hospital for Sick Children Research Institute, Toronto, ON, Canada; 15 Department of Psychiatry, University of Toronto, Toronto, ON, Canada; 16 Aberdeen Centre for Evaluation, University of Aberdeen, Aberdeen, UK; 17 Project PINK BLUE-Health and Psychological Trust Centre, Utako, Abuja, Nigeria; 18 Department of Sociology and Gerontology and Scripps Gerontology Centre, Miami University, OH, USA; 19 Department of Medical Statistics, London School of Hygiene and Tropical Medicine, London, UK; 20 Nuffield Department of Primary Care Health Sciences, University of Oxford, Oxford, UK; 21 Ottawa Hospital Research Institute, Ottawa, ON, Canada; 22 Department of Medicine, Northwestern University Feinberg School of Medicine, Chicago, IL, USA; 23 Department of Epidemiology and Population Health, Stanford University, Palo Alto, CA, USA; 24 Institute for Evidence-Based Healthcare, Faculty of Health Sciences and Medicine, Bond University, Robina, QLD, Australia; 25 Departments of Medicine, of Epidemiology and Population Health, of Biomedical Data Science, and of Statistics, and Meta-Research Innovation Centre at Stanford (METRICS), Stanford University, Stanford, CA, USA; 26 MRC Clinical Trials Unit at University College London, London, UK; 27 University College London, UCL Great Ormond Street Institute of Child Health, London, UK; 28 Faculty of Health and Life Sciences, University of Exeter, Exeter, UK; 29 Edinburgh Clinical Trials Unit, Usher Institute-University of Edinburgh, Edinburgh BioQuarter, Edinburgh, UK; 30 *The BMJ*, BMA House, London, UK; 31 Brigham and Women’s Hospital, Harvard Medical School, Boston, MA, USA; 32 Université Paris Cité, Inserm, INRAE, Centre de Recherche Epidémiologie et Statistiques, Université Paris Cité, Paris, France; 33 Clinical Trials Ontario, MaRS Centre, Toronto, ON, Canada; 34 Duke Clinical Research Institute, Duke University Medical Centre, Durham, NC, USA; 35 Department of Emergency Medicine, University of California, Los Angeles, CA, USA; 36 Mental Health, Alcohol, Substance Use, and Tobacco Research Unit, South African Medical Research Council, Cape Town, South Africa; 37 Warwick Applied Health, Warwick Medical School, University of Warwick, Coventry, UK; 38 MRC/CSO Social and Public Health Sciences Unit and Robertson Centre for Biostatistics, Institute of Health and Wellbeing, University of Glasgow, Glasgow, UK; 39 Department of Health Research Methods Evidence and Impact, McMaster University, Hamilton, ON, Canada; 40 St Joseph’s Healthcare Hamilton, Hamilton, ON, Canada; 41 York Trials Unit, Department of Health Sciences, University of York, York, UK; 42 Faculty of Medicine and Dentistry, University of Alberta, Edmonton, AB, Canada; 43 Department of Medicine, Women’s College Research Institute, University of Toronto, Toronto, ON, Canada

## Abstract

High quality protocols facilitate proper planning, conduct, reporting, and external review of randomised trials, yet their completeness varies and key elements are often not considered. To strengthen good reporting of trial protocols, the SPIRIT (Standard Protocol Items: Recommendations for Interventional Trials) 2013 statement has been updated to incorporate new evidence and emerging perspectives. This SPIRIT 2025 explanation and elaboration document provides users with exemplars of reporting in contemporary trial protocols, contextual elaboration, more detailed guidance on reporting, references to key empirical studies, an expanded checklist, and a link to a website for further information. The document is intended to be used in conjunction with the SPIRIT 2025 statement and serves as a resource for researchers planning a trial and for others interested in trial protocols.

“Readers should not have to infer what was probably done; they should be told explicitly.”

Douglas G Altman^1^

Every randomised trial requires an adequate protocol—a text that details the trial’s rationale, aim, methods, organisation, and ethical considerations.^2^ Trial investigators use protocols to document plans for the conduct of a study at all stages from the recruitment of participants to the dissemination of results. Funding agencies, research ethics committees or institutional review boards, regulatory agencies, medical journals, systematic reviewers, and others rely on protocols to appraise the conduct and reporting of clinical trials.

The usefulness of trial results to patients, healthcare providers, and policy makers depends on how well the trial was planned. Adequate reporting of a trial protocol conveys that the core methodological and logistical challenges of running the trial have been considered, and possibly dealt with, before the trial commences. In this respect, reporting guidelines for protocols might facilitate improvement in plans for a trial in addition to an improvement in reporting of those plans.

Concerns about inadequate reporting of key content in trial protocols^3^ ^4^ ^5^ provided the impetus for the development of the original SPIRIT (Standard Protocol Items: Recommendations for Interventional Trials) statement published in 2013.^6^ Modelled on the CONSORT (Consolidated Standards of Reporting Trials) statement for reporting the results of randomised trials,^7^ the international SPIRIT initiative aimed to improve the reporting of trial protocols by producing evidence based recommendations for a minimum set of items to be considered in protocols. The SPIRIT 2013 statement has been translated into seven languages and has been endorsed, among others, by national funders, research organisations, more than 150 medical journals, and the World Association of Medical Editors.^8^

Although the reporting of protocols for randomised trials has improved somewhat since 2013 it remains far from adequate.^9^ ^10^ ^11^ ^12^ Also, as the content and context of randomised trials evolve, both SPIRIT and CONSORT statements require periodic updating to reflect new evidence, methodological advances, and feedback from users. Thus, an international group developed and published the updated SPIRIT 2025 statement^13^—consisting of a 34 item checklist of the minimum recommended protocol items (table 1); a diagram illustrating the schedule of enrolment, interventions, and assessments (fig 1). SPIRIT 2025 reflects the collaboration and input of multiple contributors, including trial investigators, healthcare professionals, methodologists, statisticians, patient and public contributors, journal editors, and research funders. SPIRIT 2025 was developed in parallel with the updated CONSORT 2025 to harmonise recommendations and terminology.^7^ ^14^

**Table 1:** SPIRIT 2025 checklist of items to address in a randomised trial protocol

Section/topic	No	SPIRIT 2025 checklist item description
**Administrative information**		
Title and structured summary	1a	Title stating the trial design, population, and interventions, with identification as a protocol
	1b	Structured summary of trial design and methods, including items from the World Health Organization Trial Registration Data Set
Protocol version	2	Version date and identifier
Roles and responsibilities	3a	Names, affiliations, and roles of protocol contributors
	3b	Name and contact information for the trial sponsor
	3c	Role of trial sponsor and funders in design, conduct, analysis, and reporting of trial; including any authority over these activities
	3d	Composition, roles, and responsibilities of the coordinating site, steering committee, endpoint adjudication committee, data management team, and other individuals or groups overseeing the trial, if applicable
**Open science**		
Trial registration	4	Name of trial registry, identifying number (with URL), and date of registration. If not yet registered, name of intended registry
Protocol and statistical analysis plan	5	Where the trial protocol and statistical analysis plan can be accessed
Data sharing	6	Where and how the individual de-identified participant data (including data dictionary), statistical code, and any other materials will be accessible
Funding and conflicts of interest	7a	Sources of funding and other support (eg, supply of drugs)
	7b	Financial and other conflicts of interest for principal investigators and steering committee members
Dissemination policy	8	Plans to communicate trial results to participants, healthcare professionals, the public, and other relevant groups (eg, reporting in trial registry, plain language summary, publication)
**Introduction**		
Background and rationale	9a	Scientific background and rationale, including summary of relevant studies (published and unpublished) examining benefits and harms for each intervention
	9b	Explanation for choice of comparator
Objectives	10	Specific objectives related to benefits and harms
**Methods: Patient and public involvement, trial design**		
Patient and public involvement	11	Details of, or plans for, patient or public involvement in the design, conduct, and reporting of the trial
Trial design	12	Description of trial design including type of trial (eg, parallel group, crossover), allocation ratio, and framework (eg, superiority, equivalence, non-inferiority, exploratory)
**Methods: Participants, interventions, and outcomes**		
Trial setting	13	Settings (eg, community, hospital) and locations (eg, countries, sites) where the trial will be conducted
Eligibility criteria	14a	Eligibility criteria for participants
	14b	If applicable, eligibility criteria for sites and for individuals who will deliver the interventions (eg, surgeons, physiotherapists)
Intervention and comparator	15a	Intervention and comparator with sufficient details to allow replication including how, when, and by whom they will be administered. If relevant, where additional materials describing the intervention and comparator (eg, intervention manual) can be accessed
	15b	Criteria for discontinuing or modifying allocated intervention/comparator for a trial participant (eg, drug dose change in response to harms, participant request, or improving/worsening disease)
	15c	Strategies to improve adherence to intervention/comparator protocols, if applicable, and any procedures for monitoring adherence (eg, drug tablet return, sessions attended)
	15d	Concomitant care that is permitted or prohibited during the trial
Outcomes	16	Primary and secondary outcomes, including the specific measurement variable (eg, systolic blood pressure), analysis metric (eg, change from baseline, final value, time to event), method of aggregation (eg, median, proportion), and time point for each outcome
Harms	17	How harms are defined and will be assessed (eg, systematically, non-systematically)
Participant timeline	18	Time schedule of enrolment, interventions (including any run-ins and washouts), assessments, and visits for participants. A schematic diagram is highly recommended (see fig 1)
Sample size	19	How sample size was determined, including all assumptions supporting the sample size calculation
Recruitment	20	Strategies for achieving adequate participant enrolment to reach target sample size
**Methods: Assignment of interventions**		
Randomisation:		
Sequence generation	21a	Who will generate the random allocation sequence and the method used
	21b	Type of randomisation (simple or restricted) and details of any factors for stratification. To reduce predictability of a random sequence, other details of any planned restriction (eg, blocking) should be provided in a separate document that is unavailable to those who enrol participants or assign interventions
Allocation concealment mechanism	22	Mechanism used to implement the random allocation sequence (eg, central computer/telephone; sequentially numbered, opaque, sealed containers), describing any steps to conceal the sequence until interventions are assigned
Implementation	23	Whether the personnel who will enrol and those who will assign participants to the interventions will have access to the random allocation sequence
Blinding	24a	Who will be blinded after assignment to interventions (eg, participants, care providers, outcome assessors, data analysts)
	24b	If blinded, how blinding will be achieved and description of the similarity of interventions
	24c	If blinded, circumstances under which unblinding is permissible, and procedure for revealing a participant’s allocated intervention during the trial
**Methods: Data collection, management, and analysis**		
Data collection methods	25a	Plans for assessment and collection of trial data, including any related processes to promote data quality (eg, duplicate measurements, training of assessors) and a description of trial instruments (eg, questionnaires, laboratory tests) along with their reliability and validity, if known. Reference to where data collection forms can be accessed, if not in the protocol
	25b	Plans to promote participant retention and complete follow-up, including list of any outcome data to be collected for participants who discontinue or deviate from intervention protocols
Data management	26	Plans for data entry, coding, security, and storage, including any related processes to promote data quality (eg, double data entry; range checks for data values). Reference to where details of data management procedures can be accessed, if not in the protocol
Statistical methods	27a	Statistical methods used to compare groups for primary and secondary outcomes, including harms
	27b	Definition of who will be included in each analysis (eg, all randomised participants), and in which group
	27c	How missing data will be handled in the analysis
	27d	Methods for any additional analyses (eg, subgroup and sensitivity analyses)
**Methods: Monitoring**		
Data monitoring committee	28a	Composition of data monitoring committee (DMC); summary of its role and reporting structure; statement of whether it is independent from the sponsor and funder; conflicts of interest and reference to where further details about its charter can be found, if not in the protocol. Alternatively, an explanation of why a DMC is not needed
	28b	Explanation of any interim analyses and stopping guidelines, including who will have access to these interim results and make the final decision to terminate the trial
Trial monitoring	29	Frequency and procedures for monitoring trial conduct. If there is no monitoring, give explanation
**Ethics**		
Research ethics approval	30	Plans for seeking research ethics committee/institutional review board approval
Protocol amendments	31	Plans for communicating important protocol modifications to relevant parties
Consent or assent	32a	Who will obtain informed consent or assent from potential trial participants or authorised proxies, and how
	32b	Additional consent provisions for collection and use of participant data and biological specimens in ancillary studies, if applicable
Confidentiality	33	How personal information about potential and enrolled participants will be collected, shared, and maintained in order to protect confidentiality before, during, and after the trial
Ancillary and post-trial care	34	Provisions, if any, for ancillary and post-trial care, and for compensation to those who suffer harm from trial participation

**Fig 1:**
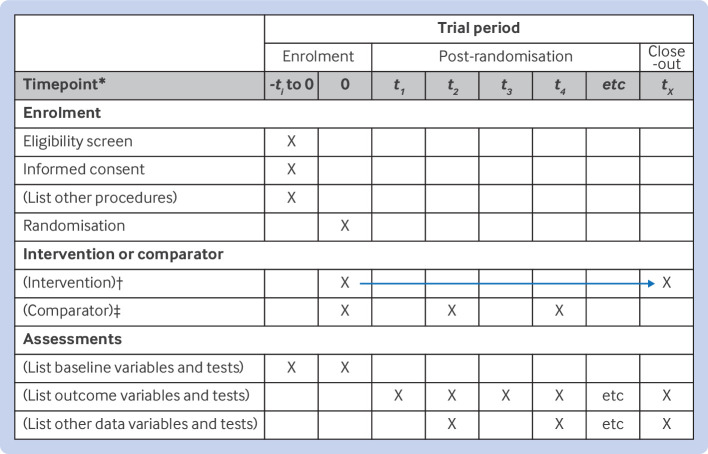
Schedule of enrolment, interventions, and assessments for SPIRIT 2025 statement. Recommended content can be displayed using various schematic formats. *List target time points and acceptable time windows (eg, 30±3 days) in this row. †Arrow indicates continuous delivery of intervention (eg, drug). ‡Example illustrates delivery of intervention or comparator at discrete time points (eg, psychotherapy). The brackets indicate placeholder text that should be replaced with the actual text for a specific trial. SPIRIT=Standard Protocol Items: Recommendations for Interventional Trials

SPIRIT 2025 is intended as a guide for those preparing a protocol for a randomised trial. The recommendations are not intended to prescribe how a trial should be designed or conducted.^15^ Rather, we call for a transparent and adequate description of what was planned. SPIRIT 2025 concerns the minimum content for trial protocols; additional considerations might be important to describe in protocols for trials of specific designs, such as crossover trials, or in protocols intended for submission to specific groups (eg, funders, regulatory agencies).

This SPIRIT 2025 explanation and elaboration document describes the rationale and scientific background for each reporting item included in the SPIRIT 2025 statement (table 1) and presents examples of good protocol reporting. We aimed to provide the basis for a fuller understanding of each checklist item and more detailed guidance for reporting. The document is primarily intended to be used in conjunction with the SPIRIT 2025 statement,^13^ but it may also serve as an overview of the core problems and methodological studies relevant to the reporting of trial protocols.

## Methods

The methods used to update the SPIRIT and CONSORT statements followed the EQUATOR (Enhancing the Quality and Transparency Of Health Research) Network guidance for developers of health research guidelines.^16^ The methods have been described in detail elsewhere.^17^ ^18^ ^19^ ^20^

Briefly, we constructed a project database of potentially relevant publications,^20^ conducted a scoping review of published comments suggesting changes to SPIRIT 2013 and CONSORT 2010,^18^ identified relevant SPIRIT or CONSORT extensions and related guidelines (eg,^21^ ^22^ ^23^ ^24^) and collected evidence and recommendations from other sources (eg, personal files and personal communications). We then generated a list of potential modifications or additions to SPIRIT and CONSORT, which we presented to interested parties in an online Delphi survey involving 317 international participants. The Delphi survey results were then discussed at a two day online expert consensus meeting attended by 30 international experts before we drafted the updated SPIRIT 2025 and CONSORT 2025 checklists and finalised them based on further feedback from people who attended the meeting. The SPIRIT 2025 and CONSORT 2025 statements are published elsewhere,^13^ ^14^ where full details of the changes to the checklists are described.

We modelled our approach for developing the SPIRIT 2025 explanation and elaboration document on the procedures for the SPIRIT 2013 explanation and elaboration document.^25^ We present each checklist item with at least one exemplar from a protocol, followed by an explanation of the rationale and main issues to address, and a list of selected references to relevant empirical and theoretical evidence.

To identify examples for each checklist item, we obtained protocols from public websites, journal publications, and appendices to published trials. Exemplars were selected to reflect how key elements could be appropriately described in a trial protocol. Some examples illustrate a specific component of a checklist item, whereas others encompass all key recommendations for an item. Examples are quoted verbatim from the trial protocol. Proper names of trial staff have been abbreviated (except for item 3a). Finally, any reference numbers cited in the original quoted text are denoted by [reference] to distinguish them from references cited in this explanation and elaboration paper.

Members of the core writing group (IB, A-WC, GSC, SH, AH, DM, KFS, RT) developed a draft for individual items. The draft was revised iteratively after comments from all authors.

For each checklist item, we provided references to core empirical studies supporting its inclusion, which we identified through a continuously updated project database.^20^ Studies were cited if they provided empirical data to support or challenge a given protocol concept. When citing empirical evidence, we aimed to reference a systematic review when available. Some items had little or no identified empirical evidence (eg, item 1a, Title) but their inclusion in the checklist was supported by a strong pragmatic or ethical rationale.

To facilitate implementation of SPIRIT 2025, we also developed an expanded version of the SPIRIT 2025 checklist, with bullet points.^26^ The expanded checklist includes an abridged version of elements presented in this SPIRIT 2025 explanation and elaboration document, with examples and references removed (see supplementary appendix). We also developed a joint SPIRIT-CONSORT website^8^ (see supplementary appendix) to provide more information about both statements, including additional resources and training materials aimed at researchers, research trainees, journal editors, and peer reviewers. The website also includes resources aimed at patients and the public that explain the importance of clear and transparent reporting of randomised trials and their importance in the delivery of evidence based healthcare.

**Summary points**

The SPIRIT 2025 statement comprises a checklist of 34 essential items for reporting protocols of randomised trials
The explanation and elaboration document provides detailed guidance, describes the rationale and scientific background for each SPIRIT 2025 checklist item, and provides published examples of good reporting
The document serves as a resource for researchers planning a trial, and for others who are interested in the content and reporting of trial protocols

## Explanation and elaboration of reporting items

### Item 1a: Title stating the trial design, population, and interventions, with identification as a protocol

#### Example

“Multicentre, national, investigator-initiated, randomised, parallel-group, register-based superiority trial to compare extended ECG monitoring versus standard ECG monitoring in elderly patients with ischaemic stroke or transient ischaemic attack and the effect on stroke, death and intracerebral bleeding: the AF SPICE protocol.”^27^

#### Explanation

The title provides an important means of trial identification. A succinct description that conveys the topic (study population, intervention, comparator) and trial design (eg, parallel group randomised trial) will facilitate retrieval from electronic database or internet searches and rapid judgment of relevance.^28^ It can also be helpful to include the trial framework (eg, superiority, non-inferiority), objective, or primary outcome, and, if relevant, the study phase (eg, phase 2) and acronym.

#### Summary of key elements to address

Descriptive title stating:

- trial design (eg, parallel group randomised trial);
- conceptual framework (eg, superiority, non-inferiority);
- trial phase (if applicable);
- population;
- intervention or comparator;
- objective or primary outcome; and
- protocol.

### Item 1b: Structured summary of trial design and methods, including items from the World Health Organization Trial Registration Data Set

#### Example

See table 2 adapted from Australian and New Zealand Clinical Trials Registry.^29^

**Table 2:** WHO Trial Registration Data Set items. Adapted from Australian and New Zealand Clinical Trials Registry^29^

	
Primary registry and trial identifying number	ANZCTR ACTRN12624000133538
Date of registration in primary registry	14-02-2024
Secondary identifying numbers	NA
Universal trial number (UTN)	U1111-1299-5067
Source(s) of monetary or material Support	Rockfield Trust
Primary sponsor	University of Auckland
Secondary sponsor(s)	NA
Contact for public queries	T E [name] Phone:+64 . . . E-Mail: . . .
Contact for scientific queries	Professor W C [name] Phone:+64 . . . Email: . . .
Public title	Gut bugs for *C difficile* infection
Scientific title	Encapsulated faecal microbiome transfer versus oral vancomycin treatment for sustained cure of recurrent or refractory *Clostridioides difficile* infection
Countries of recruitment	New Zealand
Health condition(s)	Recurrent or refractory *Clostridioides difficile* infection
Intervention(s)	Arm 1: FMT alone Participants in this group will be randomly assigned to receive . . . Arm 2: Vancomycin alone Participants in this group will be randomly assigned to receive . . . Arm 3: Vancomycin-FMT Participants in this group will be randomly assigned to receive . . .
Key inclusion and exclusion criteria	Inclusion criteria: 1. Aged 16 to 90 years; AND 2. Ability to swallow all treatment capsules; AND 3. Having: a. First recurrent *Clostridioides difficile* infection; OR b. Refractory *Clostridioides difficile* infection. Exclusion criteria: As a real world trial examining FMT effectiveness, there will be minimal exclusion criteria. However, patients meeting the three below will be excluded: . . .
Study type	Purpose: Treatment Allocation: Randomised controlled trial Masking: Blinded (masking used) Assignment: Parallel Type of endpoint: Efficacy Phase 3
Date of first enrolment	01/03/2024
Sample size	84
Recruitment status	Not yet recruiting
Primary outcome(s)	Rate of sustained *Clostridioides difficile* infection cure 12 weeks after treatment commencement with FMT alone compared to vancomycin hydrochloride alone [Clinical evaluation]. *Clostridioides difficile* infection sustained cure is defined as . . .
Key secondary outcomes	*Clostridioides difficile* infection sustained cure rate at 12 weeks after treatment initiation . . .
Ethics review	Status: Approved Approval date: 22/11/2023 Contact: Northern B Health and Disability Ethics Committee
Data sharing statement	What data will be shared: Clinical deidentified data and associated meta-data documentation and deidentified post-filtered metagenomic sequencing data Data will be available to approved applicants after the publication of the main findings within three years of the trial finishing Clinical data access requests to be sent to the Liggins Institute’s Clinical Data Research Hub Data Access Committee (email: dataservices@auckland.ac.nz). Metagenomic sequencing data will be shared on NCBI’s Sequence Read Archive (SRA)

ANZCTR=Australian New Zealand Clinical Trials Registry; FMT=faecal microbiome transfer; NA=not applicable; NCBI=National Center for Biotechnology Information; WHO=World Health Organization.

#### Explanation

A structured summary provides an accessible overview of the planned trial for users of the protocol. The WHO Trial Registration Data Set should be included in the protocol to serve as a brief structured summary of the trial. This dataset defines the minimum information to be included in a trial registry for a trial to be considered fully registered (item 4).^30^ These standards are supported by the International Committee of Medical Journal Editors (ICMJE), other journal editors, and legislation in many countries.^31^ ^32^ ^33^

Reporting of the dataset in the protocol can facilitate the prospective transfer of accurate information to a trial registry (ie, before inclusion of the first trial participant). This can also signal the need for updates to the registry record when associated protocol sections are amended—thereby promoting consistency of information between the protocol and registry.

#### Summary of key elements to address

Relevant items from the WHO Trial Registration Data Set^30^:

- primary registry and trial identifying number;
- secondary identifying numbers;
- source(s) of monetary or material support;
- primary sponsor;
- contact for public queries;
- contact for scientific queries;
- public title;
- scientific title;
- countries of recruitment;
- health condition(s) or problem(s) studied;
- intervention(s);
- key inclusion and exclusion criteria;
- study type;
- date of first enrolment (planned);
- sample size;
- primary outcome(s);
- key secondary outcome(s);
- ethics review; and
- individual trial participant data sharing statement.

### Item 2: Version date and identifier

#### Example

“Version/Date: Version 6.0/Amendment 5 (27 March 2022)

Replaces: Version 5.0/Amendment 4 (15 September 2020) . . .

Revision

Secondary endpoint duration of mechanical ventilation was changed to prevalence of prolonged (>7 days) postoperative mechanical ventilation.

Rationale

This change was made because “prolonged mechanical ventilation” is captured as a specific complication variable in the . . . database. Historically sites capture complication variables very reliably and accurately.”^34^

#### Explanation

Amendments to the trial protocol are common.^35^ ^36^ Sequentially labelling and dating each protocol version helps to mitigate potential confusion over which document is the most recent. Explicitly listing the changes relative to the previous version, with the rationale for the change, is also important (item 31). Transparent tracking of versions and amendments facilitates trial conduct, review, and oversight.

#### Summary of key elements to address

- Version date
- Version identifier (eg, version 2.0)
- List of changes relative to the previous protocol version, with reasons.

### Item 3a: Names, affiliations, and roles of protocol contributors

#### Example

“Jennifer Hemingway-Foday^1^, Alan Tita^2^, Elwyn Chomba^3^, Musaku Mwenechanya^3^, Trecious Mweemba^3^, Tracy Nolen^1^, Adrien Lokangaka^4,5^, . . . Richard Derman^19^, William A Petri^20^, Marion Koso-Thomas^21^, Elizabeth McClure^1^, Waldemar A Carlo^2^

^1^RTI International, Research Triangle Park, North Carolina, USA

^2^The University of Alabama at Birmingham School of Medicine, Birmingham, Alabama, USA

^3^University of Zambia, University Teaching Hospital, Lusaka, Zambia

^4^University of Kinshasa, Kinshasa, Congo (the Democratic Republic of the)

^5^Kinshasa School of Public Health, Kinshasa, Congo (the Democratic Republic of the) . . .

^19^Office of Global Affairs, Thomas Jefferson University, Philadelphia, Pennsylvania, USA

^20^University of Virginia, Charlottesville, Virginia, USA

^21^Eunice Kennedy Shriver National Institute of Child Health and Human Development, Bethesda, Maryland, USA

Contributors

AT and WAC conceived of the study and developed the protocol, with input from EMC, TN, JH-F, MK-T and PLH. TN developed the statistical analyses plan. JH-F wrote the first draft of the manuscript and subsequent revisions, with critical feedback from WAC, AT, EMC, MK-T and TN. EC, MMwenechanya, TM, AL, ATK, GL, PLH, AP, PKD, KK, SSG, AK, MMetgud, SS, SST, FE, PN, AS, LF, MMazariegos, SMB, RH, MSS, RG, MB, CB, EAL, OAE, NFK, RD and WAP contributed to the refinement and finalisation of the study protocol and trial implementation. All authors contributed to the preparation of this manuscript and have reviewed and approved the final version.”^37^

#### Explanation

Listing the protocol authors, their affiliations, and their role in the protocol development process provides due recognition, accountability, and transparency. Naming of authors can also help to identify conflicts of interest (item 7b) and reduce ghost and gift authorship.^38^ ^39^ ^40^ ^41^

The naming of authors and a description of their roles are standard for protocols published in journals such as *Trials* and *BMJ Open*, but they are less common for unpublished protocols. Reviews of two samples of 108 and 292 unpublished trial protocols from 2016 found that 11% and 17%, respectively, reported the names of protocol contributors or authors.^9^ ^10^

Individuals who contribute substantially to the development and drafting of a protocol should be named as authors or listed as contributors. For example, if statisticians or professional medical writers participated in drafting the protocol, then they should be listed. Non-human artificial intelligence (AI) tools (ie, large language models, machine learning) do not qualify for authorship, but their use to create content or assist in writing the protocol should be clearly stated—for example, under acknowledgments or in a methods section.^42^ ^43^

#### Summary of key elements to address

For each protocol contributor:

- name;
- affiliation; and
- description of contributions, including use of AI technologies if applicable.

### Item 3b: Name and contact information for the trial sponsor

#### Example

“Sponsor:

University of Oxford

Research Governance, Ethics and Assurance, Joint Research Office, 

Boundary Brook House

Churchill Drive, Headington,

Oxford, OX3 7GB, United Kingdom

rgea.sponsor@admin.ox.ac.uk

EudraCT Number: 2021-004267-27.”^44^

#### Explanation

The sponsor can be defined as the individual, company, institution, or organisation assuming overall responsibility for the initiation and management of the trial but is not necessarily the funder.^45^ ^46^ In general, the company is the sponsor in industry initiated trials, whereas the institution of the primary investigator is often the sponsor for investigator initiated trials. For some investigator initiated trials, the principal investigator can be considered to be a sponsor-investigator, assuming the roles of both sponsor and investigator.^46^

Identification of the trial sponsor provides transparency and accountability and can also help to identify conflicts of interest (item 7b). Reviews of two samples of 108 and 292 trial protocols from 2016 found that 57% and 78%, respectively, reported the name and contact details of the sponsor.^9^ ^10^

The protocol should identify the name, contact information, and, if applicable, regulatory agency identifying number of the sponsor.

#### Summary of key elements to address

For the trial sponsor (eg, individual, company, institution, or organisation):

- name;
- contact information; and
- regulatory agency identifying number, if applicable.

### Item 3c: Role of trial sponsor and funders in design, conduct, analysis, and reporting of trial; including any authority over these activities

#### Example

“The study is funded by the National Health and Medical Research Council (NHMRC) of Australia by a project grant (1140756). RNB is supported by an NHMRC Research Fellowship (1037220). The study is sponsored by The University of Queensland. The University of Queensland and the funder have no influence on study design, collection, management, analysis and interpretation of data, writing of the report and the decision to submit for publication.”^47^

#### Explanation

Trials are typically funded by drug or device companies (ie, industry funded) or by scientific, private, or governmental foundations or organisations (ie, non-industry funded). The various types of funders differ in their overall agenda, reasons for funding a trial, and propensity to influence the trial. A funder will typically provide direct monitory support for a trial, but funding may also be provided indirectly, such as through free trial drugs, equipment, or services (eg, statistical analysis or use of medical writers) (item 7a).

The more direct the influence a trial funder (or sponsor) with a strong interest in a specific study result has on the design, conduct, analysis, and reporting of a trial, the higher is the risk that their interests interfere with the scientific purpose of the trial. Undue influence can result in methodological problems (eg, inappropriate comparator group or narrow patient eligibility criteria) and bias.

Most industry initiated trials are controlled by the company sponsoring and funding the trial; this authority is often enforced by contractual agreements signed between the company and trial investigators.^38^ ^48^ Restrictions on publication have been imposed by various groups, including industry sponsors.^38^ ^49^ ^50^ These restrictions are sometimes not fully described in the protocol but rather in separate publication agreements.^38^ ^51^

Industry funded trials with greater funder involvement tend to report more favourable results (ie, larger estimates of intervention effects) than comparable trials without such involvement. One review of eight published meta-epidemiological studies reported that trials with a high risk of influence from industry funders, such as on trial design, conduct, and analysis, reported 12% (odds ratio) more beneficial effects (95% confidence interval (CI) 3% to 19%), on average, than comparable trials without high risk of funder influence.^52^ Reviews of two samples of 108 and 292 trial protocols from 2016 found that 61% and 39%, respectively, reported the role of the sponsor and funder.^9^ ^10^

The protocol should explicitly outline the roles of the sponsor and funders in trial design, conduct, data analysis and interpretation, manuscript writing, and dissemination of results. It is also important to state whether the sponsor or funder makes the final decision on any of these aspects of the trial or has the right to review or comment on the trial manuscript. Any restrictions on publications should be disclosed in the protocol for review by research ethics committees and other relevant parties. If any mechanisms were planned to mitigate funder influence, this should be reported. Also, the authors should explicitly state if the funder will have no direct involvement in the trial.

#### Summary of key elements to address

For the sponsor and funders:

- roles and responsibilities in trial design, conduct, data analysis and interpretation, manuscript writing, and dissemination of results;
- who will make final decision on the above trial aspects;
- whether the sponsor or funder will have the right to review or comment on the trial manuscript; and
- any mechanisms used to mitigate funder influence. If the funder will have no direct involvement in the trial, then this should be explicitly stated.

### Item 3d: Composition, roles, and responsibilities of the coordinating site, steering committee, endpoint adjudication committee, data management team, and other individuals or groups overseeing the trial, if applicable

#### Example

“A steering committee is responsible for the design, integrity, and progress of the trial. The steering committee is also tasked with implementing any potential protocol modifications, including those that may be recommended by the DMC [data monitoring committee]. The steering committee comprises staff of the UMC [University Medical Centre] Groningen and lead investigators of all participating study centers. An independent DMC has been appointed to carry out the safety-related tasks described above. The DMC consists of three internist-nephrologists and an epidemiologist. All DMC members declared no competing interests. The DMC charter is available as Supplementary material. An academic contract research organization will monitor the progress of the study and the quality of study data. Additionally, a patient advisory committee consisting of several patients . . . was assembled to provide input to the steering committee from a patient perspective.

Figure 3 shows the structure of the study organisation and the relationships between its components.”^53^

#### Explanation

A randomised trial will often include various committees or groups to facilitate trial coordination and conduct—for example, a trial management group (day-to-day trial conduct) and a trial steering committee (executive decisions).^54^ ^55^

Information on the types, conduct, and membership of such committees helps to ensure that roles and responsibilities are clearly understood at the onset of the trial and facilitates communication from external parties about the trial. Information also enables readers to understand the mandate and expertise of those responsible for overseeing participant safety, trial design, database integrity, and study conduct.

The complexity of a trial, and the need for a clear division of responsibilities and a transparent organisational structure, increase with the number of included sites and participants. Of particular importance is the trial steering committee and its independence from the trial sponsor or funder. The data monitoring committee is also central and is described in more detail in item 28a on trial monitoring. Reviews of two samples of 108 and 292 trial protocols from 2016 found that 98% and 92%, respectively, reported the steering committee membership and roles.^9^ ^10^

The protocol should outline the general membership of the various committees or groups involved in the coordination and conduct of the trial; describe the roles and responsibilities of each, including their relationship to the trial sponsor or funder; and (when known) identify the chairs and members of each committee.

#### Summary of key elements to address

For each trial committee:

- roles and responsibilities;
- relationship to trial sponsor and funders;
- outline of membership (eg, clinician, biostatistician, patient); and
- names of chairs and members of each committee, when known.

Examples of committees include:

- trial steering committee (executive decisions);
- trial management group (day-to-day trial conduct);
- data monitoring committee (review of accumulating data);
- endpoint adjudication committee;
- data management team; and
- other individuals or groups overseeing the trial.

### Item 4: Name of trial registry, identifying number (with URL), and date of registration. If not yet registered, name of intended registry

#### Example

“Australian New Zealand Clinical Trials Registry (ANZCTR)

ACTRN12622000949785

Date registered: 05/07/2022

www.anzctr.org.au/Trial/Registration/TrialReview.aspx?ACTRN=12622000949785.”^56^

#### Explanation

The ethical and scientific reasons for trial registration are compelling.^57^ ^58^ ^59^ ^60^ ^61^ ^62^ Documentation of a trial’s existence and prespecified outcomes in a publicly accessible registry helps to increase transparency, decrease unnecessary duplication of research effort, facilitate identification of ongoing trials for prospective participants, and identify and deter selective reporting of study results.^63^ ^64^ A trial registration number can serve to link all of the documents related to a trial, starting with the protocol.^65^ As mandated by the ICMJE and jurisdictional legislation,^31^ ^32^ ^66^ registration of clinical trials should occur before recruitment of the first trial participant.

The protocol should state the name of the registry and the assigned registration number. If the trial is not yet registered, the intended registry should be named and registration details updated before participant enrolment. When registration in multiple registries is required (eg, to meet regulations), each identifier should be listed in the protocol and each registry. Online protocol authoring tools such as the SPIRIT Electronic Protocol Tool and Resource (SEPTRE) enable automated transfer of relevant information to trial registries (eg, ClinicalTrials.gov).^67^

Particular attention should be given to registering complete descriptions of protocol specified outcomes (item 16), as previous reviews have reported frequent discrepancies in the primary outcomes defined in registries compared with those specified in protocols of randomised trials.^64^

#### Summary of key elements to address

- Name of registry
- Trial registry identifying number
- URL to registry record
- Date of registration.

### Item 5: Where the trial protocol and statistical analysis plan can be accessed

#### Examples

“The full protocol, dataset, and statistical code are available in the Open Science [Framework] repository, DOI https://doi.org/10.17605/OSF.IO/6BA3W.”^68^

“A detailed justification and explanation of these stages is included in the statistical analysis plan (SAP; see online supplemental file 2).”^69^

#### Explanation

The protocol describes the study design and methods, including the main features of the statistical analysis (item 27) and sample size calculation (item 19). The statistical analysis plan is an associated document that provides full technical details of the planned analyses for benefits and harms and their execution.^70^ ^71^ Comprehensive guidance is available on the content of statistical analysis plans.^71^ Typically, the statistical analysis plan is a separate document that should be read in conjunction with the protocol, but for some trials with simple analyses, it can be included as part of the protocol.^72^

Changes to the protocol and statistical analysis plan may happen after the trial begins, often for legitimate reasons—for example, in response to new evidence or unanticipated challenges.^73^ These amendments should be transparently recorded in each new document version (item 31).

Given the central role of protocols and statistical analysis plans in enhancing the transparency, reproducibility, and interpretation of trial results, there is a strong ethical and scientific rationale for these key documents to be publicly available.^74^ ^75^ ^76^ ^77^ ^78^ High quality protocols and statistical analysis plans contain relevant information on study design and conduct that is often not reported in journal publications or trial registries.^79^ ^80^ Having access to these documents may help with trial appraisal, interpretation, and replication, as well as identification of selective outcome reporting.^81^ ^82^

The protocol should indicate whether and how it will be made publicly available, along with the statistical analysis plan (if separate). Avenues for providing access to protocols and statistical analysis plans include standalone publications in journals, such as *Trials* and *BMJ Open*,^3^ ^4^ or posting the documents as an online appendix to the publication of trial results.

Most trial registries allow the full protocol and statistical analysis plan to be included along with the trial’s registration record. Other options include online repositories and preprint servers. Many journals routinely publish a statement about sharing of protocols, statistical codes, and datasets for all their published research articles.^83^

#### Summary of key elements to address

- Where the protocol will be accessible (eg, publication, repository such as Open Science Framework, trial registry)
- Where the full statistical analysis plan will be accessible.

### Item 6: Where and how the individual deidentified participant data (including data dictionary), statistical code, and any other materials will be accessible

#### Examples

“Within 3 months of the end of the final year of funding a description of the study dataset, including a code book, a SAS file of the code used for creating the final study sample, the final study variables and plan for conducting the outcomes analyses outlined in the study protocol will be made available. The investigators will create a complete, cleaned, de-identified copy of the final data set . . . A section in the MGH Health Decision Sciences Center website will be created to hold study materials and it will include information for investigators interested in accessing these materials and replicating the findings. The PI will share a de-identified data set with outside investigators according to the policies in the approved IRB [institutional review board] protocol. Investigators may be required to provide evidence of IRB approval (or exemption) and/or complete a data sharing agreement.”^84^

“The principal investigator will oversee the intra-study data sharing process. All investigators at individual study sites will be given access to the cleaned data sets. Data sets will be housed on the file transfer protocol site created for the study, and all data sets will be password protected. Investigators will have direct access to their own site’s data sets and will have access to other sites data by request. To ensure confidentiality, any data dispersed to investigators will be blinded of any identifying participant information.”^85^

#### Explanation

Data sharing typically involves making accessible the deidentified individual participant data generated from the trial and the data dictionary detailing the data elements, along with the analytical code used to carry out the planned data analyses.

A trial’s data can be stored in a data archive and shared in a variety of ways, such as through an institutional repository (for example, belonging to the university associated with the trial’s coordinating centre) or a public facing repository such as Vivli, GitHub, or the YODA Project. Data can be shared at the time of the main trial publication, or only after a specified embargo period, such as after one year, enabling the trial team to complete planned secondary projects.

Data sharing increases transparency, facilitates reproducibility, reduces research waste, and provides data for additional exploration. Some trial groups have worked collaboratively to conduct individual patient data meta-analysis.^86^ Data sharing is also associated with an increased number of citations.^87^ ^88^

Concerns about data sharing involve a perceived risk of reidentification of individuals, especially in trials enrolling participants with rare diseases. Also, inappropriate use of the data by other researchers or groups with a particular agenda can be a barrier to sharing.^89^

Consent for data sharing can be obtained from trial participants upon enrolment, whereas approval for data sharing is typically obtained from research ethics committees and data access committees.^90^ In a growing number of jurisdictions (eg, Canada, US, UK), some funders now require a commitment on the part of researchers to share their data (eg, US National Institutes of Health, UK National Institute for Health and Care Research, Gates Foundation). Similarly, some journals require data sharing statements as part of the article submission process (eg, *The BMJ, PLOS Medicine*).^91^ ^92^

However, data sharing does not happen for most randomised trials.^93^ ^94^ ^95^ ^96^ ^97^ For example, among 224 randomised trials of interventions for covid-19, 68 (30%) reported in a trial registry the intention to share data, but only 50 (22%) actually shared individual patient data upon request or in a repository.^96^

Access to the trial data is also sometimes restricted for trial investigators, and not only for individuals outside the trial. The World Medical Association supports the principle that trial investigators retain the right to access the data.^98^ Results from randomised trials might be more reproducible and trustworthy when the complete final dataset can be accessed by multiple investigators who contributed the data.

For some multicentre trials, only the trial steering committee has access to the full trial dataset. The rationale is to ensure that the overall results are not revealed by an individual study site before the main publication. Many of these trials will allow site investigators to access the full dataset if the steering committee approves a formal request describing their plans.

For some trials, especially those sponsored by industry, access to the final trial dataset is restricted to the sponsor, which can introduce concerns about potential conflicts of interest (item 3c).^99^ Reviews of two samples of 108 and 292 trial protocols from 2016 found that 23% and 32%, respectively, reported plans for who would have access to the full dataset.^9^ ^10^

The protocol should identify those individuals involved in the trial who will have access to the final dataset, and it should explicitly describe any restrictions to access as well as provide the rationale for any such restrictions. The protocol’s data sharing plans should state whether (and if so, how) the deidentified data, data dictionary, and statistical code will be shared with individuals not otherwise involved in the trial. Details on developing a data sharing plan can be found elsewhere.^100^

#### Summary of key elements to address

- What data and materials will be shared; for example:
  - deidentified individual participant data and data dictionary, analytical code used to process the data; and
  - materials associated with the intervention, such as a handbook or video for non-drug interventions.
- How the data and materials will be shared with trial investigators and external parties, including:
  - application process to access the data (if applicable);
  - data transfer process, such as through a repository or direct transfer to user; and
  - any plans to obtain consent from participants.
- If no sharing is planned, this should be clearly stated.

### Item 7a: Sources of funding and other support (eg, supply of drugs)

#### Examples

“The study is funded by the National Health and Medical Research Council (NHMRC) of Australia by a project grant (1140756). RNB is supported by an NHMRC Research Fellowship (1037220).”^47^

“Funding Information

The authors disclosed receipt of the following financial support for the research, authorship, and/or publication of this article: This work was supported by the Pfizer Global Investigator-Initiated Research Grant (grant no. GA6120A8) and Blue Cross Blue Shield of Michigan Foundation, Investigator-Initiated Grant (grant no. 002607.II).”^101^

#### Explanation

A randomised trial requires considerable funding, typically from drug or device companies (ie, industry funded) or from scientific or private foundations, or governmental or non-governmental organisations (ie, non-industry funded).^102^ One study estimated the median cost for each phase 3 industry funded trial to be $21.4m (£16.9m; €20.5m),^103^ with substantial variation. The mean cost of clinical trials funded by the UK National Institute for Health and Care Research was lower but still sizeable at about £1.3m, and also with considerable variation.^104^ The various types of funders differ in their overall agenda, reasons for funding a trial, and propensity to influence the trial.

Funding of a trial typically involves direct monetary support, but financial support might also be provided indirectly in the form of free trial drugs, equipment, or services such as statistical analysis or use of medical writers.^105^

Although both industry funded and non-industry funded trials are susceptible to bias, industry funding is associated with conclusions that favour the intervention. A systematic review of 75 methodological studies, comparing industry funded with non-industry funded studies (mostly randomised trials), reported that industry studies more often had favourable conclusions (risk ratio 1.34 (95% CI 1.19 to 1.51) compared with non‐industry studies.^106^

Industry funded trials might also report more favourable results (ie, estimates of intervention effects) than comparable trials that are funded by other sources, although the evidence is less certain. One review of eight published meta-epidemiological studies reported that, on average, odds ratios from industry funded trials did not differ significantly from those of non-industry funded trials. However, in the subset of trials with a high risk of industry funder influence (eg, on trial design, conduct, and analysis), the difference was 12% (95% CI 3% to 19%).^52^ Undue influence on trials from non-industry funders with a strong interest in a specific trial result has been described,^107^ although studied less.

There is no clear consensus on a threshold for when funding from a source with conflicting interests becomes problematic, nor on the characteristics of a possible dose-response association. It is debatable whether industry funding within itself is less important than the degree and type of funder influence on trial design, conduct, analysis, and reporting (item 3c). Indirect financial support, such as provision of various medical services, will impact differently on various stages of a trial. For example, use of industry paid medical statisticians might have an effect on analysis, and use of industry paid medical writers might influence reporting.

Reviews of two samples of 108 and 292 trial protocols from 2016 found that 91% and 83%, respectively, described funding sources.^9^ ^10^ A systematic review of five studies compared funding sources reported in protocols or trial registries with funding sources declared in the corresponding publications of the results. It found discrepancies in a median of 45% (range 4-80%) of protocol-publication pairs (not exclusively randomised trials).^64^

Reporting the funding source and the role of the trial funder (item 3c) provides important context for readers of a protocol when ascertaining the feasibility of the trial as well as its overall methodological quality (for example, the relevance of the type of comparator intervention and eligibility criteria for patients) and risk of bias (for example, the risk of selective reporting of favourable results). At a minimum, the protocol should identify the sources of direct and indirect financial support and the specific type (eg, funds, equipment, drugs, services).

#### Summary of key elements to address

For each funding source:

- name of funder; and
- type of funding:
  o direct monetary support; and
  o indirect support (free trial drugs, equipment, or services such as statistical analysis or use of medical writers).

### Item 7b: Financial and other conflicts of interest for principal investigators and steering committee members

#### Examples

“Competing interests: None declared.”^47^

“EW has been invited to advisory boards from Recordati. WS has received a speaker’s honorarium from Mag&More GmbH and neurocare and was a member of the advisory board of Recordati. AH has received speakership fees from Lundbeck, . . . and Recordati. He was member of advisory boards of Boehringer Ingelheim, . . . and Recordati. ML has received honoraria for consultancy and speakers’ fees from AstraZeneca, . . . Takeda Pharma Vertrieb GmbH, and is founder of MiNDNET e-Health-Solutions GmbH. PF is on the advisory boards and receives speaker fees from Janssen, Lundbeck, Otsuka, Servier and RBL has received honoraria for consultancy and speakers’ fees from ANM, AstraZeneca, . . . and Sound Therapeutics; All other co-authors report no conflict of interest.”^108^

#### Explanation

Conflicts of interest (also known as competing interests) may be defined as a “a set of circumstances that creates a risk that professional judgement or actions regarding a primary interest will be unduly influenced by a secondary interest.”^109^ In the context of a clinical trial, conflicts of interest imply a risk that investigators’ personal interests or ties with companies or organisations will unduly influence the design, conduct, analysis, or reporting of a trial. The concept implies a risk of influence and is not indicative of actual wrongdoing.

Disclosure of interests enables a management plan to be developed and implemented and facilitates full ascertainment of conflicts of interest, risk of bias, and overall methodological quality—for example, the relevance of the type of comparator intervention.

Types of financial ties include salary support or grants, ownership of stock or options, honorariums (eg, for advice, authorship, or public speaking), paid consultancy or service on advisory boards and speakers’ bureaus, and the holding of patents or patents pending. Non-financial conflicts of interest include academic commitments, personal or professional relationships, and political, religious, or other affiliations with special interests or advocacy positions.

A cross sectional study of 190 randomised trials published in core clinical journals found that trials with stated authors’ conflicts of interest had more positive results than trials without. The presence of a financial tie was associated with a positive study outcome (odds ratio 3.23, 95% CI 1.7 to 6.1). This association was also present after adjustment for the study funding source (3.57, 1.7 to 7.7).^110^ Conflicts of interest are most often associated with drug and device industries, but they may exist with support from or affiliation with government agencies, charities (not-for-profit organisations), and professional and civic organisations,^107^ although these associations have been much less investigated. Reviews of two samples of 108 and 292 trial protocols from 2016 found that 65% and 62%, respectively, reported declarations of interests.^9^ ^10^

The protocol should identify conflicts of interest of the principal investigators and members of main committees—for example, the first author, the last author, and the corresponding author of the protocol, the trial statistician or epidemiologist, and the heads and members of the trial steering and data monitoring committees. The protocol should also describe procedures planned to reduce the risk that conflicts of interest could influence the trial’s design, conduct, analysis, or reporting.

#### Summary of key elements to address

- Conflicts of interests for principal trial investigators and members of key committees involved in the trial (eg, steering and data monitoring committees), including any of the following support received:
  - financial: salary support or grants, ownership of stock or options, honorariums (eg, for advice, authorship, or public speaking), paid consultancy or service on advisory boards, and holding of patents or patents pending; and
  - non-financial: academic commitments, personal or professional relationships, and political or other affiliations with special interests or advocacy positions.
- Any procedures planned to reduce the risk that conflicts of interest could influence the trial’s design, conduct, analysis, or reporting
- If no conflict of interests exist, this should be clearly stated.

### Item 8: Plans to communicate trial results to participants, healthcare professionals, the public, and other relevant groups (eg, reporting in trial registry, plain language summary, publication)

#### Examples

“The PI [principal investigator] and study team have developed a plan to promote dissemination and implementation of the study findings to consumer, clinical and payer stakeholders. The PAC [Patient Advisory Committee] will facilitate dissemination of the study and results to patient, advocate and community audiences. One key role the PAC will play is to develop and maintain relationships with local and regional organisations that may assist in disseminating the results. Presentations at local meetings (eg, grand rounds), at national meetings (eg, American Academy of Orthopaedic Surgeons) as well as publications in leading journals will be used to reach physicians more broadly. In addition, the team will convene an external advisory board made up of clinician, payer, researcher and consumer representatives to guide dissemination and implementation efforts. This group will convene for one in-person meeting and two calls over the study period. These external advisors are experts across different domains (clinical care, payers, patient advocacy and consumer groups) who can help disseminate study findings more broadly.”^84^

“We have registered the trial in ClinicalTrials.gov. After data collection is complete, and within two years of completion, we will report results in ClinicalTrials.gov, including a flow chart of study recruitment and dropout, demographic and baseline characteristics of participants, primary and secondary outcomes (adherence, CD4, viral load), statistical test results, and AE [adverse event] information if applicable. After complying with NIH reporting requirements in ClinicalTrials.gov, we will undertake multi-faceted dissemination to make results available to a range of stakeholder audiences, including: (1) meetings in Vietnam with public health officials at all levels, interested patients, and the HIV community; (2) peer-reviewed publications in HIV, social science, and policy and health systems journals; and (3) presentations at national and international conferences, including the International Association of Providers of AIDS Care (IAPAC)’s annual International Adherence Conference. In the preparation of abstracts and publications, we will follow the criteria for authorship recommended by the International Committee of Medical Journal Editors (ICMJE); we do not intend to employ professional writers. If findings indicate, we will work with partners in Vietnam to plan a larger effectiveness study.”^111^

#### Explanation

A fundamental ethical principle in clinical trials is that the potential risks incurred by study participants should be balanced by the benefit of contributing to publicly available knowledge and future patients. Journal publication remains a key means of reporting trial results. Open access publishing provides patients, the public, and other interested parties with immediate and free access to read the trial’s results, with reduced copyright barriers.

However, a high proportion of clinical trials remain unpublished.^112^ ^113^ Despite providing important information relevant to patients, trials with statistically non-significant results are more prone to non-publication,^114^ or, when published, to a long delay to publication^114^ ^115^ ^116^—often referred to as publication bias. Overall, the medical literature represents a biased subset of existing data, potentially leading to overestimation of benefits, underestimation of harms, and a detrimental impact on patient care and research.^112^ ^117^ ^118^ Although peer reviewers and journal editors can be biased in favour of positive findings,^119^ lack of publication seems to be primarily due to trial investigators or sponsors failing to submit negative or null results, rather than journals rejecting them.^114^ ^120^

Beyond journal publication, trial registries and preprint servers are important venues for dissemination.^121^ ^122^ ^123^ Meta-research studies have found that more than a quarter of unpublished trials in various countries had results available in trial registries, and some data (particularly harms) were more completely reported in registries than in journal publications.^113^ ^124^ ^125^ ^126^ ^127^ Almost all journals are supportive of preprinting trial protocols and completed studies^128^ before formal journal submission.^129^ ^130^ ^131^ Initial data suggest a few differences between trial results on preprint servers and those on final publication.^132^ ^133^ ^134^ Another publication format is Registered Reports, which involves registration, peer review, and (possibly) preacceptance of a trial before data collection.^135^

Dissemination of trial results is closely linked to authorship. Substantive contributions to the design, conduct, interpretation, and reporting of a clinical trial are recognised through the granting of authorship on the final trial report.^43^ Individuals who fulfil authorship criteria should not remain hidden (ghost authorship) and should have final authority over manuscript content. Similarly, those who do not fulfil such criteria should not be granted guest authorship.

Trials would not be possible without the voluntary contributions of participants. Communication of the trial results directly to the trial participants is widely considered an ethical responsibility supported by both researchers and participants.^136^ A plain language summary of results for the wider public also serves to increase public knowledge and inform future patients.^137^

In a sample of 326 protocols approved in 2012, 70 (21%) trials remained unpublished after 10 years.^113^ Twenty three of 147 investigator sponsored trials (16%) reported their results in a trial registry in contrast with 150 of 179 industry sponsored trials (84%). Also, publication of trial results was associated with higher quality reporting in the protocol.^113^ In two other samples of 108 and 292 protocols from 2016, plans to disseminate trial results were described in 56% and 71%, respectively.^9^ ^10^

The trial protocol should report the dissemination plan for the trial findings, including posting of trial results in a registry and publication in a journal. It is also important to describe how results will be conveyed to trial participants and broader audiences. The plan should include a process and timeframe for approving and submitting reports for dissemination, as well as authorship criteria.

#### Summary of key elements to address

- Plan to disseminate trial results to participants, healthcare professionals, the public, and other relevant groups (eg, reporting results in trial registry, preprint, plain language summary, publication in an open access journal)
- Process and timeframe for approving and submitting reports for dissemination
- Authorship guidelines.

### Item 9a: Scientific background and rationale, including summary of relevant studies (published and unpublished) examining benefits and harms for each intervention

#### Examples

“1. Background

[Introduction]: Over 300 million people undergo major surgical procedures annually.[reference] A significant proportion of patients experiences perioperative neurocognitive disorders (NCDs) after surgery including delirium and postoperative neurocognitive disorder (P-NCD) (mild and major P-NCD) . . .

[Existing knowledge]: In recent years, dexmedetomidine (DEX) has gained attention for its potential perioperative therapeutic effects. DEX is a potent and selective α2 receptor (α2R) agonist approved for procedural sedation by anaesthesiologists or as a sedative in the intensive care unit (ICU). Recent studies indicate that DEX reduces postoperative delirium after cardiac and non-cardiac surgery.[references to systematic review and individual randomised trials]

[Mechanisms]: In murine models, DEX reduces anaesthetic/surgery-induced learning and memory deficits from propofol, etomidate, benzodiazepines and halogenated inhalational anaesthetics.[references] These salutary effects on learning and memory have also been demonstrated when DEX is administered in murine models of sepsis without any surgical insult or anaesthetic exposure.[references] The mechanisms of DEX neuroprotection are proposed to be both direct and indirect. Directly, it has opioid/GABA-ergic drug-sparing effects.[reference] Indirectly, it has anti-inflammatory effects in the CNS and is protective of neuro-apoptosis.[reference] The synergistic anaesthetic sparing and anti-inflammatory effects of DEX in the CNS [central nervous system] could reduce the incidence of major P-NCD after open cardiac surgery, the surgical population with the highest observed risk . . .

[Need for a trial]: The evidence for DEX reducing major P-NCD, while biologically plausible, is limited by suboptimal outcome measurement in terms of both neurocognitive assessments and the dose of DEX highlighting the need for a large, well designed trial.

With approximately 300 000 open cardiac procedures occurring yearly in North America, the burden of P-NCD is enormous. Currently, there are no interventions to prevent persistent cognitive impairment after surgery. Thus, effective mitigation strategies for major P-NCD are urgently needed.”^138^

“[Introduction]: Traumatic brain injury (TBI) is a significant public health concern and represents the leading cause of mortality and long-term disability in young adults [reference] . . .

[Mechanism]: the cerebral autoregulation that normally compensates for variations in oxygen delivery is impaired,[reference] rendering their brain vulnerable to ischaemia and secondary injuries. In the absence of high-quality evidence, several experts have suggested maintaining higher haemoglobin (Hb) levels (>100 g/L) on the assumption that it reduces metabolic distress and improves brain tissue oxygenation [references] . . .

[Existing evidence]: The evidence to support transfusion strategies in patients with TBI remains scarce. In a systematic review of studies in neurocritical care patients, we found insufficient evidence to support the use of a specific transfusion threshold to improve morbidity and mortality.[reference] A recent randomised controlled trial showed no effect of red blood cell (RBC) transfusion on neurological outcomes in patients with moderate or severe TBI, although the expected effect size was large and most patients included were not anaemic [reference] . . .

[Need for a trial]: To date, clinical practice guidelines are based on limited evidence and do not provide clear recommendations regarding RBC transfusion in TBI.[references] As a result, transfusion practices vary greatly within and between centres [references]; many clinicians extrapolate the evidence supporting the non-inferiority of a restrictive strategy in critically ill patients without TBI [references] while others advocate for a liberal transfusion strategy pending stronger evidence to support this practice [reference].”^139^

#### Explanation

The ethical and scientific justification for a trial depends on the uncertainty of the benefit or harm of the intervention to be tested. This uncertainty depends in turn on what is known on the topic before the trial commences. The background section of a protocol should summarise the relevance of the research question, justify the need for the trial in the context of available evidence, and present any available preliminary data on the potential effects of the interventions (benefits and harms), thus reporting a rationale for the trial.

This information is particularly important to the trial participants as it provides motivation for contributing to the trial.^140^ It is also relevant to funders, research ethics committees or institutional review boards, and other groups who evaluate the scientific and ethical basis for trial approval. The background should also present a plausible explanation of how the intervention might work. Understanding the rationale or theory underpinning an intervention helps readers to understand which aspects or components are likely to be essential to its efficacy or harm, and which are likely to be incidental.^24^

Reviews of two samples of 108 and 292 trial protocols from 2016 found that 44% and 26%, respectively, justified the research question.^9^ ^10^

The World Medical Association’s Declaration of Helsinki states that biomedical research involving people should be based on a thorough knowledge of the scientific literature, as it is unethical to expose humans unnecessarily to the risks of research.^2^ To place the trial in the context of available evidence, it is strongly recommended that an up-to-date, systematic review of relevant trials be summarised and cited in the protocol, or , in the absence of a published review, that the protocol authors systematically identify and summarise previous trials.^141^ ^142^ This evidence can also help researchers to optimise the usefulness of the new trial by informing design aspects such as outcome definition and sample size.^143^ ^144^ ^145^

Several funders request this background information in grant applications.^146^ ^147^ Failure to review the cumulative evidence can lead to unnecessary and wasteful duplication of research^148^ or to trial participants being deprived of effective interventions or exposed to harmful interventions.^144^ ^149^ ^150^ ^151^ Previous reviews of trial protocols have found that many do not cite the systematic reviews or trials that exist on the same topic.^152^ ^153^ ^154^

#### Summary of key elements to address

- Importance of the research question
- Why a new trial is needed in the context of available evidence:
  - explanation of how the intervention might work;
  - pretrial evidence of the benefits and harms of the interventions; and
  - reference to systematic review(s) of relevant trials; if none is available, a summary of relevant trials based on a systematic search.

### Item 9b: Explanation for choice of comparator

#### Examples

“Control intervention: restrictive transfusion strategy

Patients in the restrictive transfusion strategy group receive an RBC [red blood cell] transfusion only if their Hb [haemoglobin] is ≤70 g/L. We have chosen this threshold because it is the most studied restrictive RBC transfusion threshold [references] and reflects the current standard of care in non-bleeding critically ill patients without neurological or coronary artery diseases.[reference] It also is a frequently used and accepted threshold for clinicians who care for brain-injured patients.[reference]”^139^

“The control group will receive placebo. There is no current data on the efficacy of ivermectin against the virus in vivo; therefore the use of placebo in the control group is ethically justified. Participants of the study will have non-complicated COVID-19 and will not have risk factors to develop severe disease; thus they would not be receiving any alternative treatment for the disease.”^155^

#### Explanation

The choice of comparator has important implications for trial ethics, recruitment, results, and interpretation. In trials comparing an intervention with an active comparator, placebo, or usual care, a clear description of the rationale for the comparator will facilitate understanding of its appropriateness.^156^ ^157^ For example, a trial in which the comparator group receives an inappropriately low dose of an active drug will overestimate the relative efficacy of the study intervention in clinical practice; conversely, an inappropriately high dose in the comparator group will lead to an underestimate of the relative harms of the study intervention.^157^ ^158^ The choice of comparator should not deprive trial participants of an effective treatment if one is available. In a study of hypothetical decision making in doctors, around half indicated that they would enrol a patient in a randomised trial of biologics for rheumatoid arthritis despite considering the comparator inappropriate in the context of usual care.^159^

Reviews of two samples of 108 and 292 trial protocols from 2016 found that 76% and 83%, respectively, explained the choice of comparator.^9^ ^10^

What constitutes usual care differs substantially across clinical settings. The explanation for choosing a usual care comparator should be complemented by a description of the content of usual care (item 15a).

The appropriateness of using comparator groups only receiving placebo has been the subject of extensive debate and merits careful consideration of the existence of other effective treatments, the potential risks to trial participants, and the need for assay sensitivity—that is, the ability to distinguish an effective intervention from less effective or ineffective interventions.^160^ In addition, surveys have shown that a potential barrier to trial participation is the possibility of being allocated a placebo only or active comparator intervention that is perceived to be less desirable than the study intervention.^140^

If a placebo comparator group is planned, its type and components should be reported.^161^ Surgical placebo interventions require additional ethical and methodological considerations, as detailed by the Applying Surgical Placebo in Randomised Evaluations (ASPIRE) guidelines.^162^

#### Summary of key elements to address

- Why a particular comparator group was chosen
- Whether the comparator represents standard of care.

### Item 10: Specific objectives related to benefits and harms

#### Examples

“The primary objective of this trial is to assess the effect of primary antibiotic prophylaxis with co-trimoxazole on overall survival compared to placebo in adults with cirrhosis and ascites, utilising a treatment policy estimand. Key secondary objectives include assessing the incidence of SBP, hospital admissions, Clostridium difficile (C. difficile)-associated diarrhoea and antimicrobial resistance, cost-effectiveness, and incidence of cirrhosis-related events, liver transplantation and treatment-related serious adverse events.”^163^

“5 Objectives

5.1 Efficacy

The objective is to assess the ability of day-and-night hybrid closed loop glucose control to maintain CGM [continuous glucose monitoring] glucose levels within the target range of 3.9 to 10 mmol/l (70 to 180 mg/dl) in comparison to sensor augmented pump therapy in young children with type 1 diabetes.

5.2 Safety

The objective is to evaluate the safety of day-and-night hybrid closed loop glucose control, in terms of frequency and severity of hypoglycaemia, as defined by International Society for Paediatric and Adolescent Diabetes, frequency of diabetic ketoacidosis (DKA), and nature and severity of other adverse events.

5.3 Utility

The objective is to determine the acceptability, duration and frequency of use of the closed loop system in this population. A series of questionnaires will be given to parents/guardians at the end of each intervention arm.

5.4 Human Factors

The objective is to assess emotional and behavioural characteristics of participating subjects and family members and their response to the closed loop system and clinical trial using quantitative (validated surveys) and qualitative data (interviews).

5.5 Health Economics

The objective is to perform a cost utility analysis on the benefits of closed loop insulin delivery to inform reimbursement decision-making.”^164^

#### Explanation

The study objective reflects the scientific question to be answered by the trial and defines its purpose and scope, with profound implications for many other aspects of the trial, such as the design (item 12) and analysis (item 27).

The PICO format to formulate the trial objective is often used. This entails defining the participant population (P), intervention (I), comparator (C), and outcomes (O) of main interest.^165^ PICO is sometimes styled as PICOTS to include the timeframe and setting.

Protocol authors should further report whether the intention of the trial is to evaluate potential benefits and harms,^166^ and whether the aim is to assess superiority of the intervention or non-inferiority or equivalence^167^ versus the comparator. For example, the sample size calculation and statistical analyses for superiority trials will differ from those of non-inferiority trials.

Authors should also indicate which treatment effect they are planning to investigate—for example, the effect of assignment to the intervention irrespective of adherence, or the effect of adhering to the intervention (see box 1). They should also report whether the trial is intended to provide preliminary data (a pilot or feasibility trial) or confirmatory results.

Box 1 **Estimands**

Concerns have been raised that the precise research questions that randomised trials are intended to answer are often unclear.^168^ In particular, ambiguity often exists around the handling of events that occur after randomisation (termed intercurrent events). Research objectives can be specified using an estimands framework to improve clarity. We provide a brief overview of estimands and introduce terminology so this framework can be applied and reported if used. A more detailed primer on the estimand framework, which provides practical guidance on estimands in studies of healthcare interventions, can be found elsewhere.^169^

The European Medicines Agency^170^ defines an estimand as “a precise description of the treatment effect reflecting the clinical question posed by a given clinical trial objective.” The estimands framework provides a structured description of the treatment effect in an attempt to bring clarity to specifying the research question, which can be used to guide the study design, data collection, and statistical analysis methods. Briefly, an estimand comprises five key attributes: population, treatment conditions, endpoint, summary measure, and handling of intercurrent events (table 3 and table 4). A separate estimand should be defined for each study outcome, and for some outcomes, more than one estimand may be defined.

**Table 3:** Five key attributes of the estimands framework*

Attribute	Definition
Population	Patients for whom researchers want to estimate the treatment effect
Treatment conditions	Different intervention strategies being compared in the treatment effect definition
Endpoint	Outcome for each participant that is used in the treatment effect definition
Summary measure	Method used to summarise and compare the endpoint between treatment conditions (eg, risk ratio, odds ratio)
Handling of intercurrent events	Strategies used to handle each intercurrent event† in the treatment effect definition; different strategies could be used for different types of intercurrent events

* Adapted from Kahan et al.^169^

† Intercurrent events are post-baseline events (or post-randomisation events in randomised trials) that affect the interpretation or existence of outcome data. These events often affect receipt of treatment (eg, treatment switching or treatment discontinuation) or preclude existence of the outcome (eg, death, if it is not defined as part of the outcome). The European Medicines Agency outlines five strategies for handling intercurrent events, which are at the core of the estimands framework (see Kahan et al,^169^ European Medicines Agency,^170^ and table 4).

**Table 4:** Strategies for handling intercurrent events*

Strategy	Description
Treatment policy	The occurrence of the intercurrent event is considered irrelevant in defining the treatment effect of interest: the value for the outcome of interest is used regardless of whether the intercurrent event occurs
Hypothetical	The treatment effect in a scenario where the intercurrent event did not occur is of interest
Composite	The intercurrent event is incorporated into the outcome definition
While receiving treatment	The outcome before the occurrence of the intercurrent event is of interest
Principal stratum	The outcome in a subpopulation of patients who would not (or would) experience the intercurrent event is of interest

Although the terminology surrounding estimands may be new to some investigators, the concepts defined by the attributes and strategies of the framework are not new. Several reporting guidelines have recently included estimands in their recommendations.^171^ ^172^ If the estimands framework has been used to design the trial and data collection or to inform the statistical analysis (by choosing appropriate methods), then this should be clearly reported within the manuscript.

* Adapted from Kahan et al.^169^

If authors are planning a trial that involves readjusting the objective during the trial, such as in some platform trials or basket trials,^173^ ^174^ this should be reported. Trials can be designed to study the effect of the intervention under different conditions, often described on a spectrum from ideal conditions (explanatory trial) to routine clinical care conditions (pragmatic trial).^175^

The objectives should generally be phrased using neutral wording, such as “to compare the effect of treatment A versus treatment B on outcome X for persons with condition Y,” rather than in terms of a particular direction of effect.^176^ For multi-arm trials, the objectives should clarify which treatment group comparisons are of interest (eg, A *v* B, A *v* C).

Reviews of two samples of 108 and 292 trial protocols from 2016 found that 91% and 94%, respectively, described the specific objectives of the trial.^9^ ^10^

Recently, some trials are being designed using the estimands framework to define the research question and trial objectives.^177^ Box 1 presents the key concepts of this framework.

#### Summary of key elements to address

- Trial objectives related to the benefits and harms, including the:
  - participants;
  - intervention;
  - comparator;
  - primary outcome or outcomes; and
  - time point of main interest.
- Description of trial estimand or estimands, as appropriate.

### Item 11: Details of, or plans for, patient or public involvement in the design, conduct, and reporting of the trial

#### Example

“The study involved consumer representatives (n=2) in all aspects of the study design providing input to the processes and assessments of this research project. The Consumer and Community Health Research Network, through The Consumer and Community Involvement Coordinator (Mr BH) based at Edith Cowan University, assisted our Involvement Strategy by supporting us to find the most appropriate consumers for this work. The consumers were women over the age of 65 years who have attended five meetings, including four visits to the Medical Research Foundation and one visit to the Bone Density Department, over the last 12 months, prior to the start of the study. The women contributed to the design of the study, the informational material (results letter, handouts and videos) which supported the intervention and gave feedback about the burden of the intervention from the patient’s perspective (including clinical assessments and online questionnaires). The two women from the community involved in this research provided feedback on their own clinical results and contributed to the dissemination plan of the study by suggesting which information should be provided and how it should be presented (graphic representation of scan results, providing feedback on the wording of all documents and questionnaires provided). The consumers were provided with a AU$30 gift card for each hour involved in the study.”^178^

#### Explanation

Patient and public involvement in health research entails collaborating or partnering with patients and members of the public to design, conduct, interpret, or disseminate research: the research is done by or with patients and the public rather than for, at, or about them.^179^ ^180^ ^181^ Importantly, patient and public involvement is distinct from including patients or members of the public in a trial as participants.

The terminology used differs internationally—for example, such activity is commonly called patient and public involvement in the UK, whereas patient engagement is more common in mainland Europe and North America, community and public engagement in Africa, and consumer involvement in Australia.^180^ ^181^ ^182^

Funding bodies are increasingly encouraging or requiring researchers to include patient and public contributors in grant applications.^179^ ^183^ ^184^ In articles published in *The BMJ* family journals, which require a patient and public involvement section, about 40% of these statements reported that such activities were conducted.^185^ Most of these trials included patient and public contributors at the trial design stage.^185^

Input from the patient and public contributors and partners^186^ can help researchers to identify and prioritise research topics and questions, identify relevant outcome measures, boost recruitment and retention, improve trial design and tools, and improve the ethical acceptability of trial protocols to potential participants.^186^ ^187^ ^188^ Public involvement in other types of health research has been shown to help researchers to engage under-served populations and recruit diverse participant groups.^186^

Transparent reporting of patient and public contributors in a trial protocol is essential to allow interested parties, including potential participants and trial staff, research ethics committees or institutional review boards, and funders, to appraise the importance and ethical foundation of the trial.

We recommend that protocol authors report whether, and, if so, how patient and public involvement influenced the development of the protocol (for example, the number of patient representatives, their background, and their role), and describe any plans for similar involvement during the conduct and reporting of the trial. The GRIPP2 (Guidance for Reporting Involvement of Patients and the Public) checklist provides more in-depth guidance.^189^

#### Summary of key elements to address

- Planned methods of patient and public involvement at the different study stages (eg, design, conduct, reporting)
- Who will be involved (eg, patients, carers, members of the public)
- If no patient or public involvement planned, this should be stated.

### Item 12: Description of trial design including type of trial (eg, parallel group, crossover), allocation ratio, and framework (eg, superiority, equivalence, non-inferiority, exploratory)

#### Example

“The MYOMEX-2 trial is an open-label, multicentre, non-inferiority randomized controlled trial . . . Patients are randomised 2:1 in a parallel group design between two treatment arms: 119 patients in the UPA [ulipristal acetate] group and 60 patients in the surgery group.”^190^

#### Explanation

By “trial design” we mean the core characteristics of the type of a randomised trial planned (eg, parallel group or crossover) and the conceptual framework (eg, superiority, equivalence, or non-inferiority). Other specific aspects of the trial design, including details of randomisation and blinding, are dealt with elsewhere in the SPIRIT checklist.

SPIRIT 2025 focuses primarily on trials with participants individually randomised to one of two parallel groups. Although most published trials have such a design,^191^ the main alternative designs are multi-arm parallel,^192^ factorial,^193^ crossover,^194^ and cluster.^195^ The reporting of the planned unit of randomisation (eg, patient, clinic, lesion) is important. Most trials are designed to identify the superiority of an intervention, whereas others are designed to assess non-inferiority or equivalence.^196^ It is important that researchers clearly describe these aspects of their trial.

A review of 1000 randomised trials indexed in PubMed in 2016 showed that 85% had a parallel group design, whereas the other main designs were crossover (7%) and cluster (5%). Of the 1000 trials, 88% had two groups, 8% had three groups, and 4% had four or more groups.^191^

If a less common design is employed, authors should explain their choice, especially as such designs might imply the need for a larger sample size or more complex analysis and interpretation. For example, factorial and non-inferiority trials can involve more complex methods, analyses, and interpretations than parallel group superiority trials.^193^ ^197^ In addition, the interpretation of trial results in published reports is not always consistent with the prespecified conceptual framework,^198^ ^199^ ^200^ especially among reports claiming post hoc equivalence based on a failure to show superiority, rather than on a specific test of equivalence.^198^ Although most trials use equal randomisation (eg, 1:1 for two groups), it is helpful to explicitly state the allocation ratio.

#### Summary of key elements to address

- Type of trial design (eg, parallel group)
- Conceptual framework (eg, superiority, non-inferiority, or equivalence)
- Unit of randomisation (eg, individual participant)
- Allocation ratio (eg, 1:1).

### Item 13: Settings (eg, community, hospital) and locations (eg, countries, sites) where the trial will be conducted

#### Examples

“The trial is based in Kaffrine District, Senegal. The total population is 257 696 inhabitants. The population of children under 23 month is 17 780, and the prevalence of low birth weight (BW) is 17%. Data from 2015 indicated a prevalence of stunted children under five of 26.8%.[reference] The district has 28 health posts or clinics and 32 health huts administered by the Kaffrine Health Centre. The AASH [Action Against Stunting Hub] will recruit participants in seven clinics, and women participating in the AASH study will be targeted for the recruitment of their newborns in the SENGSYN study.”^201^

“Study setting: Healthy pregnant individuals registered to deliver at either St. Paul’s Hospital or BC Women’s Hospital, Vancouver, British Columbia, Canada, and under the care of a regulated maternity care provider (midwife, obstetrician or family doctor) will be invited to participate in the study . . . Those registered but who ultimately deliver outside of either hospital will still be included in the study.”^202^

#### Explanation

Along with the eligibility criteria for participants (item 14) and the description of the intervention and comparator (item 15), information on the settings and locations where the trial will be conducted is crucial to assess the generalisability of the trial.^203^ The environment in which the trial will be conducted may differ considerably from the setting in which the trial’s results are later used to guide practice and policy.

Reviews of two samples of 108 and 292 trial protocols from 2016 found that 42% and 56%, respectively, listed the countries where data were to be collected, 40% and 44% reported the location of participant recruitment, and 90% and 91% described the setting of intervention delivery.^9^ ^10^

Authors of protocols should report the number and type of settings and describe the type of care providers involved. They should report the locations in which the trial will be carried out, including the country, city if applicable, and immediate environment (eg, community primary care practice, hospital outpatient clinic, inpatient unit). It should be particularly clear whether the trial will be carried out in one or more sites (ie, single centre versus multicentre trial).

#### Summary of key elements to address

- Setting of participant recruitment (eg, primary or tertiary care, outpatient community or hospital clinic, inpatient unit)
- Location(s) where the trial will be carried out (eg, country, city)
- Planned number of sites.

### Item 14a: Eligibility criteria for participants

#### Example

“Patients should meet the following inclusion criteria: (1) KOA (knee osteoarthritis) according to the clinical American College of Rheumatology (ACR) criteria.[references] The clinical ACR criteria [reference] for KOA are: knee pain and at least three of the six following features: age ≥50, morning stiffness <30 min, crepitus, bony tenderness, bony enlargement, no palpable warmth. KOA will be confirmed with radiographs, including anterior-posterior and medio-lateral radiographs for imaging of the tibiofemoral joint, and an axial view for imaging of the patellofemoral joint. Patients with tibiofemoral (and patellofemoral) OA will be included. Kellgren and Lawrence (K&L) grading system for OA [references] will be applied, with K&L grade 2 or higher defined as OA; radiographic KOA is defined as definite osteophytes and possible joint space narrowing [references]; (2) pain, nominated by the patient as three or higher on a visual analogue scale on most days of the last 3 months [reference]; (3) aged ≥50 years.

Exclusion criteria are:

- Treatment with exercise therapy or joint infiltrations (eg, corticosteroids, hyaluronic acid) in the preceding 6 months.
- Being on a waiting list for knee replacement.
- Any contra-indication for exercise therapy as established by the treating physician.
- Corticosteroid infiltrations in the last 6 months.
- Cognitive impairment (unable to understand the test instructions and/or Mini-Mental State Examination score <23/30).
- Unable to understand the Dutch language.
- Inflammation unrelated to OA (eg, due to acute or chronic infection) established by CRP >10 mg/L.
- Presence of a disorder (eg, cancer, fibromyalgia, rheumatoid arthritis) and/or medication (eg, opioids, immunotherapy, anti-epileptics) that influences pain and/or the immune system.”^204^

#### Explanation

The criteria for eligibility of participants in a trial are important, partly because they should reflect the aim of the trial and partly because they affect recruitment, attrition,^205^ ^206^ ^207^ ^208^ ^209^ ^210^ ^211^ outcome event rates,^212^ ^213^ and generalisability.^214^

Typical selection criteria relate to the nature and stage of the condition or disease being studied, the exclusion of individuals thought to be particularly vulnerable to harm from study participation, and issues required to ensure that the study satisfies legal and ethical norms.

Reviews of two samples of 108 and 292 trial protocols from 2016 found that patient eligibility criteria were reported in 100% of protocols.^9^ ^10^ The importance of transparent documentation is highlighted by evidence that the eligibility criteria listed in published trials often differ from those specified in the trial protocol.^215^ ^216^ For example, a systematic review of articles comparing protocols and published reports (most being clinical trials or systematic reviews) found discrepancies about eligibility criteria in 25% to 57% of studies.^64^

Certain eligibility criteria may warrant explicit justification in the protocol, particularly when they limit the trial sample to a narrow subset of the population.^214^ ^217^ The appropriateness of restrictive participant selection depends on the objectives of the trial.^218^ When trial participants differ substantially from the relevant clinical population, the trial results may not easily be extrapolated to routine clinical settings.^215^ ^219^

#### Summary of key elements to address

- Specific inclusion and exclusion criteria defining the trial population to be randomised.

### Item 14b: If applicable, eligibility criteria for sites and for individuals who will deliver the interventions (eg, surgeons, physiotherapists)

#### Example

“Surgeon eligibility: participating surgeons must either be a consultant orthopaedic surgeon or perform the procedure under direct consultant supervision. To deliver KR (knee replacement) within KARDS (Knee Arthroplasty versus Joint Distraction for Osteoarthritis), a surgeon must have performed ≥10 KRs in the past 12 months as the primary surgeon. To deliver KJD (knee joint distraction) within KARDS, they must have performed ≥10 external fixations during their career as the primary surgeon or completed a limb reconstruction fellowship.”^220^

#### Explanation

For all types of trials, it is important to plan the eligibility criteria for trial sites and to consider the characteristics of the treatment providers intended to deliver both the experimental and the comparator interventions. One reason for this is logistical, as the complexity of running a trial increases with the number of sites (so each site should preferably be able to include a reasonable number of trial participants). For many trials, however, it is also important to align the experience of the treatment providers in the trial with that of the expected treatment providers in routine clinical practice.

For some drug interventions it is important that treatment providers have specialist training or that the intervention is delivered in specialised settings, such as within oncology. More commonly, issues related to the training, qualifications, and experience of treatment providers and the setup of sites arise in trials of non-drug interventions. For trials of non-drug interventions, such as surgery or rehabilitation, it is often important to also report the eligibility criteria for sites where the interventions will be administered and for the individuals who will deliver the intervention.^221^ ^222^

Patient outcomes can be associated with hospital and care provider volume—that is, the number of patients seen by a care provider or treated in a hospital.^222^ A systematic review of 135 trials found that 71% observed a positive association between hospital volume and outcomes and 70% observed an association between care provider volume and outcomes.^223^ Different levels of expertise of care providers in each trial group can bias treatment effect estimates.^224^ Furthermore, a non-drug intervention might be found to be safe and effective in a trial performed in high volume sites by high volume care providers, but it might have different results in low volume sites. For example, in an analysis of Medicare National Claim files of 167 208 patients undergoing coronary stent surgery, patients treated by high volume doctors and at high volume centres experienced better outcomes compared with those treated by low volume doctors at low volume centres.^225^

Eligibility criteria for care providers and centres in protocols for non-drug trials are often poorly reported. Reviews of two samples of 108 and 292 trial protocols from 2016 found that eligibility criteria for centres (and criteria for those performing the intervention) were reported in 30% and 38% of protocols, respectively.^9^ ^10^

A description of care providers who will be involved in the trial, as well as details of the sites where participants will be treated, is essential to enable study staff to apply these criteria consistently throughout the trial. This information also helps readers appraise the risk of bias and the applicability of the trial results to other populations.^222^ Eligibility criteria for sites typically relate to site volume for the procedure under investigation, or to similar procedures. Eligibility of care providers might include professional qualifications, years in practice, number of interventions performed, skill as assessed by number and type of complication when performing the intervention, and specific training before trial initiation. Eligibility criteria should be justified as they influence the applicability of the trial results.

#### Summary of key elements to address

If applicable:

- eligibility criteria for sites (eg, site volume for surgical procedure); and
- eligibility criteria for individuals delivering the interventions (eg, surgeons, physiotherapists), such as professional qualifications, years in practice, skills, or validation of specific training before trial initiation.

### Item 15a: Intervention and comparator with sufficient details to allow replication including how, when, and by whom they will be administered. If relevant, where additional materials describing the intervention and comparator (eg, intervention manual) can be accessed

#### Examples

“Azithromycin, or Zithromax, is supplied as an oral suspension in bottles containing azithromycin dehydrate powder equivalent to 1200 mg per bottle and the following inactive ingredients: sucrose, tribasic anhydrous sodium phosphate, hydroxypropyl cellulose, xanthan gum; FD&C Red #40, and flavoring including spray dried artificial cherry, crème de vanilla, and banana. After constitution, a 5 mL suspension contains 200 mg of azithromycin. This study will utilize the European Union formulation of the drug, which is comparable to the United States formulation.

Azithromycin and placebo will be administered as a single oral 1.2 g dose . . . We propose providing a 1.2 g oral dose depending on immediate availability and formulation for azithromycin and placebo . . . 

The comparator group for this trial will be a masked placebo. We propose to use placebo due to the lack of safety and efficacy data for azithromycin.”^226^

“The Scleroderma Patient-centered Intervention Network COVID-19 Home-isolation Activities Together [SPIN-CHAT] Program is a brief group videoconference intervention that was developed based on best-practice principles for managing anxiety and worry [references] recommendations for maintaining mental health during COVID-19 [references] and input from the SPIN COVID-19 Patient Advisory Team. The intervention will be delivered 3 times per week for 4 weeks during the COVID-19 crisis in 60- to 90-min sessions. Each session will include 3 segments: (1) engagement via therapeutic recreation activities (20-30 min); (2) education on information management and anxiety management through psychological and other strategies (20-30 min); and (3) open discussion and social support (20–30 min). Each intervention group will be moderated by a member of the research team or by leaders who have been trained in our SPIN support group leader training program [references].The moderators will support participants to develop routines . . . Supervision and support of group moderators will be provided by a trained social worker with 28 years of total experience and over ten years of experience working with the systemic sclerosis [SSc] community. Educational segments in each session will be delivered by a research team member with experience and training related to the topic.

Leisure activities that will be done at the start of each session will include games (eg, Pictionary, charades, pub-style trivia), creative activities (eg, roll-a-story, where participants roll a dice . . .), . . .

There will be an initial program overview in the first session. Then, educational segment topics will include (1) healthy information management and social connection (session 2); (2) managing worry (sessions 3, 7, 11) (3) . . .

In the healthy information management and social connection segment, strategies will be provided and discussion will be facilitated on how to stay informed via accurate information sources while avoiding sensationalist . . .

The managing worry segments will include an overview of worry, including what it is, the difference between helpful and harmful anxiety or worry, how to identify triggers of worry, and strategies to manage worry, . . . Exercises will be done with the group to illustrate techniques. In the relaxation segments, an introduction to the purpose of relaxation techniques . . . Adaptations for participants with breathing or positioning limitations will be made.

The physical activity segments will include an overview of the physical and psychosocial benefits of physical activity for maintenance of health for chronic disease management, [references] including ... Participants will be guided through movement options for the home-based setting, including warm-ups, aerobic, and strength activities. Behaviour change techniques to foster building the habit of moving more at home will be taught, including goal-setting, scheduling, addressing barriers, and building social support.[references]

Activity engagement sessions will be guided by the leisure education content model.[reference] Sessions will involve interactive group discussion about the benefits of leisure engagement [reference] . . . Sessions will explore barriers to leisure for persons with SSc and include tips for finding leisure resources within the participants' own homes, both in-person and online.

Based on our previous experience [references] and consistent with previous trials of videoconference training,[references] 8 participants will be assigned to each training group to maximize effective interaction and participation. Sessions will be delivered using the GoToMeeting® videoconferencing platform, a high-performance platform that has been used successfully in similar applications [references] and in feasibility and full-scale trials of SPIN's support group leader training program.[references](. . .)

Participants assigned to the waitlist will receive notices and reminders to complete trial measures as part of the SPIN-COVID-19 Cohort. They will be contacted with information on intervention groups post-trial.”^227^

“Usual care is defined as ‘the wide range of care that is provided in a community whether it is adequate or not, without a normative judgment’ [reference]. To increase external validity and relevance of study findings to clinical practice, the study protocol does not restrict access to usual care, in line with our pragmatic study design [reference] and the possibility for heterogeneity of usual care treatments available for older people with frailty. For example, usual care at a personal level will depend on individual frailty, level of independence and social predicaments. It is likely to include GP [general practitioner] care, district nurse input, and home care packages, but usual care may also include the use of voluntary sector services, day centres, and respite care. Use of and referral to services (including other rehabilitation) will be recorded at baseline and follow-up assessments in both intervention and control groups.”^228^

#### Explanation

The protocol’s description of the essential characteristics of the trial intervention and comparator are important for correct implementation during the trial, and they are also important for healthcare providers, systematic reviewers, policy makers, and others who are interested in understanding, implementing, or evaluating the intervention.^229^

This principle applies to all types of interventions, but it is particularly true for complex interventions (eg, health service delivery) consisting of interconnected components that can vary between healthcare providers and settings.

Reviews of two samples of 108 and 292 trial protocols from 2016 found that key elements of drug treatments (ie, generic name, dose, and schedule of intervention) were reported in more than 90% of protocols.^9^ ^10^

Protocols should provide a comprehensive description of each intervention and comparator, including usual care or placebo as applicable. Key information includes the different components of the intervention or comparator; how, when, and for how long it will be administered; the intervention material—that is, any physical or informational materials used for the intervention or comparator (eg, instruction manual), including those provided to participants or used in intervention delivery or in training of intervention providers and where it can be accessed (eg, online appendix, URL); and the procedure for tailoring the intervention or comparator to individual participants (box 2).

Box 2 Examples of essential information to be reported for various types of interventions*

**Drug^24^**

Generic name

Manufacturer

Dose

Route of administration (eg, oral, intravenous)

Timing

Titration regimen if applicable

Duration of administration

Procedure for tailoring the intervention to individual participants

Conditions under which interventions will be withheld

Whether and how adherence of patients to the intervention will be assessed or enhanced

Any physical or informational materials used in the intervention and where the materials can be accessed

**Rehabilitation, behavioural treatment, education, or psychotherapy^221^**

Qualitative information

Theory or rationale for essential intervention elements

Content of each session

Mode of delivery (individual or group, face to face or remote)

Whether the treatment is supervised

The content of the information exchanged with participants

The materials used to give information

Procedure for tailoring the intervention to individual participants

Whether and how the interventions will be standardised

Background and expertise of individuals delivering the interventions

Whether and how adherence of individuals delivering the interventions to the protocol will be assessed or enhanced

Any physical or informational materials used in the intervention and where the materials can be accessed

Quantitative information

Intensity of intervention when appropriate

Number of sessions

Session schedule

Session duration

Duration of each main component of each session

Overall duration of the intervention

**Surgery, technical procedure, or implantable devices^221^**

Details relevant to preoperative care

Details relevant to intraoperative care

Configuration of any device

Details relevant to postoperative care

Procedure for tailoring the intervention to individual participants

Whether and how the interventions will be standardised

Background and expertise of individuals delivering the interventions

Whether and how adherence of individuals delivering the interventions to the protocol will be assessed or enhanced

Any physical or informational materials used in the intervention and where the materials can be accessed

*This list is not intended to be exhaustive; it is a starting point for authors to consider when reporting the intervention. Information is based on Hoffman et al^24^ and Boutron et al.^221^

If the comparator or intervention group is to receive a combination of treatments, the authors should provide a description of each treatment, an explanation of the order in which the treatments will be introduced or withdrawn, and the triggers for their introduction, when applicable.

Interventions that consist of usual care (also called standard of care) require further elaboration in the protocol, as this care can vary substantially across sites and patients, as well as over the duration of the trial. Furthermore, it is important to clarify whether the intervention group also receives usual care and what will differ between the groups.

Specific guidance has been developed to improve the reporting of interventions, particularly the Template for Intervention Description and Replication (TIDieR),^24^ TIDieR-Placebo,^230^ which applies to trial protocols (as well as trial reports). Although the CONSORT extensions for non-drug treatments^221^ were developed for trial reports, they may also inspire authors of trial protocols.

#### Summary of key elements to address

- Details of each intervention and comparator to allow replication, including:
  - components of the intervention and comparator;
  - how the intervention or comparator will be administered;
  - when and for how long the intervention or comparator will be administered;
  - any procedure for tailoring the intervention to individual participants; and
  - any physical or informational materials to be used as part of the intervention or comparator (eg, instruction manual) and where the materials will be made accessible.
- When comparator group is usual care:
  - description of usual care, and any plans to track and measure it during the trial; and
  - whether the intervention group will also receive usual care.

### Item 15b: Criteria for discontinuing or modifying allocated intervention/comparator for a trial participant (eg, drug dose change in response to harms, participant request, or improving/worsening disease)

#### Examples

“Guidelines for Delay, Reduction and/or Discontinuation of Study Medications

Dose modification for an individual subject is not permitted unless the following ensues. Modification of study medication is allowed in Arm 0 (treatment arm with enoxaparin) if the [creatinine clearance] (CrCl) falls <15ml/min. In that instance conversion to dose adjusted IV UFH [unfractionated heparin] is acceptable during the time that the CrCl remains <15ml/min. If the patient cannot be placed on UFH IV (difficult to obtain frequent aPTT [activated partial thromboplastin time] draws, etc), an acceptable alternative is the use of UFH SQ using the fixed-dose weight-adjusted FIDO regimen, 333U/kg SQ, followed by 250U/kg Q12 hours, without the need to obtain [activated partial thromboplastin time] aPTT monitoring. The investigator is then encouraged to convert back to treatment dose enoxaparin as per protocol once the CrCl ≥15ml/min. Modification is allowed in Arm 1 (prophylactic group) if the CrCl falls <15ml/min to use [unfractionated heparin] UFH up to 22 500 U daily (i.e. UFH 5000u SQ BID or TID or 7500IU SQ BID or TID). The investigator is then encouraged to convert back to prophylactic/intermediate dose enoxaparin as per protocol once the CrCl ≥15ml/min.”^231^

“5.4.1 Discontinuation of the product under investigation

During the research treatment phase, the participant may suspend the product under investigation at any time. Likewise, the investigator may interrupt the product under investigation whenever necessary, either due to an adverse event or to preserve the participant's safety.

Participants who discontinue treatment under investigation without an apparent reason after randomisation and before the completion of the study will be encouraged to return with the medication and continue the study as normal. If the treatment is discontinued, the patient will continue in the research for the collection of information regarding events of the composite outcome. These participants will be treated according to the standard of care . . .

6.6 Modification of drug dose

6.6.1 Adverse reactions when using medications . . .

The decision to temporarily suspend medication can be taken at any time by either the participant or the investigator. Whenever possible, the patient should return to use the products under investigation.”^232^

#### Explanation

During a clinical trial, situations can emerge that necessitate changes in, or the discontinuation of, the allocated intervention or comparator for a participant. These events can be caused by a variety of factors, including harms, improved health status, lack of efficacy, and withdrawal of participant consent.^233^ The protocol should predefine standardised criteria for guiding intervention modifications and discontinuations. This information could be particularly important to evaluate the risk of bias owing to deviations from the intended intervention or comparator^234^; an important domain of the risk of bias tool developed by the Cochrane Collaboration. Assessing this domain requires a clear understanding of deviations that occur as planned in the protocol and deviations that arise owing to the experimental context.

Reviews of two samples of 108 and 292 trial protocols from 2016 found that 76% and 82%, respectively, reported criteria for modification of interventions.^9^ ^10^ Regardless of any decision to modify or discontinue their assigned intervention or comparator, study participants should be retained in the trial whenever possible to enable the collection of follow-up data and prevent missing data (item 25b).^235^

#### Summary of key elements to address

- Criteria to guide modifications to trial intervention or comparator (eg, drug dose change in response to harms, participant request, or improving or worsening disease)
- Criteria to guide discontinuation of trial intervention or comparator.

### Item 15c: Strategies to improve adherence to intervention/comparator protocols, if applicable, and any procedures for monitoring adherence (eg, drug tablet return, sessions attended)

#### Examples

“Study site personnel will review dosing information with the patient (or legally authorized representative) on scheduled clinic visit days, providing instructions regarding dose, dose frequency and the number of tablets to be taken for each dose. Patients (or legally authorized representative) will be instructed to keep all unused containers (empty, partially used, and/or unopened) for accountability at the next scheduled clinic visits. A compliance check and tablet count will be performed by study personnel during clinic visits. Study site personnel will record compliance information in the eCRF [electronic Case Report Form].

Every effort should be made to ensure patients return to the clinic with their study drug containers/unused study drug at each study drug dispensation visit. Study site personnel should conduct a verbal review of dosing with the patient and document the discussion in the patient’s medical record. This may serve as source documentation for the purpose of entering dosing data in the appropriate eCRF.”^236^

“All group sessions will be video-recorded and audited to ensure adherence to the program by two members of the research team. We will use standard methods for evaluating intervention fidelity, including observation of entire sessions for a randomly selected sample of 25% of sessions. Members of the research team will evaluate adherence to each session's goals and content. Consistent with best-practice recommendations for assessing treatment fidelity, [reference] this will be done using a checklist based on a standardized format adapted for the specific components of the SPIN-CHAT Program. The checklist will include the main session components (engagement via therapeutic recreation activities; education on information management and anxiety management through psychological and other strategies; open discussion and social support), and, for the educational component, will include the specific topics to be covered in each session.”^227^

#### Explanation

Fidelity to the intervention or comparator protocol is defined as the extent to which the intervention or comparator is implemented by care providers, as planned in the protocol.^237^ Adherence to intervention or comparator protocols refers to the degree to which the behaviour of trial participants corresponds to the intervention or comparator assigned to them (eg, taking a drug, behavioural change, doing exercises).^238^

It is important to consider the research question (eg, explanatory or pragmatic) when standardising the intervention and comparator and assessing fidelity and adherence.^239^ ^240^ ^241^ In explanatory randomised trials, where the aim is to determine the treatment effect under ideal circumstances, the intervention is usually highly standardised, with close monitoring of fidelity and adherence and use of strategies to increase them. Although no consensus exists on the acceptable minimum fidelity and adherence level in explanatory randomised trials, low fidelity and adherence can have a substantial effect on statistical power and interpretation of results.

To overcome the effects of non-fidelity and non-adherence, many trials implement procedures and strategies for monitoring and improving adherence, and any such plans should be described in the protocol. Monitoring could, for example, rely on biologic markers, direct patient observation, patient interviews, patient diaries, adherence questionnaires, pill counts, electronic monitoring of package entry, or ingestible smart sensors.^242^

Although the many types of monitoring methods have limitations,^243^ they can inform the interpretation of the trial result. It may be desirable to select strategies that can be easily implemented in clinical practice so that the level of adherence in routine clinical settings is comparable to that observed in the trial.

In pragmatic randomised trials, which aim to determine the treatment effect in typical clinical settings, interventions are usually highly flexible, with less focus on measuring fidelity and adherence and less use of strategies to maintain or improve them. Nevertheless, assessing fidelity and adherence to the intervention or comparator, or at least recording the most important components, is necessary to understand what was administered to participants. This is particularly important for complex interventions when diversity in the implementation of the intervention or comparator is expected. For example, in a pragmatic trial assessing a surgical procedure where the procedure is left to surgeons’ choice, investigators should plan to systematically record key elements related to preoperative care, anaesthesia, the surgical approach, and postoperative care. This information is essential to provide a relevant description of the intervention that was provided when the trial was completed.

Reviews of two samples of 108 and 292 trial protocols from 2016 found that strategies to improve adherence were addressed in 86% to 90%, respectively.^9^ ^10^ Authors should describe whether, when, and how fidelity and adherence to the intervention or comparator protocol were assessed.^244^ They should prespecify what is considered an adequate level of intervention or comparator delivery as planned. Any procedure to enhance fidelity or adherence to the intervention or comparator should be described. For pragmatic trials of complex interventions or comparators, authors should indicate which components and aspects of the intervention or comparator will be recorded and how.

#### Summary of key elements to address

- Strategies for improving fidelity of care providers and adherence of participants to intervention or comparator protocols, if applicable
- When and how fidelity of care providers and adherence of participants to intervention or comparator protocols will be assessed, if applicable
- When appropriate, prespecified definition for classifying participants as being treated as planned or not.

### Item 15d: Concomitant care that is permitted or prohibited during the trial

#### Example

“6.2.1 Concomitant therapy

All medications, procedures, and significant non-drug therapies (including physical therapy and blood transfusions) administered after the participant was enrolled into the study must be recorded on the appropriate Case Report Forms.

Each concomitant drug must be individually assessed against all exclusion criteria/prohibited medication. If in doubt, the investigator should contact the Novartis medical monitor before randomising a participant or allowing a new medication to be started. If the participant is already enrolled, contact Novartis to determine if the participant should continue participation in the study.

The patient must be told to notify the Treating Physician about any new medications that he/she takes after the start of canakinumab.

6.2.1.1 Permitted concomitant therapy requiring caution and/or action

Patients in this study will be enrolled to canakinumab or placebo, in addition to SOC per local practice, which may include anti-viral treatment, corticosteroids and supportive care.

Immunomodulator (topical or inhaled) use for asthma and atopic dermatitis or corticosteroid use (per medical judgement) are not restricted.

Use of oral, injected or implanted hormonal methods of contraception are allowed while on canakinumab.

6.2.2 Prohibited medication

The following medications are prohibited:

- Up to Day 29, concomitant use of biologics including anakinra, tocilizumab, abatacept, rilonacept, rituximab and any other biologics (investigational or marketed) and TNF inhibitors including etanercept, adalimumab, infliximab and/or other TNF inhibitors (investigational or marketed).
- All investigational medications being used in an investigational trial.”^245^

#### Explanation

In a randomised trial, a key goal is to have comparable study groups that differ only by the intervention being evaluated. Bias can arise when the trial groups receive different concomitant care—for example, additional interventions that may affect trial outcomes.^246^

Trials may differ in what is meant by the intervention being evaluated. In some trials (with a marked explanatory approach), the intervention might be defined narrowly as the experimental intervention tested. In other trials (with a more pragmatic approach), the intervention might be defined as the experimental intervention plus allowed concomitant interventions and procedures, such as rescue interventions. Also, in some multicentre trials an intervention could be added to usual care that might differ considerably in content between centres (item 15a).

Reviews of two samples of 108 and 292 trial protocols from 2016 found that 69% and 81%, respectively, reported the concomitant care that was permitted.^9^ ^10^

The protocol should list the relevant concomitant care that is allowed (including rescue interventions) or prohibited during the conduct of the trial. Relevant concomitant care refers to concomitant care and interventions that could affect the trial outcome. Plans for recording any allowed and prohibited concomitant care during the trial should also be reported.

#### Summary of key elements to address

- Relevant concomitant care that is allowed (eg, rescue interventions) or prohibited during the trial
- Any plans to record concomitant care received, including usual care.

### Item 16: Primary and secondary outcomes, including the specific measurement variable (eg, systolic blood pressure), analysis metric (eg, change from baseline, final value, time to event), method of aggregation (eg, median, proportion), and time point for each outcome

#### Examples

“The primary endpoint is the difference in mean change of GELP [Genital Erosive Lichen Planus] scores from baseline (week 8) to week 32 between deucravacitinib and methotrexate treatment groups.

The secondary endpoints will explore mean changes in:

- Vulvar Quality of Life Index (VQLI) [Reference] at weeks 8, 24 and 32 . . .
- General Health Questionnaire-28 (GHQ-28) [Reference] at weeks 8, 24 and 32
- Physician Global Assessment (PGA) [Reference] at weeks 8, 24 and 32
- Patient Global Assessment (PtGA) [Reference] at weeks 8, 24 and 32”^247^

“We will use two primary outcome measures:

1. The patient’s global perceived effect (GPE) at 3 months after start of treatment measured on a 7-point Likert scale. The GPE scale will be dichotomized as “improved” (scores 1–2) or “unchanged/worse” (scores 3–7). GPE is recommended as a core outcome measure in pain studies, as it may cover additional aspects to pain relief and physical function that is important to the individual [Reference].
2. The proportion with a clinically important improvement at 3 months in function measured by the Patient-Specific Function Scale (PSFS; 0–10). An important improvement will be defined as 30% increase on PSFS. The PSFS will also be dichotomized. Percent changes in PSFS scores will be calculated by taking the actual change in score divided by the possible change, to account for baseline values.”^248^

#### Explanation

Trial outcomes are fundamental to the study design and interpretation of results. For a given intervention, an outcome can generally reflect benefit (efficacy) or harm (adverse effect). The outcome of main interest is designated as the primary outcome, which usually appears in the objectives (item 10) and is the basis of the sample size calculation (item 19). The remaining outcomes constitute secondary or other exploratory outcomes. The inclusion of validated patient reported outcomes is encouraged to reflect patient perspectives on their health status.^23^

It is recommended that one outcome is designated as primary. Although up to 38% of trials defined multiple primary outcomes,^249^ ^250^ ^251^ ^252^ this practice can introduce problems with multiplicity of analyses, selective reporting, and interpretation when results are inconsistent across outcomes.^253^ Problems also arise when trial protocols do not designate any primary outcome, as seen in half of protocols for a sample of trials published from 2002 to 2008,^254^ and in 18% and 25% of randomised trial protocols that received ethics approval in Switzerland and Denmark, respectively.^249^ ^255^ Furthermore, major discrepancies in the primary outcomes designated in protocols, registries, and regulatory submissions versus final trial publications are common. The discrepancies favour the reporting of statistically significant primary outcomes over non-significant ones, and they are often not acknowledged in final publications.^64^ ^256^ ^257^ ^258^ ^259^ Such bias can only be identified and deterred if trial outcomes are clearly defined a priori in the protocol, and if protocol information is made public.^78^ ^260^ ^261^ ^262^

The systematic development and adoption of a common set of key trial outcomes for a given health condition can help to deter selective reporting of outcomes and facilitate comparisons and pooling of results across trials in a meta-analysis,^263^ ^264^ helping to reduce research waste. The COMET (Core Outcome Measures in Effectiveness Trials) initiative aims to facilitate the development and application of such standardised sets of core outcomes for clinical trials of specific conditions.^265^

Reviews of two samples of 108 and 292 trial protocols from 2016 found that the specific measurement variable was reported in 89% to 97%, respectively, whereas fewer specified the analysis metric (83% to 90%) and time point of main interest (82% to 94%).^9^ ^10^ Similar findings were observed for a sample of protocols of published oncology trials.^266^

For each outcome, the trial protocol should define four components: the specific measurement variable, which corresponds to the data collected directly from trial participants (eg, Beck depression inventory score, all cause mortality); the participant level analysis metric, which corresponds to the format of the outcome data that will be used from each trial participant for analysis (eg, change from baseline, final value, time to event); the method of aggregation, which refers to the summary measure format for each study group (eg, mean, proportion of participants with score >2); and the measurement time point of interest for analysis.^251^ ^261^

It is also important to explain the rationale for the choice of trial outcomes, such as use of a surrogate outcome.^267^ An ideal outcome is valid, reproducible, relevant to the target population (eg, patients), and responsive to changes in the health condition being studied.^205^ The use of a dichotomous analysis metric compared with a continuous metric reduces statistical power^268^ ^269^ ^270^; and subjective outcomes are more prone to bias from inadequate blinding (ascertainment bias) and allocation concealment (selection bias) than objective outcomes.^271^

Individual components of any composite outcome should be clearly defined.^22^ Although composite outcomes increase event rates and statistical power, their relevance and interpretation can be unclear if the individual component outcomes vary greatly for event rates, direction of effect, importance to patients, or amount of missing data.

#### Summary of key elements to address

- Specification of which outcomes are primary and which are secondary
- Rationale for the choice of trial outcomes and whether they are part of a core outcome set
- For each outcome:
  - specific variable to be measured (eg, Beck depression inventory score, all cause mortality), with definition when relevant;
  - analysis metric for each participant (eg, change from baseline, end value, time to event);
  - summary measure for each study group (eg, mean, proportion with score >2); and
  - time point of interest for analysis (eg, three months).

### Item 17: How harms are defined and will be assessed (eg, systematically, non-systematically)

#### Example

“Those administering the study products, all other trial staff collecting safety and immunogenicity endpoints, and the participants and parents will be blind . . .

DEFINITIONS

Adverse event (AE)

Based on ICH-GCP E6 (R2), an AE in this trial is defined as any untoward medical occurrence in a participant administered a study product which does not necessarily have a causal relationship with the study product itself. An adverse event can therefore be any unfavourable and unintended sign (including an abnormal laboratory finding), symptom or disease temporally associated with the administration of the study product whether related to the study product or not. Symptoms, signs or conditions present at screening (visit 0) which do not change are not AE and will be recorded as part of the screening procedures. Any subsequent change in the severity of a symptom, sign or condition following screening will be recorded as AE.

Serious adverse event

An AE is defined as serious if it:

- Results in death
  Any deaths will be reported as grade 5 severity
- Is life threatening
  The term life-threatening in the definition of serious refers to an event in which the participant was at risk of death at the time of the event; it does not refer to an event which hypothetically might have caused death if it was more severe.
- Requires inpatient hospitalization or prolongation of existing hospitalization
- Results in persistent or significant disability/incapacity
- Is a congenital anomaly/birth defect

Important medical event

Important medical events that may not be immediately life threatening or result in death or hospitalization, but which may jeopardize the participant or may require intervention to prevent one of the other outcomes listed in the definition of serious should be reported in the same ways as serious adverse events.

Suspected unexpected serious adverse reaction

AE that are serious or are important medical events, that are judged to be related to study product administration, and that are unexpected based on the information contained in the reference safety information will be termed suspected unexpected serious adverse reactions (SUSARs)

SOLICITED ADVERSE EVENTS

The following solicited local and systemic adverse events will be collected and graded for severity based on the National Institutes of Health (NIH), Division of AIDS (DAIDS) Table for Grading the Severity of Adult and Paediatric adverse events (version 2.1. Jul 2017):

- Adult local: pain, redness/erythema, swelling/induration, pruritus
- Toddler and infant local: pain, redness/erythema, swelling/induration
- Adult systemic: acute allergic reactions (day 0 only), axillary temperature, vomiting, diarrhoea, headache, fatigue, myalgia, arthralgia and rash
- Toddler and infant systemic: acute allergic reactions (day 0 only), axillary temperature, vomiting, diarrhoea, irritability, drowsiness, appetite and rash

Solicited AE will be recorded on the day of study product administration (visit 1 — day 0) by the study clinician and daily through home visits conducted by trained field workers between day 1 and day 13 post-study product administration. Any local and systemic AE ongoing after day 13 will be recorded by the study clinician as an unsolicited AE at the day 14 clinic visit (visit 3). Any grade 3 solicited AE identified during home visits will prompt an unscheduled clinic visit and review by a clinical study clinician on the same day or within 24 h at the latest.

Solicited AE data from home visits will be reviewed daily by a study clinician. In addition, home visits will be spot checked by senior members of the field team to ensure the quality and consistency of findings.

UNSOLICITED ADVERSE EVENTS

Any event fulfilling the definition of an AE, but which is not reported based on the definition of solicited local and systemic AE will be reported as unsolicited AE. When possible, collections of individual signs and symptoms will be reported as the underlying clinical syndrome. For example, gastroenteritis should be reported rather than diarrhoea, vomiting and fever. If an underlying clinical syndrome is not apparent, symptoms and signs will be reported individually. Unsolicited AE will be coded by preferred term (PT) and primary system, order, class (SOC) for reporting according to the latest online version of the MedDRA®.

CLASSIFICATION OF ADVERSE EVENTS

Severity

Solicited local and systemic AE as well as unsolicited AE will be graded for severity based on the NIH DAIDS Table for Grading the Severity of Adult and Paediatric AE (version 2.1. Jul 2017) or, if not included, based on the criteria set out in Table 5. The highest severity grade applicable at any point during an illness will ultimately be reported. Any AE which results in death will be defined as severity grade 5.

Causality

Other than solicited local reactions which, by definition, are related to study product administration, other AEs will be assessed for relatedness to the study vaccine by a study clinician.

The relatedness of a particular AE will be assessed based on clinical judgment considering the timing of the event in relation to study product administration, the nature of the event, the presence or absence of other illnesses or conditions to explain the event and relevant background history and concomitant medication use.

Based on these assessments, the relationship between a given AE and study product will be defined as:

- Related: There is a reasonable possibility of a causal relationship between the AE and the study product administered. The AE is more likely to be explained by the administration of the study product than by another cause.
- Not related: There is not a reasonable possibility of a causal relationship between the AE and the study product administered. The AE is more likely to be explained by another cause.

Given the double-dummy design, at the time of the initial unblinded assessment of an AE, it will not be possible to establish whether systemic AE is related to the MRV-MNP [measles and rubella vaccine microneedle patches] or MRV-SC.

Expectedness

Expectedness, either ‘expected’ or ‘unexpected’, will be assessed for unsolicited related AE by the sponsor’s medical expert based on the latest Investigator’s Brochure for the MRV-MNP and summary of product characteristics (SmPC) for the SC injection. For systemic events, the SmPC will serve as the reference safety information for the purposes of assessing expectedness of any reactions to the MRV irrespective of administration methods. Therefore, any systemic reaction judged to be expected based on the SmPC for the MRV will also be defined as expected following MRV-MNP.

Outcome

The outcome of AE will be defined as resolved/recovered, resolved/recovered with sequelae, ongoing stable chronic condition, ongoing at end of the study visit, resulted in death and unknown.

Safety laboratory investigations

Screening (visit 0) and safety (visit 2 and visit 3 [adults only]) laboratory investigations will be performed in the MRCG CSD laboratories according to their Standard Operating Procedures (SOPs). The SOPs govern the processes of sample reception, sample processing and result reporting. The CSD biochemistry, haematology, microbiology and serology laboratories are all ISO15189 accredited and Good Clinical Laboratory Practice (GCLP) compliant.

All abnormal safety laboratories will be graded based on a locally appropriate grading scale and will be judged for clinical significance and relatedness. Abnormal safety laboratories will be repeated as clinically indicated.”^272^

#### Explanation

Evaluation of harms plays a key role in monitoring the wellbeing of participants during a trial and in enabling appropriate management of adverse events. Documentation of trial related adverse events also informs clinical practice and the conduct of ongoing and future studies. To better reflect the negative effects of interventions, use of the term harms is preferred over safety,^273^ although we recognise variations in the terminology used between regional and organisational practices.

An adverse event refers to an unwanted occurrence during the trial, which may or may not be causally related to the intervention or other aspects of trial participation.^273^ ^274^ This definition includes unfavourable changes in symptoms, signs, laboratory values, or health conditions. An adverse effect is a type of outcome that is attributed to being caused by the intervention. In the context of clinical trials, it can be difficult to accurately ascribe causation for an adverse event experienced by an individual participant.^275^

How harms outcomes are assessed will affect the results obtained.^276^ ^277^ ^278^ ^279^ Harms outcomes can be either systematically assessed (ie, solicited by active or targeted surveillance) or non-systematically assessed (ie, unsolicited or spontaneous declaration, passive surveillance). To increase statistical power, harms outcomes are sometimes grouped in a composite outcome (eg, cardiovascular events).

Specifying the methods of assessing, grouping, and analysing harms in the protocol helps to identify and deter the selective reporting of results in publications and registries.^280^ ^281^ Substantial discrepancies have been observed between protocol specified plans for the data collection and reporting of harms and what is described in publications of completed trials.^282^

The protocol should list the systematically and non-systematically assessed harms outcomes that will be collected, as well as time points of their assessment and overall surveillance timeframe. It is important to state who will perform the assessment, coding, severity grading, and grouping, and whether those individuals will be blinded to the allocated trial group.

For each systematically assessed harms outcome, the information outlined in item 16 should be reported, including the outcome definition, instrument used (eg, name of a validated questionnaire), analysis metric (eg, final value), method of aggregation (eg, proportion), and time point of interest for analysis. The data collection methods should also be described as outlined in item 25a.

For non-systematically assessed harms outcomes, authors of protocols should report how data will be collected (item 25a), including any standardised questions used to solicit information. It is important to describe the process for coding adverse events (eg, using standardised terminology) and grading their severity, including the coding system (eg, Medical Dictionary for Regulatory Activities) and severity grading system (eg, Common Terminology Criteria for Adverse Events).

Harms outcomes are often grouped by seriousness, severity, body system, discontinuation of interventions owing to harms, and attribution of causality.^276^ If aggregation of adverse events is planned, then protocol authors should define the grouping categories. If harms will be categorised as being causally related to the intervention or not, authors should describe the attribution methods, including who will make the causal attribution and whether they will be blinded to the assigned trial group.^283^

If applicable, the protocol should address how important adverse events will be reported to relevant groups (eg, sponsor, research ethics committee or institutional review board, data monitoring committee, regulatory agency), a process that is defined by local regulation.^284^ Key considerations determining the need to report an adverse event to external groups include severity, potential causality, and whether the adverse event represents an unexpected event.

#### Summary of key elements to address

For each systematically assessed harm (active or targeted surveillance):

- definition and measurement (eg, name of validated questionnaire);
- where appropriate, the metrics, method of aggregation, and time point of interest for analysis; and
- procedures for harms assessment, including:
  - who will do the assessment and whether they will be blinded to the allocated trial group; and
  - assessment time points and overall time period for recording harms.

For each non-systematically assessed harm (passive surveillance):

- how data will be collected;
- assessment time points and overall time period for recording harms; and
- process for coding each adverse event and grading its severity, including:
  - who will do the coding and severity grading and whether they will be blinded to the allocated trial group; and
  - which coding and severity grading systems will be used, if any.

For grouping of harms by seriousness, severity, body system, discontinuation of intervention (owing to harms), and causality:

- definitions of grouping categories; and
- who will do the grouping and whether they will be blinded to the allocated trial group.

If relevant:

- process of reporting important adverse events to applicable groups (eg, sponsor, regulator, data monitoring committee).

### Item 18: Time schedule of enrolment, interventions (including any run-ins and washouts), assessments, and visits for participants. A schematic diagram is highly recommended (see fig 1)

#### Example

**Fig 2:**
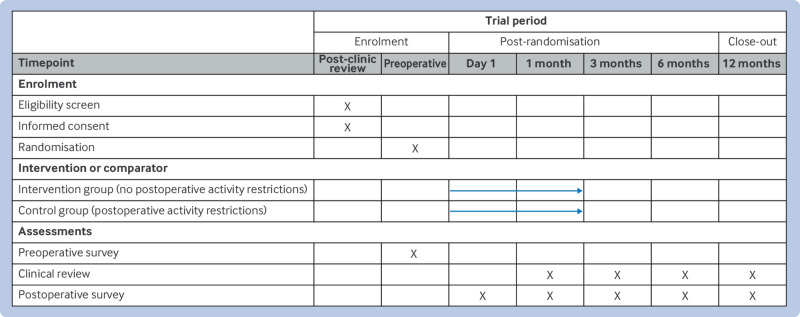
Participant schedule of enrolment, interventions, and assessments for a randomised trial of lumbar microdiscectomy and postoperative activity restrictions. Adapted from Daly et al^285^

“With the assistance of a blinded investigator, participants will complete online outcome questionnaires preoperatively, the day following surgery, and at home at one, three, six and 12 postoperative months. Questionnaires will include: 1. VAS lower back pain; 2. VAS leg pain; 3. ODI; 4. Current situation/patient satisfaction questionnaire; 5. SF-12/EuroQol (EQ-5D) (quality of life questionnaire). Patients will receive standard post-operative trial blinded neurosurgical outpatient review at approximately 30–60 days following surgery at trial sites. Teleconsultation will occur at the three, six and 12 months. Patients will only receive further neurosurgical outpatient review if clinically indicated . . . The participant timeline is illustrated below. (Figure 2)”^285^

#### Explanation

A concise timeline of the planned study visits, enrolment process, interventions, and assessments performed on participants can help to guide the conduct of a trial and enable external review of participant burden and feasibility. These factors can also affect the decision of potential investigators and participants to join the trial (item 20).^286^

A schematic diagram is highly recommended to efficiently present the overall schedule and time commitment for trial participants in each study group. Although various presentation formats exist, key information to convey includes the timing of each visit, starting from initial eligibility screening through to study close-out, time periods during which the trial intervention or comparator will be administered (including any run-ins and washout periods),^287^ and the procedures and assessments performed at each visit (with reference to specific data collection forms, if relevant).

#### Summary of key elements to address

Schematic diagram outlining the schedule and time commitment for trial participants, including:

- timeline of trial visits, starting from eligibility screening to trial close-out;
- timeline of interventions, including any run-in and washout periods; and
- procedures and assessments performed at each visit, referencing specific data collection forms, if relevant.

### Item 19: How sample size was determined, including all assumptions supporting the sample size calculation

#### Examples

“The sample size for this study is 1128 participants. This full trial sample size is based on the SD of the EQ-5D-5L at 4 months post surgery of 0.3 points [reference] and a minimal clinically important difference of 0.075 [reference] with 2-sided significance of 5% requiring 506 with the primary outcome for 80% power or 676 with the primary outcome for 90% power.

In this population, we expect considerable loss to follow-up. Previous WHiTE trials have indicated that these losses are due mainly to patients declining consent to further follow-up, incapacity, and death [references]. We are able to account for participants who have died in our primary outcome measure and have assumed that only 60% of recruited study participants will be available at the definitive endpoint at 4 months. With a significance level of 5%, this inflates the sample size to 844 for 80% power and 1128 for 90% power. Conservatively, we aim to randomise 1128 in order to ensure a minimum of 676 participants with the primary outcome which will ensure 90% power based on these assumptions.”^288^

“The trial is designed to detect a reduction in hospitalisation or mortality rate of 7.5% at 1 year in patients identified with cardiac dysfunction from 15% anticipated in patients randomised to the standard pathway [reference]. Given approximately one third of patients in both randomised arms are estimated to have cardiac dysfunction, a 7.5% reduction would be diluted to a 9% overall reduction in the enhanced pathway arm. To detect a reduction in events from 15% to 9% (equivalent to a HR equal to 0.58) using log-rank analysis with an overall type 1 error rate of 0.05 (two-sided analysis) and a power of 0.90 requires a total of 146 events to be observed in at least 1070 participants (nQuery Advisor assuming 18-month recruitment) inflated to 1200 in anticipation of minimal drop-out.”^289^

#### Explanation

The sample size calculation is a key component in the design of a randomised trial.^290^ ^291^ Sample size calculations need to balance ethical, logistical, clinical, and statistical considerations to ensure the scientific question can be reliably and precisely answered without unnecessarily exposing individuals to ineffective or harmful interventions. The sample size calculation is generally based on one primary outcome. For trials with more than one primary outcome, a separate calculation can be performed for each, and the largest sample size used.

The sample size should be sufficiently large to have a high probability (power) of detecting a clinically important difference of a prespecified magnitude that meets a criterion of statistical significance. The association between sample size and detectable difference is not linear: for small differences, enormous sample sizes are required for a trial to be sufficiently powered to detect them. A trial might knowingly be undertaken despite being underpowered, when the intent is for the trial to be incorporated into a prospectively planned meta-analysis.^292^

A complete description of the sample size calculation in the protocol enables an assessment of whether the trial will be adequately powered to detect a minimal clinically important difference.^293^ For transparency and reproducibility, the protocol should include the following (also see box 3): outcomes (item 16); values assumed for the outcome in each study group, such as the proportion with the event, or the measure of central tendency (eg, mean and standard deviation or median and interquartile ranges); statistical methods used to compare groups for outcomes (item 27a); α (type 1 error level); power; and calculated sample size for each group—assuming no loss of data and, if relevant, after any inflation for anticipated missing data (item 27c). Trial investigators are encouraged to also provide a rationale or reference for the outcome values assumed for each study group (thereby defining the target difference deemed important to detect), and to name the software used.^290^

Box 3 Reporting items for sample size calculation in the protocol of a randomised superiority trial*

**Recommended reporting items**

Core items

1. Primary outcome (and any other outcome on which the calculation is based); if a primary outcome is not used as the basis for the sample size calculation, state why
2. Statistical significance level and power
3. Express the target difference according to outcome type
  - Binary—state the target difference as an absolute or relative effect (or both), along with the intervention and control group proportions. If both an absolute and a relative difference are provided, clarify if either takes primacy in terms of the sample size calculation
  - Continuous—state the target mean difference, common standard deviation, and standardised effect size (mean difference divided by the standard deviation)
  - Time to event—state the target difference as an absolute or relative difference (or both); hazard ratio, the control group event proportion, planned length of follow-up, intervention and control group survival distributions, and accrual time (if assumptions regarding them are made). If both an absolute and a relative difference are provided for a particular time point, clarify if either takes primacy in terms of the sample size calculation
4. Allocation ratio; if an unequal ratio is used, state the reason
5. Sample size based on the assumptions listed above
  - Reference the formula or sample size calculation approach, if standard binary, continuous, or survival outcome formulas are not used. For a time-to-event outcome, state the number of events required
  - If any adjustments (eg, allowance for loss to follow-up, multiple testing) that alter the required sample size are incorporated, they should also be specified, referenced, and justified along with the final sample size
  - For alternative trial designs, additional input should be stated and justified. For example, for a cluster randomised trial (or an individually randomised trial with clustering), state the average cluster size and intracluster correlation coefficient(s). Variability in cluster size should be considered, and, if necessary, the coefficient of variation should be incorporated into the sample size calculation. Justification for the values chosen should be given
  - Provide details of any assessment of the sensitivity of the sample size to the inputs used
6. Underlying basis used for specifying the target difference (an important or realistic difference)
7. Explain the choice of target difference—specify and reference any formal method used or relevant previous research

*Adapted from Cook et al.^291^

The target difference in a superiority trial is the difference in the value of the primary outcome between the compared groups that the study is designed to detect. This reflects the two distinct concepts of statistical significance and clinical relevance. The target difference should ideally be the smallest clinically important difference (ie, the minimum clinically important difference),^294^ although some trials plan for a target difference that is realistically achievable.

The values of certain prespecified variables tend to be inappropriately inflated (eg, clinically important target difference) or underestimated (eg, standard deviation for continuous outcomes),^295^ leading to trials ultimately having less power than originally intended. References to support the sample size formula or approach should be given. When uncertainty about a sample size estimate is acknowledged, there are methods to re-estimate sample size.^296^ The protocol should describe the rationale, intended use, and details of such an adaptive design approach. If the sample size has been determined based on a series of simulations, it is essential to describe this method in enough detail to ensure a comparable level of transparency and evaluation.

Among randomised trial protocols that describe a sample size calculation, studies often do not state all components necessary to understand and reproduce the sample size (including the derivation of the target difference and the source of estimated values).^200^ ^297^ Reviews of two samples of 108 and 292 trial protocols from 2016 found that 99% reported the estimated sample size but only 57% to 78%, respectively, reported the assumed outcome values involved.^9^ ^10^ Also, a systematic review of articles comparing protocols and published reports (most being clinical trials or systematic reviews) found discrepancies about sample size in 26% to 44% of studies.^64^

For trial designs other than parallel group superiority trials, additional elements should be reported when describing the sample size calculation. For example, an estimate of the standard deviation of within person changes from baseline should be included for crossover trials,^298^ the intracluster correlation coefficient for cluster randomised trials,^195^ and the equivalence or non-inferiority margin for equivalence or non-inferiority trials, respectively.^196^ Such elements are often not described in final trial reports,^299^ ^300^ ^301^ ^302^ and they are infrequently specified in the protocol.^303^ For pilot or feasibility trials where sample size may not be guided by a formal sample size calculation, authors should report how the sample size was determined.^304^ ^305^ ^306^

#### Summary of key elements to address

For sample size calculations:

- primary outcome (and any other outcome) on which the calculations are based;
- outcome values (eg, proportion) assumed for each group, with rationale;
- target difference in outcome values between trial groups (including common standard deviation for continuous outcomes), with rationale;
- statistical significance level or α (type I) error;
- statistical power or β (type II) error;
- any upward adjustments (eg, accounting for missing data or non-adherence);
- target sample size for each trial group; and
- any software used.

### Item 20: Strategies for achieving adequate participant enrolment to reach target sample size

#### Examples

“Participants are recruited internally within the community-based FQHC [federally qualified health center]. The clinic data team regularly creates, updates, and shares a list of potentially eligible patients based on their recent HIV [human immunodeficiency virus] care history with the research team. Clinic staff also help to recruit from their patient population by reviewing clinic schedules weekly to identify participants with a pending appointment who might qualify based on the eligibility criteria for on-site recruitment. Other clinic staff, such as case managers and general medical staff, are informed of study details and eligibility to provide preliminary study information and refer potential participants. Research staff also work with other teams within the HIV Department to promote the study. The clinic’s community advisory board also serves as a resource for informing potential participants that the study is occurring, and study participants can also refer friends.”^307^

“Evidence-based site selection was used to confirm the eligibility of each centre to participate in the trial using volumes of endovascular repair of infra-renal and thoraco-abdominal aortic aneurysms listed on the National Vascular Registry as well as a record of satisfactory patient outcomes and strong clinical engagement. During the trial, the team will maintain regular contact with the sites, undertake regular site visits, and ensure there are adequate numbers of randomisers at sites and that Cydar EV is installed in as many rooms as required. These will be supplemented by in person local principal investigator and research nurse meetings where site teams can hear the experience of other sites and problems and tips and tricks to ensure strong participant recruitment can be shared.”^308^

#### Explanation

The main goal of recruitment is to meet the target sample size (item 19). However, difficulties in recruitment are common in clinical trials.^309^ ^310^ For example, a review of 151 trials from 2004 to 2016 funded by the UK Health Technology Assessment Programme found that 44% did not reach their recruitment targets.^311^ Inadequate enrolment reduces statistical power and can lead to early trial stoppage or to extensions resulting in delayed results and greater costs.

Strategies to promote adequate enrolment are thus important to consider during the planning of trials. Recruitment strategies can vary depending on the trial topic, context, and site. Different recruitment methods can substantially affect the number and type of trial participants recruited^206^ ^309^ ^312^ ^313^ and can incur different costs.^314^ ^315^ ^316^ Design issues such as the number and stringency of eligibility criteria will also directly affect the number of eligible trial participants.

Although most trials will use strategies to promote enrolment of participants, this information is often incompletely reported in trial protocols.^9^ ^10^ ^317^ For example, reviews of two samples of 108 and 292 trial protocols from 2016 found that less than 44% of protocols reported the location of recruitment, individuals to be recruited, or expected recruitment rate.^9^ ^10^

In the protocol, descriptions of where participants will be recruited (eg, primary care clinic, community), by whom (eg, surgeon), when (eg, time after diagnosis), and how (eg, advertisements, review of health records) can be helpful for assessing the feasibility of achieving the target sample size and the applicability of the trial results in practice. Other relevant information to explicitly provide in the protocol includes expected recruitment rates, duration of the recruitment period, plans to monitor recruitment during the trial, and any financial or non-financial incentives provided to trial investigators or participants for enrolment.

#### Summary of key elements to address

- Planned strategies to promote adequate enrolment (eg, advertisements, pre-screening of health records, reducing participant burden)
- Where participants will be recruited (eg, primary care clinic, community), by whom (eg, surgeon), and when (eg, time after diagnosis).

### Item 21a: Who will generate the random allocation sequence and the method used

#### Examples

“To ensure fairness, an independent statistician uses SPSS.25 software to generate randomized allocations for eligible patients . . . The statistician has no involvement in evaluating or executing the experiment.”^318^

“One of the leading investigators (TMS) will generate the allocation sequences using a random number generator in Excel.”^319^

“A statistician, not involved in the analysis of the trial results, will prepare the randomisation schedule. The randomisation schedule will be created using computer-generated random numbers before the first participant has been recruited, in a one-to-one ratio.”^320^

#### Explanation

Who generated the random allocation sequence is important for two main reasons. Firstly, someone, or some group, should take responsibility for this critical trial function. Secondly, providing information on the generator might help readers to evaluate whether anyone else had access to the allocation sequence during implementation (item 21b).

Participants should be assigned to comparison groups in the trial on the basis of a chance (random) process characterised by unpredictability. Successful randomisation in practice depends on two interrelated factors the adequate generation of an unpredictable allocation sequence and the concealment of that sequence until assignment occurs. A key problem is whether the schedule is known or can be predicted by the people involved in allocating participants to the comparison groups. The treatment allocation system should thus be set up so that the individual responsible for enrolling participants does not know in advance which group assignment the next participant will receive—a process termed allocation concealment. Proper allocation concealment shields knowledge of forthcoming assignments, whereas proper random sequences prevent correct anticipation of future assignments based on knowledge of past assignments.

Examples of adequate methods for the generation of random sequences include the use of a computerised random number generator or a random number table. If the random sequence is to be computer generated, we recommend specifying the software used. Randomisation decreases selection bias in allocation, helps to facilitate blinding after allocation, and enables the use of probability theory to test whether any difference in outcome between trial groups reflects chance.^321^

Use of terms such as randomisation without further elaboration in the protocol is not sufficient to describe the allocation process, as these terms have been used inappropriately to describe non-random, deterministic allocation methods such as alternation or allocation by date of birth.^322^ In general, these non-random allocation methods introduce selection bias and biased estimates of an intervention’s effect size.^323^ ^324^ ^325^ ^326^ ^327^ ^328^ Bias presumably arises from the inability to adequately conceal (item 22) these more predictable, non-random sequence generation systems.

The method of sequence generation was not described in three quarters of randomised trial protocols approved by a research ethics committee in Denmark in 1994-95, and a US cooperative cancer research group in 1968-2006.^329^ ^330^ More recent studies reported improved reporting. Reviews of two samples of 108 and 292 trial protocols from 2016 found that 75% and 61%, respectively, described the method of sequence generation.^9^ ^10^

#### Summary of key elements to address

- Who will generate the allocation sequence
- Method of sequence generation (eg, computerised random number generator)
- Any software used.

### Item 21b: Type of randomisation (simple or restricted) and details of any factors for stratification. To reduce predictability of a random sequence, other details of any planned restriction (eg, blocking) should be provided in a separate document that is unavailable to those who enrol participants or assign interventions

#### Examples

“On the day of surgery, participants will be randomized (1:1) to one of two treatment arms. Randomization will be stratified by hernia width measured during the procedure using the European Hernia Society Width categories (W1 <4cm, W2 4-10cm, W3 >10cm) [reference] to ensure equitable distribution of disease severity between the two treatment arms . . . The block sizes will not be known to the PIs. Randomization will take place through the Research Electronic Data Capture (REDCap™) system [reference].”^331^

“Participants will be randomly assigned to either control or experimental group with a 1:1 allocation as per a computer generated randomisation schedule stratified by site and the baseline score of the Action Arm Research Test (ARAT; <= 21 versus >21) using permuted blocks of random sizes. The block sizes will not be disclosed, to ensure concealment.”^332^

#### Explanation

Trial protocols must mention the type of randomisation planned (box 4), as this is the defining feature of a randomised trial. If stratification is to be used, the stratification categories (including relevant cut-off boundaries) should be reported (eg, recruitment site, sex, disease stage).

Box 4 **Randomisation and minimisation**

**Simple randomisation**

Randomisation based solely on a single, constant allocation ratio is known as simple randomisation.^323^ ^333^ ^334^ Simple randomisation with a 1:1 allocation ratio is analogous to an unbiased coin toss, although tossing a coin is not recommended for sequence generation. No other allocation approach, regardless of its real or supposed sophistication, surpasses the prevention of bias and unpredictability of simple randomisation.^321^ ^334^

**Restricted randomisation**

Any randomised approach that is not simple randomisation is restricted. Blocked randomisation is the most common form. Other methods, used less often, include replacement randomisation, biased coin, and urn randomisation.^321^ ^334^

**Blocked randomisation**

Blocked randomisation (also called permuted block randomisation) assures that study groups of about the same size will be generated when an allocation ratio of 1:1 is used. Blocking can also ensure close balance of the numbers in each group. After every block of eight participants, for example, four would have been allocated to each trial group.^335^ Improved balance comes at the cost of reducing the unpredictability of the sequence. Although the order of interventions varies randomly within each block, an individual running the trial could deduce some of the next treatment allocations if they discovered the block size.^336^ Blinding the interventions, using larger block sizes, and randomly varying the block size helps to avoid this problem.^321^ Fixed blocks of two or four are most problematic, as allocation might become predictable for a sizeable proportion of trial participants.

**Biased coin and urn randomisation**

Biased coin designs attain the similar objective as blocked designs without forcing strict equality. They therefore preserve much of the unpredictability associated with simple randomisation. Biased coin designs alter the allocation ratio during the trial to rectify imbalances that might be occurring.^321^ ^334^ Adaptive biased coin designs, such as the urn design, vary allocation ratios based on the magnitude of the imbalance. These approaches are, however, used infrequently.

**Stratified randomisation**

Stratification is used to ensure a good balance in characteristics of participants between each group. Without stratification, study groups might not be well matched for baseline characteristics, such as age and stage of disease, especially in small trials. Such imbalances can be avoided without sacrificing the advantages of randomisation. Stratified randomisation is achieved by performing a separate randomisation procedure within each of two or more strata of participants (eg, categories of age or baseline disease severity), ensuring that the numbers of participants receiving each intervention are closely balanced within each stratum. Stratification requires some form of restriction (eg, blocking within strata) to be effective. The number of strata should be limited to avoid over-stratification.^337^ Stratification by centre is common in multicentre trials.

**Minimisation**

Minimisation assures similar distribution of selected participant factors between study groups.^333^ ^338^ Randomisation lists are not set up in advance. The first participant is truly randomly allocated; for each subsequent participant, the treatment allocation that minimises the imbalance on the selected factors between groups at that time is identified. That allocation may then be used, or a choice may be made at random with a heavy weighting in favour of the intervention that would minimise imbalance (for example, with a probability of 0.8).^339^ The use of a random component is generally preferable.^340^ Minimisation has the advantage of creating small groups closely similar for specific desirable participant characteristics at all stages of the trial.

Minimisation offers the only acceptable alternative to randomisation, and some have argued that it is superior.^341^ Conversely, minimisation lacks the theoretical basis for eliminating bias on all known and unknown factors. Nevertheless, in general, trials that use minimisation are usually considered methodologically similar to randomised trials, even when a random element is not incorporated. For SPIRIT, minimisation is considered a restricted randomisation approach without any judgment as to whether it is superior or inferior to other restricted randomisation approaches.

SPIRIT=Standard Protocol Items: Recommendations for Interventional Trials.

When restricted randomisation is planned, details on restriction (including minimisation) should not appear in the main body of the protocol to reduce predictability of the random sequence (box 5). The details should instead be described in a separate document that is unavailable to trial implementers, such as recruiters, screeners, enrollers, and assigners. For blocked randomisation, this information would include details on how the blocks will be generated (eg, permuted blocks by a computer random number generator), the block size or sizes, and whether the block size will be fixed or randomly varied.

Box 5 **Need for a separate document to describe restricted randomisation**

If some type of restricted randomisation approach is to be used, in particular blocked randomisation or minimisation, then knowledge of the specific details could lead to bias.^342^ ^343^ For example, if the trial protocol for a two arm, parallel group trial with a 1:1 allocation ratio states that blocked randomisation will be used and the block size will be six, then trial implementers know that the intervention assignments will balance every six participants. Thus, if intervention assignments become known after assignment, knowing the block size will allow trial implementers to anticipate when equality of the sample sizes will arise. A sequence can be discerned from the pattern of past assignments, and then some future assignments could be accurately predicted. For example, if part of a sequence contained two As and three Bs, trial implementers would know that the last assignment in the sequence would be an A. If the first three assignments in a sequence contained three As, trial implementers would know that the last three assignments in that sequence would be three Bs. Selection bias could result, regardless of the effectiveness of allocation concealment (item 22).

Selection bias is mainly a problem in open label trials where everyone becomes aware of the intervention after assignment. It can also be a problem in trials where everyone is supposedly blinded (masked), but the blinding is ineffective, or the harms from the intervention provide clues such that treatments can be guessed.

We recommend that trial investigators do not provide full details of a restricted randomisation scheme (including minimisation) in the trial protocol. Knowledge of these details might undermine randomisation by facilitating deciphering of the allocation sequence. Instead, this specific information should be provided in a separate document with restricted access. However, simple randomisation procedures could be reported in detail in the protocol because simple randomisation is perfectly unpredictable.

Simple randomisation (unrestricted randomisation) can be specified in the main body of the protocol because it is perfectly unpredictable. Its use does not require a separate document.

#### Summary of key elements to address

- Type of randomisation: simple versus restricted (eg, blocked), fixed versus adaptive (eg, minimisation), and, when relevant, the reasons for such choices
- If applicable, factors (eg, recruitment site, sex, disease stage) to be used for stratification, including categories and relevant cut-off boundaries
- For restricted randomisation, aside from the above: all other details on restriction (including minimisation) should be provided in a separate document to reduce predictability of the random sequence.

### Item 22: Mechanism used to implement the random allocation sequence (eg, central computer/telephone; sequentially numbered, opaque, sealed containers), describing any steps to conceal the sequence until interventions are assigned

#### Examples

“The use of central web-based randomization with permutated blocks will ensure that individual surgeons and anesthesiologists cannot determine trial allocation ahead of time. Allocation to the experimental or control interventions will take place the day of surgery. The anesthesiologist will reveal the allocation once the patient is considered “anesthesia ready,” which implies that general anaesthesia has been administered, and all lines and catheters have been inserted. By revealing the allocation once the patient is in the operating room and under anaesthesia, we can ensure that clinical care will not be influenced by trial allocation.”^344^

“The adopted concealment mechanism of the allocation sequence will use sequentially numbered (#), opaque, sealed envelopes.”^345^

#### Explanation

Successful randomisation in practice depends on two interrelated factors: generation of an unpredictable allocation sequence (item 21), and concealment of that sequence until assignment irreversibly occurs.^336^ ^346^ The allocation concealment mechanism aims to prevent participants and recruiters from knowing the study group to which the next participant will be assigned.^336^ ^346^ Allocation concealment helps to ensure that a participant’s decision to provide informed consent, or a recruiter’s decision to enrol a participant, is not influenced by knowledge of the group to which they will be allocated if they join the trial. Allocation concealment should not be confused with blinding (masking) (item 24), which seeks to prevent ascertainment, performance, and attrition biases.

Without adequate allocation concealment, even random, unpredictable assignment sequences can be subverted.^336^ ^346^ ^347^ ^348^ For example, a common practice is to enclose assignments in sequentially numbered, sealed envelopes. If the envelopes are not opaque and the contents are visible when held up to a light source, or if the envelopes can be unsealed and resealed, then this method of allocation concealment can be corrupted.

Protocols should describe the planned allocation concealment mechanism in sufficient detail to enable assessment of its adequacy. In one study of randomised trial protocols in Denmark, more than half did not adequately describe allocation concealment methods.^329^ In contrast, central randomisation was stated as the allocation concealment method in all phase 3 trial protocols initiated in 1968-2003 by a cooperative cancer research group that used extensive protocol review processes.^330^ Improved protocol reporting was found more recently. Reviews of two samples of 108 and 292 trial protocols from 2016 found that 83% addressed the mechanism of allocation concealment.^9^ ^10^ Similar to failure of random sequence generation, inadequate or unclear allocation concealment in trial publications has been associated with inflated effect size estimates.^325^ ^327^ ^328^ ^336^ ^349^ ^350^

#### Summary of key elements to address

- How the individuals enrolling participants will be kept unaware of the next trial group assignment in the random sequence (not to be confused with blinding).

### Item 23: Whether the personnel who will enrol and those who will assign participants to the interventions will have access to the random allocation sequence

#### Examples

“The randomization is performed using the National Cancer Institute clinical trial randomization tool (https://ctrandomization.cancer.gov/). A minimisation method is used on the allocation process to ensure the required balance across the intervention and control groups. Results are sent to an independent researcher responsible for allocation that is not involved in the recruitment nor in the delivery of the intervention. The allocation is performed right before the beginning of the intervention of each group of randomised participants (in blocks), sending the results to a local researcher in a pre-printed Excel worksheet (Microsoft, Redmond, WA, USA). Randomisation and allocation of participants are carried out by different researchers.”^351^

“One of the leading investigators (TMS) will generate the allocation sequences using a random number generator in Excel. After eligibility is determined, the other leading investigator (AEP) will assign participants to condition (either intervention or wait-list control) according to those sequences and subsequently enroll participants in the study . . . The allocation sequence will be stored in a secure folder on a secure iRT [innovation Research & Training] server until interventions are assigned and will only be accessible to the leading investigator (TMS) who created the sequence. The other leading investigator (AEP), who assigns participants to interventions, will only see the assignments for participants ready to enroll, and will not be able to see the future allocation sequence.”^319^

“Randomization is performed by a statistician unrelated to the trial. The research assistant (RA) who is carrying out allocation will have no access to allocation sequence.”^352^

“The random sequence for trial group allocation in blocks will be generated by the P.I. [principal investigator] using an online tool [reference] . . . To prevent biased selection of patients, the PI will remotely screen for new patient admissions and alert the ICU [intensive care unit] staff for possible candidates. The patient’s attending physicians are responsible for applying protocol, deciding patient eligibility, and checking for inclusion/exclusion criteria and obtaining the informed consent. Once patient enrollment has been established, the attending physicians, who are blinded for the allocation blocks, will determine the corresponding stratification subgroup by using IAP [intra-abdominal pressure] and creatinine values and open the respective sealed envelope containing the group allocation.”^345^

“The randomization list will be computer-generated by the biostatistician and integrated into a REDCap randomization project. Members of the study staff will not have access to the sequence prior to assignment and will utilize the REDCap project to randomly assign participants to study arm.”^353^

#### Explanation

The process of randomising participants into a trial involves three steps: sequence generation, allocation concealment mechanism, and implementation. Investigators should strive for complete separation of the people involved with sequence generation and allocation concealment from the people involved in the implementation of assignments.

Failure to implement this separation might introduce bias. For example, the individual who generated the allocation sequence will likely have access to a copy of the sequence list and could consult it if they were enrolling and assigning participants in a trial. Thus, that individual could bias the enrolment or assignment process.

Authors of protocols should confirm complete separation of the people involved with sequence generation and allocation concealment from the people involved in the implementation of assignments, and they should describe how this separation was achieved. If complete separation did not occur, then authors should describe how the people involved in the implementation will be prevented from accessing the allocation sequence (eg, specifying that the allocation sequence will be locked in a secure location).

Reviews of two samples of 108 and 292 trial protocols from 2016 found that 73% and 56%, respectively, did not describe the individuals who would enrol or assign participants.^9^ ^10^

#### Summary of key elements to address

- Who will have access to the random allocation sequence
- Who will enrol participants
- Who will assign participants to interventions
- Whether the staff enrolling and assigning participants will have no access to the random allocation sequence

When individuals involved in sequence generation and allocation concealment are the same individuals involved in the implementation of assignment:

- how and where the random allocation list will be securely stored; and
- any mechanisms to prevent those responsible for enrolling and assigning participants from accessing the list.

### Item 24a: Who will be blinded after assignment to interventions (eg, participants, care providers, outcome assessors, data analysts)

#### Examples

“Thus, participants and care providers will be blinded to the intervention. All researchers involved in the trial are also blinded to the intervention allocation until the trial is completed. At the end of the study, researchers performing the statistical analysis will divide the participants into the coded groups and will be unaware regarding the intervention allocation.”^351^

“Trial participants, care providers, outcome assessors, and data analysts will be blinded to treatment allocation. Only the provider of the treatment and placebo packages will know the coding schemes for the corresponding packages and will not disclose this information until after trial completion.”^354^

“Given the open nature of the trial, the assigned interventions will be unblinded for the clinical staff and patient. Outcome assessment and data analysts will also be unblinded for patient’s group allocation given the objective character of the outcomes and the open trial intervention.”^345^

#### Explanation

Blinding (masking)—the process of keeping the study group assignment hidden after allocation—is commonly used to reduce the risk of bias in randomised trials.^355^ ^356^ Awareness of the intervention assigned to participants can introduce ascertainment bias in the measurement of outcomes, particularly subjective ones (eg, quality of life)^325^ ^356^ ^357^; performance bias in the decision to discontinue or modify study interventions (eg, dosing changes) (item 15b), concomitant interventions, or other aspects of care (item 15d)^358^; and exclusion or attrition bias in the decision to withdraw from the trial or to exclude a participant from the analysis.^359^ ^360^ We use the generally preferred term blinding but acknowledge that others prefer to use the term masking because of the association between blind and an ophthalmological condition or health outcome.^361^

Many groups can be blinded: trial participants, care providers, data collectors, outcome assessors, data analysts,^362^ and manuscript writers. Blinding of data monitoring committee members is generally discouraged.^363^

When blinding of trial participants and care providers is not possible owing to obvious differences between the interventions,^364^ ^365^ blinding of the outcome assessors can often still be implemented.^323^ The exception is in trials with patient or participant reported outcomes (eg, pain) where the patient is the outcome assessor.

It may also be possible to blind participants or trial staff to the study hypothesis in terms of which intervention is considered active. For example, in a trial evaluating light therapy for depression, participants were informed that the study involved testing two different forms of light therapy, whereas the true hypothesis was that bright blue light was considered potentially effective and that dim red light was considered placebo.^366^

Despite its importance, blinding has often been poorly described in trial protocols.^5^ Yet, reviews of two samples of 108 and 292 trial protocols from 2016 found that 82% to 94% of protocols addressed the blinding status of participants and care providers, and 68% to 70% reported the blinding status of outcome assessors.^9^ ^10^

The protocol should explicitly state who will be blinded to intervention groups—at a minimum, the blinding status of trial participants, care providers, and outcome assessors. Such a description is much preferred over the use of ambiguous terminology such as single blind or double blind.^367^ ^368^

#### Summary of key elements to address

- Who will be blinded to treatment assignments:
  - trial participants;
  - care providers (ie, those administering the intervention);
  - outcome assessors (ie, those who determine whether a participant experienced the outcome of interest), such as the participant (for patient reported outcomes), care provider, or independent observer; and
  - data analysts performing the statistical analysis.

### Item 24b: If blinded, how blinding will be achieved and description of the similarity of interventions

#### Examples

“Two capsules of the probiotic contain 5×107 CFU of Hafnia alvei HA4597™, 5 mg of zinc and 20 μg of chromium. The capsules are gastro-resistant which allows the ingredients to resist stomach acidity. A placebo is used as a comparator to determine if the probiotic effect is directly related with the strain (i.e. Hafnia alvei). The placebo product is indistinguishable in colour, smell, and taste from the active formulation, and has the same composition but without the live bacteria . . . The study products are packaged and blinded by an outsourced pharmacy. The package appearance of both products is identical.”^351^

“The loading infusion agents will be diluted in sterile, plastic, and opaque containers, and the continuous infusion agent will be prepared in a 50 mL syringe (all labeled “research agent”) . . . After endotracheal induction, patients in the high-dose TXA [Tranexamic acid] group will be administered a loading dose of TXA 20 mg/kg, followed by a continuous infusion of TXA at a rate of 5 mg/kg/ hour until dura closure. In the low-dose group, patients will receive a loading dose of TXA 20 mg/kg followed by a continuous infusion of normal saline. In the control group, patients will receive an identical volume of normal saline in the same setting.”^369^

#### Explanation

Achieving blinding of trial participants or staff requires adequate procedures for both induction and maintenance of blinding. Often, blinding of trial participants and intervention providers involves matching of the compared interventions—that is, producing two externally indistinguishable interventions, one experimental containing the causal component the trial is testing and one comparator without the component. Classically this is achieved by producing a placebo comparator that matches the experimental intervention—not only the format (eg, tablet) but also external characteristics (eg, colour, taste, size).^162^

In some trials that test two active interventions (for example, one as a tablet and the other as an injection) a double dummy procedure is used in which the compared interventions involve an experimental tablet with placebo injection versus placebo tablet with experimental injection. The conventional placebo used in most trials is matched on the external characteristics of the experimental intervention, but not to any discernible non-therapeutic effects of the experimental intervention. If such side effects are regarded as potentially important, some drug trials use an active placebo—that is, a placebo to which has been added a pharmacologically active substance that mimics (some) of the side effects of the experimental intervention.^370^ ^371^

Blinding of an external outcome assessor is almost always possible, even in situations where blinding of trial participants and intervention providers is not. This usually requires that the outcome assessor does not participate in patient care.

Maintaining blinding of participants, care providers, and outcome assessors can be challenging. Indeed, inadequate matching—that is, distinguishable differences in the physical properties of compared interventions—seems to be the main mechanism for unblinding in drug trials.^372^ Notably, inadequate matching related to discernible differences in colour and taste seem particularly problematic.^372^ The risk of unblinding is higher in trials designed such that patients are exposed to both the experimental and the comparator intervention, such as in crossover trials and parallel group trials with a placebo run-in period.

Another obvious example of the risk of unblinding is use of a fixed code (versus a unique code for each participant) to denote each trial group assignment (eg, A=experimental, B=comparator). In these circumstances, the unblinding of one participant could result in the inadvertent unblinding of many trial participants. The risk of unblinding of is high when outcome assessors interact closely (eg, during an interview) with unblinded patients. 

Some have suggested that the success of blinding should be formally tested at the end of the trial by asking key individuals in the trial to guess the study group assignment and comparing these responses to what would be expected by chance.^373^ However, it is unclear how best to interpret the results of such tests.^372^ ^374^ ^375^ ^376^

The reporting in trial protocols of plans for inducing and maintaining blinding was studied in a sample of protocols from 1994.^5^ Out of 55 protocols of trials planned as double blind, five (9%) provided no information beyond double blind, 25 (45%) reported a double dummy procedure, and 32 (58%) reported the compared interventions as being similar.

If trial researchers contend that the trial investigators, participants, and assessors will be blinded, then the protocol should provide information about the mechanism (eg, capsules, tablets, film) and similarity of treatment characteristics (eg, method of administration, appearance, smell, taste, use of special flavours to mask a distinctive taste). Furthermore, any strategies to reduce the potential for unblinding should be described in the protocol, such as pretrial testing of blinding procedures.^377^ We also encourage investigators to describe any procedures intended to reduce or evaluate the risk of compromised blinding,^372^ ^376^ including cases of overt unblinding.^372^ ^376^

#### Summary of key elements to address

For blinded trials:

- mechanism to establish blinding (eg, identical placebo, double dummy);
- any similarities or differences in characteristics (eg, appearance, taste) of the interventions being compared;
- any procedures intended to maintain blinding and reduce risk of unintentional unblinding; and
- any procedures intended to evaluate blinding procedures (eg, pretrial testing of blinding procedures).

### Item 24c: If blinded, circumstances under which unblinding is permissible, and procedure for revealing a participant’s allocated intervention during the trial

#### Examples

“We expect the need for emergency unblinding to be relatively rare. Nevertheless, we have the following procedure in case emergency unblinding is required: If unblinding is deemed to be necessary for the intraoperative event of significant safety concerns, the principal investigator will be notified immediately. If the principal investigator considers emergency unblinding necessary, a request will be directed to the responsible anesthesiologist. The requested information and details of emergency unblinding will be recorded in the case report form and well-documented. The actual allocation must not be disclosed to the participant or other trial personnel since the trial is placebo controlled-double-blind.”^369^

“This study defines three unblinding situations^1^: when data entry is completed,^2^ when statistical analysis is completed, and 3 when serious adverse events occur. All adverse events will be reported to the data and safety monitoring committee (DSMC), who will decide whether the patient’s participation needs to be discontinued and whether the patient should be unblinded immediately.”^378^

#### Explanation

Emergency unblinding reveals the assigned intervention of a trial participant, typically when the participant experiences potential harm from the intervention. It is important that emergency unblinding procedures are constrained to a single participant and that unblinding of additional participants is avoided (item 24b). In some cases (eg, minor, reversible harms), stopping the assigned intervention and then cautiously reintroducing it can avoid both unblinding and further harm.

Reviews of two samples of 108 and 292 trial protocols from 2016 found that 81% and 90%, respectively, addressed the conditions when unblinding was permissible.^9^ ^10^

A clear protocol description of the circumstances and procedures for emergency unblinding helps to prevent unnecessary unblinding, facilitates implementation by trial staff when indicated, and enables evaluation of the appropriateness of the planned procedures.

#### Summary of key elements to address

For blinded trials:

- circumstances under which unblinding is permissible during the trial (eg, to reduce immediate risk to a participant); and
- procedure for revealing a participant’s allocated intervention during the trial.

### Item 25a: Plans for assessment and collection of trial data, including any related processes to promote data quality (eg, duplicate measurements, training of assessors) and a description of trial instruments (eg, questionnaires, laboratory tests) along with their reliability and validity, if known. Reference to where data collection forms can be accessed, if not in the protocol

#### Examples

“The Psychotic Symptoms Rating Scales-Auditory Hallucinations subscale [PSYRATS-AH] is an interviewer-assessed measure tapping different dimensions of auditory hallucinations, e.g. frequency, duration and distress . . . The PSYRATS-AH (the primary outcome of the study) has been shown to have high inter-rater reliability (ranging from 0.79 to 1.00) as well as good validity [References]. Assessments will be conducted by trained psychologists at each treatment site. The main author of this article (LCS) is a psychologist with a specialisation in psychiatry. LCS is responsible for the training of all assessors. In the training process, supervised co-interviews are conducted. During the data collection phase, videotaped assessments are distributed among assessors for monthly consensus meetings. A random selection of video-taped interviews is scored individually by all assessors. ICC [Intraclass correlation coefficient] scores will subsequently be calculated and reported.”^379^

“All data are recorded in paper form. Data collection forms comprise a patient questionnaire, a GP [general practitioner] case report form, a patient diary, an optional dual-energy CT [computed tomography] case report form and a telephone interview questionnaire. All data collection forms are available from the corresponding author on request.

As this is a pragmatic study conducted in general practices, blood samples are analysed in the affiliated laboratories of the practices.

The dual energy examination takes place in the three university hospitals by trained staff. The procedure of the examination and the measurement parameters are defined in a standard operating procedure. To assess the quality of the reading, two trained radiologists will review a subset of the images independently. Inter- and intra-observer reliability will be determined.”^380^

#### Explanation

The validity and reliability of trial data depend on the quality of the data collection methods. The processes of acquiring and recording data often benefit from attention to training of study staff and use of standardised, pilot tested method, both of which should be identical for all study groups, unless precluded by the nature of the intervention.

The choice of methods for outcome assessment can affect study conduct and results. Substantially different responses can be obtained for certain outcomes (eg, harms) depending on who answers the questions (eg, the participant or investigator) and how the questions are presented (eg, discrete options or open ended).^381^ ^382^ ^383^ ^384^ Also, when compared with paper based data collection, the use of mobile devices and electronic data capture systems has the potential to improve protocol adherence, data accuracy, user acceptability, and timeliness of receiving data.^385^

The quality of data also depends on the reliability, validity, and responsiveness of data collection instruments, such as questionnaires and laboratory instruments.^386^ ^387^ Instruments with low interrater reliability will reduce statistical power, whereas those with low validity will not accurately measure the intended outcome variable. Modified versions of validated instruments might no longer be considered validated, and use of unpublished measurement scales can introduce bias and inflate treatment effect sizes.^388^ Routinely collected data, such as from administrative databases, are increasingly used for data collection in randomised trials, although they may be associated with reduced effect estimates.^389^

Standard processes are often implemented by local study staff to enhance data quality and reduce bias by detecting and reducing the amount of missing or incomplete data, inaccuracies, and excessive variability in measurements.^390^ Examples include standardised training and testing of outcome assessors to promote consistency, tests for validity or reliability of study instruments, and duplicate data measurements.

A review of UK government funded trials found that among 75 protocols with patient reported outcomes, only 37% described the instrument’s measurement properties.^391^ Among protocols from 2016, 32% to 54% described the staff who would be collecting data.^9^ ^10^

A clear protocol description of the data collection process, including the staff, methods, instruments, and measures to promote data quality, can facilitate implementation and help protocol reviewers to assess their appropriateness. If not included in the protocol, then a reference to where the data collection forms can be accessed should be provided. If performed, pilot testing and assessment of reliability and validity of the forms should also be described.

#### Summary of key elements to address

- Who will assess the outcome (eg, participant, doctor, nurse, caregiver)
- Who will collect the data (eg, participant, doctor, nurse, caregiver)
- Mode of data collection (eg, paper based data collection, mobile devices)
- Description of data collection instruments (eg, validated questionnaires, laboratory instruments), including reliability and validity
- Processes to promote quality of data collection (eg, duplicate measurements, training of assessors)
- Where the data collection form can be accessed (eg, appendix, link to repository)
- Any pilot testing and assessment of reliability and validity of the forms, if performed.

### Item 25b: Plans to promote participant retention and complete follow-up, including list of any outcome data to be collected for participants who discontinue or deviate from intervention protocols

#### Example

“Participant retention will be achieved by several strategies. Participant involvement throughout the trial development, activation and conduct, facilitated primarily by obtaining the Consumer Advisory Board’s input, ensures a patient-centred approach is applied to all trial activities and interactions with trial participants. The study staff at each site will be accessible to participants to answer their questions and respond to any concerns. To minimise the burden on a participant’s time, study visits will be scheduled to occur with any pre-existing clinic or other outpatient appointments in place for them when possible. Parking vouchers or travel reimbursements, to a maximum of $60 AUD, will be provided for visits that are study-specific and cannot be done remotely over the phone. Practical guidance and suggestions for participant retention awareness training will occur at the site initiation meetings and is documented in the Operations Manual.

For participants who withdraw from the trial, no further information will be collected from the date of withdrawal. If a participant at an intervention site does not receive the CGA [Comprehensive Geriatric Assessment intervention], or a participant at the control site receives a CGA, they will remain in the trial and be followed up until the end of the study and analyses will be conducted using the intention-to-treat principle.”^392^

#### Explanation

Trial investigators must often seek a balance between achieving a sufficiently long follow-up for clinically relevant outcome measurements^393^ and achieving a sufficiently short follow-up to decrease attrition and maximise completeness of data collection. Non-retention refers to instances where participants are prematurely off-study (ie, consent withdrawn or lost to follow-up) and thus outcome data cannot be obtained from them. Most trials will have some degree of non-retention, and the number of these off-study participants usually increases with the duration of follow-up.^394^

It is desirable to plan for how retention will be promoted to prevent missing data and avoid the associated complexities in both study analysis (item 27c) and interpretation. Participant retention can be improved,^205^ ^235^ ^395^ such as by financial reimbursement, systematic methods and reminders for contacting patients, scheduling appointments, and monitoring retention, as well as limiting participant burden related to follow-up visits and procedures (item 18).

Because missing data can be a major threat to trial validity and statistical power, data collection is often continued until a participant withdraws from the study.^396^ A participant who discontinues follow-up assessment for one outcome might be willing to continue with assessments for other outcomes, if given the option.

Non-retention should be distinguished from non-adherence.^397^ Non-adherence refers to deviation from intervention protocols (item 15c), but it does not mean that the participant is off-study and no longer in the trial. Non-adherence should not be an automatic reason for ceasing to collect data from the trial participant. It is widely recommended that all randomised participants should be included in the analysis in their assigned groups, regardless of adherence (item 27c).

Reviews of two samples of 108 and 292 trial protocols from 2016 found that only half reported strategies to promote participant retention and complete follow-up.^9^ ^10^

Protocols should describe any retention strategies and define which outcome data will be recorded from those who deviate from the planned intervention.^235^ Protocols should also detail any plans to record the reasons for non-adherence (eg, discontinuation of intervention owing to harms versus lack of efficacy) and non-retention (ie, withdrawn from trial or lost to follow-up), as this information can influence the handling of missing data and interpretation of results.^235^ ^398^

#### Summary of key elements to address

- Retention strategies to promote complete follow-up and prevent missing data
- List of outcome data that will be collected for participants who discontinue or deviate from intervention protocols
- Any plans to record the reasons for:
  - non-adherence (eg, discontinuation of intervention owing to harms versus lack of efficacy); and
  - non-retention (withdrawal from trial, loss to follow-up).

### Item 26: Plans for data entry, coding, security, and storage, including any related processes to promote data quality (eg, double data entry; range checks for data values). Reference to where details of data management procedures can be accessed, if not in the protocol

#### Example

“Household data, clinical measurements in cohort incidence study, and entomological data collected during the cross-sectional surveys will be captured on electronic forms using smartphones installed with ODK Collect. The data will be stored on a secure server located at LSHTM [London School of Hygiene and Tropical Medicine] and all data management and manipulation will be done using Stata (Stata Corp). Laboratory data output will be available directly from the analyser . . . and imported into a database. Data extractions will be converted into Stata format for querying and analysis. It will be possible to share de-identified data in several widely used formats.

Data quality and control

Paper case report forms (CRFs) will have numbered and coded items to ensure straightforward and accurate data entry and processing, and drafts will be reviewed by the study team before finalisation. Standard Operating Procedures (SOPs) for data collection will be developed and field staff will be appropriately trained to ensure rigorous data collection. This will include quality control (QC) of their own performance by checking for missing data or implausible responses. Furthermore, more QC checks will be performed by a supervisor to check for data completeness and internal consistency of responses within a few hours of data collection. Corrections, when appropriate, will be done before the CRFs are submitted for data entry. Electronic CRFs will have built in checks for missing data, implausible responses, and internal consistency; data collected using electronic CRFs will include the device serial number and date/time stamp and the device will be password protected. All quantitative data collected on paper CRFs will be double-entered into a database independently by two data clerks. The database will maintain an audit trail with time-date stamps of data entry and all changes that are made to the data . . .

Data security

Every effort will be made to ensure data security, particularly relating to sensitive participant information. All data will be uploaded onto a secure server on the LSHTM cloud. All data will be stored encrypted and will be accessible only by password and encryption keys held by the data manager. In the study database, we will not store any information that could be used to identify individual study participants. We will use anonymised study numbers as our unique participant identifier . . .

Data storage

Upon completion of the study, electronic files will be stored on a server and also copied to encrypted USB and stored offsite in a safebox. CRFs will be stored in the secure archive, which is equipped with locked cabinets for long term storage of CRFs and documents. All paper source records will be retained for a minimum of 10 years from the point of publication of data on the primary outcome. Electronic data will be stored for a minimum of 10 years following study completion, with regular checks to make sure that the data are still readable.”^399^

#### Explanation

Plans to handle the data collected from trial participants helps to promote data validity and integrity. A data management plan details how the data will be collected, processed, secured, stored, and shared both during and after a trial. Guidance is available on the content of data management plans.^400^ ^401^ ^402^

Differences in data entry methods can affect the trial in terms of data accuracy, cost, and efficiency. For example, when compared with paper case report forms, electronic data capture can reduce the time required for data entry and allow for efficient data validation, resolution of queries, and database release by combining data entry with data collection (item 25a).^385^ ^403^ When data are collected on paper forms, data entry can be performed locally or at a central site. Local data entry can enable fast correction of missing or inaccurate data, whereas central data entry facilitates blinding (masking), standardisation, and training of a core group of data entry staff.

Raw, non-numerical data are usually coded for ease of data storage, review, tabulation, and analysis. It is important to define standard coding practices to reduce errors and variation between observers. When data entry and coding are performed by different individuals, it is particularly important that the staff use unambiguous, standardised terminology and abbreviations to avoid misinterpretation.

Standard processes are often implemented to improve the accuracy of data entry and coding.^390^ ^404^ Common examples include double data entry,^405^ verification that the data are in the proper format (eg, integer) or within an expected range of values, and independent verification of the source document of a random subset of data to identify missing or apparently erroneous values. Although independent double data entry is performed to detect data entry errors, the time and costs need to be weighed against the magnitude of reduction in error rates compared with single data entry.

For trials in which both participants and staff are blinded, it is important to plan the timing and procedures for unblinding the trial, such as after the creation of a cleaned and locked data file.

Among two samples of trial protocols approved in 2016, 64% and 75%, respectively, reported the data entry and coding processes.^9^ ^10^ The protocol should fully describe the plans for data entry and processing, along with measures to promote their quality, or outline key elements with a reference to the data management plan where full information can be found. These details are particularly important for the primary outcome data. The protocol should also document data security measures to prevent unauthorised access to or loss of participant data, as well as plans for data storage (including time frame) during and after the trial. This information facilitates an assessment of adherence to applicable standards and regulations.

#### Summary of key elements to address

- Processes for data management, including:
  - data entry and coding, including measures to reduce errors (eg, double data entry, range checks for data values);
  - data security; and
  - data storage, including timeframe.
- Reference to where full information can be found (eg, data management plan), if not in the protocol.

### Item 27a: Statistical methods used to compare groups for primary and secondary outcomes, including harms

#### Examples

“Primary outcome analysis: The primary outcome (days alive and out of hospital within 90 days of randomisation) will be analysed using a mixed-effects negative binomial regression model, with a random-intercept for centre [reference]. The model will be adjusted for the minimisation factors of patient age and ASA [American Society of Anesthesiologists] class (I, II, III, IV, and V) [reference], as well as the following prognostic baseline covariates: urgency of surgery (immediate, urgent, and expedited), Glasgow Coma Score (GCS), systolic blood pressure, and pulse rate [reference]. Urgency of surgery and ASA class will be included as categorical variables, while patient age, GCS, systolic blood pressure, and pulse rate will be included as continuous variables. Patient age and GCS will be included assuming a linear association with the outcome, and systolic blood pressure and pulse rate will be included using restricted cubic splines with 3 knots (knots will be placed based on Harrell’s recommended percentiles) [references]. Missing baseline data will be handled using mean imputation for continuous variables, and a missing indicator variable for categorical variables [reference].

Secondary outcome analysis: Mortality within 90 days and 1 year of randomisation will be analysed using an analogueous mixed-effects logistic regression model (same random effects and covariate strategy as primary outcome). Duration of hospital stay and hospital re-admission will be analysed using a competing-risk time-to-event model, which includes mortality as a competing risk [reference]. Both models will adjust for the set of covariates specified above. Duration of stay in a level 2 or level 3 critical care bed will be analysed using a mixed-effects negative binomial regression model, with a random intercept for centre. The model will adjust for the set of covariates specified above.”^406^

“All primary comparisons between treatment arms will be on an intention-to-treat basis, that is, according to the group to which participants were randomised and without reference to their actual compliance with assigned treatment. Each of the co-primary endpoints will be analysed separately in time-to-event analyses. Event rates (time to first event within each endpoint definition) will be compared between groups using an HR and 95% CI from a Cox proportional hazards regression model fitted to the endpoint, with censoring for individuals not experiencing an endpoint event at their most recent study visit, and a single covariate being an indicator of the group to which the individual was randomised, statin or placebo. The proportional hazards assumption will be tested for each model. Loss to follow-up will be considered a censoring event. This equates to an assumption that data is missing at random given the participant’s treatment group and the timing of their loss to follow-up. The adequacy of this assumption will be checked in sensitivity analyses that will include both imputation approaches and adjustment for baseline covariates predictive of propensity for dropout.

A closed testing procedure will be used to allow for the multiple testing arising from two co-primary endpoints. This approach is based on the expectation that cardiovascular benefit will be the main contributor to improved disability-free survival and that a substantial effect of statins on the latter is unlikely in the absence of an effect on the former. First, major cardiovascular events will be tested at α=0.05 and, if the major cardiovascular events p value is <0.05 then second, disability-free survival will be tested at α=0.05. If the major cardiovascular events p value is not <0.05 than a p value for disability-free survival will not be presented.”^407^

#### Explanation

A clear and comprehensive account of the planned statistical methods for a trial facilitates implementation, replication, and critical assessment. Details of all statistical analyses are often reported in a full statistical analysis plan—a document that accompanies a trial protocol.^71^ Similar to a protocol, a statistical analysis plan should be date stamped and have any revisions documented (item 2).

The results for the primary and secondary outcomes can be substantially affected by the choice of methods used for analysis, which should align with the trial objectives, and, if used, the estimands framework (item 10). When more than one analysis method is applied to an outcome, there is potential for inappropriately selecting the approach that leads to the most “interesting” finding. Prespecifying the analysis plans in the protocol reduces the risk of selective reporting of outcomes and results.^63^ ^64^ ^81^ ^82^

Adjusting for baseline covariates, including those used in any stratified randomisation, is often advised in the analysis, particularly when a baseline covariate is prognostic of the outcome, as it can lead to improved power to detect an intervention effect.^408^ ^409^ ^410^

Trials are often affected by multiplicity issues.^411^ ^412^ When multiple comparisons are performed (eg, multiple outcomes, time points, subpopulations, interim analyses), the risk of false positives (type 1 error) is inflated. Although no standardised rules exist for dealing with multiplicity, guidance is available on key problems to consider.^411^ ^412^

Analysis plans for harms are challenging (item 17). Data will often consist of a mix of systematically and non-systematically assessed harms, making classic hypothesis testing difficult. Also, harms are often measured differently (eg, as events, rates, changes on a scale, or time to event), and, in general, the numbers involved are low. Randomised trials are thus often underpowered to detect typical harms of interest, such as a rare but clinically serious event that affects trial participants’ quality of life. Also, the relevant risk difference between the compared groups that is interesting to detect may be modest. However, the objective of analysing harms in randomised trials is not only to detect a statistically significant difference but also to identify preliminary evidence of possible harms (ie, a signal detection approach). It may be helpful to consider the analyses of harms in three scenarios: systematically assessed adverse events for hypothesis testing; signal detection in emerging common events; and descriptive analysis for less frequent events.^413^

Formal analytical approaches to harms data exist.^413^ If trial investigators decide to use descriptive approaches, this could involve reporting all adverse events in an appendix. Furthermore, planning a prospective meta-analysis can in some situations help achieve sufficient power by pooling the results from multiple trials.^414^

Among protocols for randomised trials approved in 2016, 87% detailed the main analysis plan for the primary outcome.^9^ ^10^ A systematic review found that analysis plans described in protocols for up to half of studies (mostly clinical trials and systematic reviews) did not match those reported in publications.^64^

The protocol should describe in sufficient detail the key considerations of the planned statistical analyses of the primary and secondary outcomes, regardless of whether there is a separate statistical analysis plan. It is important to specify the main analysis (often referred to as the primary analysis) of the primary outcome (item 16), including the analysis methods to be used for statistical comparisons, precisely which trial participants will be included (item 27b), and how missing data will be handled (item 27c). The protocol should also indicate the effect measure (eg, absolute risk) and the statistical significance level that will be used, as well as the intended use of confidence intervals when presenting the results. If applicable, any plans to perform an adjustment for multiple testing should be explained, including the rationale and the method of adjustment.

The protocol should also describe whether an adjusted analysis is to be performed, the covariates included in the adjusted analysis (or any criteria to select the covariates), and how any continuous covariates will be handled (eg, modelled assuming linearity or non-linearity).^415^ The potential for missing values in any of the covariates being adjusted for should be anticipated, and plans to handle any missing values should be described (item 27c). If both unadjusted and adjusted analyses are planned, then it is important to state which will be the primary analysis.

Some trials are designed based on bayesian methods.^416^ ^417^ In this case, the choices of priors, computational choices, and any modelling used should be described.

The same considerations apply to detailing the analysis of secondary outcomes in the protocol.

#### Summary of key elements to address

- Statistical methods for each analysis:
  - main analysis method for statistical comparison;
  - effect measure (eg, absolute risk) with confidence intervals;
  - statistical significance level; and
  - for bayesian analysis: choices of priors, computational choices, details of any modelling, and effect measure with credible intervals.
- For adjusted analyses (if applicable):
  - rationale for adjusted analyses;
  - list of covariates for adjustment;
  - statistical methods; and
  - if both adjusted and non-adjusted analyses are planned, which will be the main analysis.
- Methods to account for multiplicity, if applicable.
- Reference to the full statistical analysis plan, if a separate document exists.

### Item 27b: Definition of who will be included in each analysis (eg, all randomised participants), and in which group

#### Example

“Analysis populations are defined as follows:

- Intention to treat (ITT): this population includes all randomised participants regardless of whether they were later found to be ineligible, did not adhere to the protocol or were never treated.
- Per protocol (PP): this population contains all randomised participants who received their allocated trial treatment without major protocol deviations.
- Safety population: this population contains all randomised participants who received at least one dose of trial IMP and will be classified according to the actual treatment received.

The analysis of the primary outcome will be in the ITT population with sensitivity analysis in the PP population. The safety population will be used to report side effects.”^418^

#### Explanation

To preserve the benefits of randomisation, all randomised participants need to be included in the analysis and retained in the group to which they were allocated. An intention-to-treat analysis population is defined by the inclusion of outcome data from all participants in their originally allocated group—this is widely recommended as the preferred analysis population.^360^ ^419^ ^420^ Attrition bias can be avoided when outcome data are obtained from all participants and included in the analysis, regardless of adherence to the protocol. Although imputation of missing outcomes would allow an intention-to-treat analysis, it does not guarantee avoidance of bias except under strong assumptions about the missing data.

Many analyses are described as intention to treat but apply variable definitions in terms of handling missing outcome data (item 27c) or excluding participants who deviate from the intervention or follow-up protocols.^421^ ^422^ Some trials will define a per protocol analysis population that includes participants completing the study with no major protocol deviations. Exclusion of data from participants who did not adhere to the protocol can compromise the randomisation and introduce bias, particularly if the frequency of and the reasons for non-adherence vary between the trial groups (eg, healthy adherers bias).^423^ Depending on the aim of the analyses (box 1), other analysis populations may be planned, and their rationale and definition should be explained.

A meta-epidemiological study of 322 comparisons from 310 randomised trials found that analyses deviating from intention to treat produced larger intervention effect sizes than those applying the intention-to-treat principle.^424^ Other analysis populations may be planned (eg, a safety population), and their rationale and definition should be explained. Reviews of two samples of 108 and 292 trial protocols from 2016 reported that 69% and 74%, respectively, specified the analysis population.^9^ ^10^ Other reviews have found frequent discrepancies between the main analysis population described in protocols compared with in reports of completed trials (or systematic reviews).^64^

The protocol should clearly define the primary analysis population (and any other populations, as applicable). This includes considering whether all randomised participants (completely observed outcomes or imputed outcomes) or a subset of randomised participants with observed outcomes will be included in the analysis, and in which trial group. Ambiguous labels such as an intention-to-treat analysis or modified intention-to-treat analysis should be avoided unless they are fully defined in the protocol.

#### Summary of key elements to address

- Who will be included in the primary and other analyses (eg, all randomised participants with either observed or imputed outcome data):
  - any exclusions due to missing data or other reasons.
- Trial group in which participants will be analysed (eg, as randomised).

### Item 27c: How missing data will be handled in the analysis

#### Example

“We anticipate two types of missing data in this trial. The first are those that are due to mortality during the follow- up period, and the second is due to loss to follow-up. Our previous work in ICU [Critical Care Unit] survivors and other work in the surgical literature has shown that most post-ICU deaths happen within the first 30 days of discharge. We do not expect death rates to be different between randomization groups, and we will monitor death closely during the trial using both follow-up contact and information from the EMR [Electronic Medical Record]. The second type of missing data comes from participant withdrawal during follow-up or inability to contact. This may be more frequent in the usual care group than in the intervention group due to infrequent contact when compared to the intervention group. The mixed effects approach we propose is robust under the missing-at-random assumption (i.e., the probability of missing is unrelated to the missing outcomes). However, we will compare the baseline characteristics of patients with missing outcomes to those with complete outcome ascertainment to detect violation of this assumption. We will also perform sensitivity analyses using various methods of imputation or a full parametric likelihood approach assuming various patterns of missing data.”^425^

#### Explanation

Most randomised trials encounter some degree of missing outcome and covariate data.^422^ ^426^ Missing data negatively affect trials by reducing statistical power and introducing potential bias.^426^ Methods for handling missing data (including analysis of complete cases only) typically rely on unverifiable assumptions. It is thus important to develop and document strategies to maximise completeness of follow-up and prevent missing data from arising in the first place (item 25b).

Missing values can occur for various reasons, such as when participants withdraw consent for further data collection or fail to attend follow-up visits. Some reasons for missingness could be related to the treatment allocation, prognostic factors, or a particular adverse event or health outcome.^427^ The mechanism resulting in missing data affects the risk of bias and decisions on how best to handle the missing data.^428^ ^429^ The assumed missingness of data are usually described using three categories of convoluted terminology. The first category, missing completely at random, means that there is no systematic difference between missing and observed data—they have the same distributions. The second category, missing at random, means that missing data are systematically related to known aspects of the observed data (which enables statistical modelling). Both these categories are different from the third category, not missing at random.^430^

When planning to handle anticipated missing data, protocol authors have a choice between various methods, including imputing data, fitting a mixed effects model to repeated measures data, or omitting participants.^428^ ^429^ When the amount of missing outcome data are not large, all randomised participants with an outcome observed (a complete case population) can be planned to be included in the analysis population under a plausible mechanism for missing data (eg, missing at random). Sensitivity analyses can be planned to explore departures from this assumption, thereby using all randomised participants at least in sensitivity analyses (item 27d).^431^ Still, although imputation of missing outcomes allows an intention-to-treat analysis, it does not guarantee avoidance of bias except under strong assumptions about the missing data mechanism, which may be unknown. It is often recommended that participants with missing data be included in the analysis using multiple imputation, wherein the missing outcomes or covariates are estimated using other variables.^432^ Although imputation aligns with intention-to-treat analysis, it demands strong assumptions that may be challenging to justify or verify.

Simple imputation methods (eg, last observation carried forward) may seem appealing but are not advisable as they introduce bias and ignore the uncertainty induced by missing data, leading to confidence intervals that are too narrow.^433^

If randomised participants with missing data are omitted (a complete case analysis), then the analysis deviates from the intention-to-treat principle and can introduce bias (item 27b) depending on the amount of missing data and the mechanism resulting in the missing data. A complete case analysis also diminishes statistical power by reducing the sample size.

Despite the high prevalence and important effect of missing data, only 69% and 74% of two samples of trial protocols approved in 2016 considered statistical methods to handle missing data (and the analysis population relating to protocol non-adherence).^9^ ^10^

The protocol should outline how missing data will be handled in the analysis, including planned methods for imputing missing data and details of the variables that will be used in the imputation process, if applicable (box 6). It is also important to describe any planned sensitivity analyses that will explore the extent to which trial results vary under different assumptions about missing data (item 27d).

Box 6 Reporting recommendations to handle missing data*

1. Report any strategies planned to reduce missing data.
2. Report whether and how the sample size calculation will account for missing data, and the justification for these decisions (item 19).
3. For the main analysis of all outcomes, report the assumption about the missing data mechanism and the justification for this choice. For multiple imputation methods, report^435^:
  - What variables will be included in the imputation procedure
  - How non-normally distributed and binary or categorical variables will be dealt with
  - If statistical interactions will be included in the final analyses, whether they will also be included in imputation models
  - Whether imputation will be done separately by randomised group
  - The number of imputed datasets that are planned
  - How results from different imputed datasets will be combined
4. Report the method planned to handle missing data for the primary analysis (eg, complete case, multiple imputation) and the justification for the methods chosen, for all outcomes. Include whether or which auxiliary variables were collected and used.
5. Report methods planned to conduct any sensitivity analyses for missing data for all outcomes, and the justification for the assumptions and methods chosen
6. Report how data that will be truncated due to death or other causes will be handled, with a justification for the method (if relevant).

Adapted from Hussain et al.^434^

#### Summary of key elements to address

For each analysis:

- assumption about the missing data mechanism (eg, missing at random), with justification; and
- how missing data will be handled (eg, multiple imputation, model based approaches), with justification.

### Item 27d: Methods for any additional analyses (eg, subgroup and sensitivity analyses)

#### Examples

“The primary analyses for symptom resolution will be investigated to determine whether treatment effectiveness differs according to the following subgroups:

1. Presence of concomitant STI [sexually transmitted infection] (yes/no)
2. BV [bacterial vaginosis] confirmed by positive microscopy (yes/no)
3. Type of centre at which participant presented (sexual health clinic versus GP/other clinics)

Between-group treatment effects will be provided for each subgroup, but interpretation of any subgroup effects will be based on the treatment subgroup interaction and 95% CI, estimated by fitting an appropriate interaction term in the regression models. Since the trial is powered to detect overall differences between the groups rather than interactions of this kind, these subgroup analyses will be regarded as exploratory.”^436^

“Sensitivity analyses will be performed, based on the assumption that the missing outcomes are the worst or best possible in the different randomization groups. If these show that the conclusions may differ based on the missing values, further multiple imputation will be performed for the missing values. These analyses will consider the results of any losses at follow-up to the extent that they relate to differences in the measured variables (i.e., under the assumption of random missingness).”^437^

#### Explanation

Subgroup analyses are planned to look for evidence of whether the intervention effect varies between different subgroups (eg, younger and older participants, men and women). Sensitivity analyses are planned to examine the robustness of the primary analysis to different assumptions or analytical approaches.

Examining differences in subgroups involves assessing the statistical interaction between a variable and the intervention effect.^438^ Interactions can be presented by showing results on a figure (along with relevant information, such as point estimate and confidence interval for each subgroup, as well as the interaction P value).

Although commonly performed,^439^ ^440^ subgroup analyses are highly susceptible to bias and misinterpretation, particularly if they are inappropriately conducted or selectively reported. Post hoc subgroup analyses (ie, performed after looking at the data) should be avoided owing to the high risk of spurious (false positive) findings.^441^ For the same reason, it is problematic to conduct many subgroup analyses, even if prespecified. Defining subgroups based on variables measured after randomisation is susceptible to bias and should be avoided.^442^ Another common mistake is to conclude differences between subgroups on the basis of a statistically non-significant P value in one subgroup versus a statistically significant P value in another subgroup, or on the basis of whether confidence intervals overlap. Well documented subgroup differences tend to be far less common than claims made for such differences.^443^

Another consideration is the practice of categorising continuous variables to define subgroups. Although categorisation is often used for perceived simplicity and ease of communication, this approach has notable drawbacks. Cut-off points to define subgroups are often arbitrarily chosen without clinical or biological rationale. Categorisation also leads to the loss of information and a reduction of statistical power.

Among protocols published from 2006 to 2017 or approved in 2012 and 2016, 20% to 36% described subgroup analyses but often failed to report key details, including the rationale (unreported for 83% to 96% of protocols that described subgroup analyses), anticipated direction of effect (85% to 100%), specific variables (17% to 27%), tests for interaction (67% to 73%), and considerations of statistical power (88% to 97%).^439^ ^440^ Reviews have found that for more than half of trials the subgroup analyses reported in publications do not match those described in protocols,^64^ ^440^ raising concerns about post hoc analyses and selective reporting.

The rationale for examining any subgroups should be outlined in the study protocol, including which baseline variables will be explored and the hypothesised direction of the subgroup effect based on plausibility.^444^ If continuous baseline variables are to be categorised, then the rationale and the cut-off points should be stated. All statistical methods used to analyse any subgroups should be clearly described.

Sensitivity analyses have a different aim from subgroup analyses. Sensitivity analyses are planned to examine whether the (primary) trial results vary substantially under a range of different assumptions about the data, methods, and models.^445^ ^446^ Sensitivity analyses are often planned to explore the effect of missing data and any assumptions made in the primary analysis on the handling of missing data (item 27c). When the findings from a sensitivity analysis are consistent with the primary results, confidence that the primary results are valid can be increased.^445^ ^447^

The protocol should clearly describe all planned sensitivity analyses, their rationale, and the methods to be used.

#### Summary of key elements to address

For any planned subgroup analyses:

- baseline variables to be explored;
- rationale;
- statistical methods (eg, test of interaction); and
- cut-off points and rationale for categorisation of continuous baseline variables (if applicable).

For any planned sensitivity analyses:

- rationale; and
- statistical methods.

### Item 28a: Composition of data monitoring committee (DMC); summary of its role and reporting structure; statement of whether it is independent from the sponsor and funder; conflicts of interest and reference to where further details about its charter can be found, if not in the protocol. Alternatively, an explanation of why a DMC is not needed

#### Examples

“The DMC [Data Monitoring Committee] will be responsible for safeguarding the interests of the trial participants and for assessing the safety and efficacy of the interventions during the trial. Also, the DMC is responsible for monitoring the overall conduct of the trial. The DMC consists of three specialists with expertise in anaesthesiology, intensive care, and clinical research and thus holds clinical and statistical expertise as recommended [Reference]. The DMC is independent of the sponsor and other members of the research staff. The DMC will review de-identified data for safety at five predetermined milestones (200, 500, 1000, and 1500 enrolled patients), but can, at any time, require extra reviews. Unless group differences are observed that require unblinding (as determined by the DMC), the DMC will be blinded to treatment groups. The trial will continue while the DMC reviews data. After a review, the DMC will prepare a short report for the steering committee with recommendations for continuation, modifications, or termination of the trial. The final decision on potential modifications or termination will rest with the steering committee and the sponsor-investigator. A detailed charter for the DMC will be available on the STOP-COPD trial website after patient inclusion starts.”^448^

“The independent statistician of the IDMSC [Independent Data Monitoring and Safety Committee] will conduct one blinded interim analysis after 500 participants (50%) have been followed for 28 days. The alpha value for the interim analysis is 0.0054 as by the O'Brien-Fleming bounds, which preserves type I error at the usual 5%. [reference] The trial will be stopped early if the alpha cut-off is crossed at the interim analysis. The IDMSC will be provided with the following outcome data with the two groups masked (eg interventions coded as 0 and 1):

-Days alive without life support (ie invasive mechanical ventilation, circulatory support or renal replacement therapy (including days in between intermittent renal replacement therapy)) from randomisation to day 28.
-Number of participants with one or more SARs [serious adverse reaction] or SUSARs [suspected unexpected serious adverse reaction] from randomisation to day 28.”^449^

#### Explanation

Some trials plan a periodic inspection of the accumulating outcome data by trial group in case the data show clear benefit or harm and the trial needs to be discontinued. Data monitoring can also inform other aspects of the trial, such as recruitment, and identify the need for adjustments. In many cases, trials with a data monitoring committee will be those of long duration and with interventions with known risk of harms. Many trials do not, however, need a formal data monitoring committee^450^; typically trials with a short duration or known minimal risks.

A data monitoring committee will often comprise members from a variety of disciplines.^363^ ^451^ ^452^ The primary role of a data monitoring committee is to periodically review the accumulating data and recommend to the trial steering committee or sponsor whether the trial should continue, be modified, or be discontinued. Independence, particularly from the sponsor and trial investigators, is a key characteristic of the data monitoring committee and can be broadly defined as the committee comprising members who are “completely uninvolved in the running of the trial and who cannot be unfairly influenced (either directly or indirectly) by people, or institutions, involved in the trial.”^363^ ^452^

The use of a data monitoring committee was reported for 29% of trial protocols approved from 2000 to 2003 in Switzerland, Germany, and Canada,^453^ ^454^ and for about 40% of trials registered on ClinicalTrials.gov from 2007 to 2010. Reviews of two samples of 108 and 292 trial protocols from 2016 found that 58% reported whether a data monitoring committee would or would not be planned.^9^ ^10^

The protocol should state whether a data monitoring committee will or will not be planned, with rationale. If a data monitoring committee is planned, the protocol should name its chair and members. If the members are not yet known, the protocol can indicate the intended size and characteristics of the membership. The protocol should also indicate the data monitoring committee’s roles and responsibilities, planned method of functioning, and degree of independence from those conducting, sponsoring, or funding the trial.^54^ ^363^ ^452^ ^455^ A data monitoring committee charter is recommended for detailing this information and should be included or referenced in the protocol.^54^ ^452^

#### Summary of key elements to address

- Whether a data monitoring committee is planned, with rationale
- If a data monitoring committee is planned:
  - composition of the committee;
  - size and characteristics of membership (eg, type of expertise);
  - chair and member names (if known);
  - roles and responsibilities;
  - reporting structure;
  - method of operation (eg, meeting format and frequency);
  - degree of independence from those conducting, sponsoring, or funding the trial; and
  - reference to data monitoring committee charter where further details can be found.

### Item 28b: Explanation of any interim analyses and stopping guidelines, including who will have access to these interim results and make the final decision to terminate the trial

#### Example

“There will be one formal interim analysis to test the primary hypothesis. The decision boundaries at the interim analysis are calculated for either stopping for futility or stopping for efficacy using the O’Brien-Fleming error spending function [References]. Since we adopt the non-binding futility boundary [References], if it is decided the study will continue due to other considerations even if we cross the futility boundary at the interim look, there will be no inflation of type I error . . . In our design setting, even if the efficacy boundary is crossed at the interim look, the study may continue and there will be no inflation of type I error, as efficacy is already established at the interim. The interim analysis will occur when 528 events (50% information) are collected.

If the interim result meets the stopping criterion for futility, that is, the one-sided p-value for Wald’s test on the log(hazard ratio) between SR [spatial repellent] and placebo at the interim is >0.3450, the study may stop for futility . . . If the one-sided p-value <0.00882, then the study can stop for efficacy; otherwise, the study will proceed. However, if it is decided the study will continue due to other considerations even if we cross the efficacy boundary at the interim look, there will be no inflation of type I error, as efficacy is already established at the interim.

If the one-sided p-value at the interim is >0.00882 but <0.3450, then the study will continue. At the final analysis, if the one-sided p-value from Wald’s test on the log(HR) between SR and placebo <0.04668, we will reject the null hypothesis, claiming SR reduces the malaria hazard rate compared to placebo in Kenya at the significance level of 5% . . .

Interim analysis data will be available to the DSMB [data and safety monitoring board], Funder, Sponsor, and SCJ [SC Johnson] along with the study oversight contractor, fhiClinical, and any ad hoc experts deemed appropriate. The DSMB has the ability to recommend stopping the trial based on safety concerns, but do not have the responsibility of stopping the trial due to their assessment of efficacy or futility. The responsibility to stop the trial is held by the Sponsor.”^456^

#### Explanation

Interim analyses can be conducted to formally monitor the accumulating data in clinical trials. They are generally performed in trials that have a data monitoring committee, longer duration of recruitment, and potentially serious outcomes.

The results of these analyses, along with non-statistical criteria, can be part of a stopping guideline that helps inform whether the trial should be continued, modified, or halted earlier than intended for benefit, harm, or futility.^457^ Criteria for stopping for harm are often different from those for benefit and may or may not employ a formal statistical criterion.^458^ Stopping for futility occurs in instances where, if the study were to continue, it is unlikely that an important effect would be seen (ie, low chance of rejecting the null hypothesis). Multiple analyses of the accumulating data increase the risk of a false positive (type I error), and various statistical strategies have been developed to compensate for this inflated risk.^459^ Aside from informing stopping guidelines, prespecified interim analyses can be used for other trial adaptations such as sample size re-estimation, alteration of the proportion of participants allocated to each study group, and changes to eligibility criteria.^460^

Interim analyses were described in a third of 894 trial protocols approved from 2000 to 2003 in Switzerland, Germany, and Canada.^454^ Such analyses were also reported in 71% (106/150) of cancer trial protocols with time-to-event outcomes in Italy from 2000 to 2005^461^; among 86 protocols with plans for efficacy interim analyses, 100% reported the timing of the analyses, 90% specified the overall reason for stopping (eg, superiority, futility), and 94% detailed the statistical approach.^461^

A complete description of any interim analysis plan, even if it is only to be performed at the request of an oversight body (eg, the data monitoring board), should be provided in the protocol—including the statistical methods, who will perform the analyses, and when they will be conducted (timing and indications). If applicable, details should also be provided about the decision criteria (statistical or other) that will be adopted to judge the interim results as part of a guideline for early stopping or other adaptations.

In addition, it is important to state who will see the outcome data while the trial is ongoing, whether these individuals will remain blinded (masked) to study groups, and how the integrity of the trial implementation will be protected (eg, maintaining blinding) when any adaptations are made to the trial.

A third of protocols for industry initiated randomised trials that received ethics approval in Denmark in 1994-95 stated that the sponsor had access to accumulating trial data, which can introduce potential bias due to conflicts of interests.^38^ Finally, the protocol should specify who has the ultimate authority to stop or modify the trial, such as the principal investigator, trial steering committee, or sponsor.

#### Summary of key elements to address

Interim analyses:

- when they will be conducted (timing and indications), and by whom;
- statistical methods; and
- who will have access to interim results, and whether they will be blinded.

Stopping guidelines:

- any criteria (statistical or non-statistical) that will be used to inform decisions about early stopping or other adaptations (eg, sample size re-estimation); and
- who will make the decision to continue, stop, or modify the trial.

### Item 29: Frequency and procedures for monitoring trial conduct. If there is no monitoring, give explanation

#### Examples

“As part of a risk-based monitoring strategy, all centres will be visited on-site or virtually after enrolment of the first 10–15 patients. Key data will be verified for 100% of patients. Trial sites will then be assessed as being either with or without noticeable findings with respect to the trial protocol, data quality and good clinical practice (GCP). Monitoring will be performed by the study centre at the site of the coordinating investigator at LMU Munich. The monitor (affiliated to the sponsor of the trial) reviews the eCRF [electronic case report form] for completeness and accuracy and instructs site personnel to make any required corrections. During monitoring visits, the monitor will ensure that data documented in the eCRF are in line with underlying source data. Sites without noticeable problems will receive no further on-site monitoring visits. Centres assessed as having noticeable problems will be visited again within 4 months. If problems persist, visits will be repeated three times per year, and key data will be verified for at least 50% of patients at the respective site. If no more findings occur, monitoring visits will no longer be required.”^462^

“Central monitoring

A detailed monitoring plan will be developed. In summary, the CTU [Clinical Trials Unit] will closely monitor the trial to ensure the rights, safety, and wellbeing of the trial participants and to ensure the accuracy of the data. All coordinating centres and site trial teams will be trained in the trial procedures and provide extensive guidance. Central monitoring methods will be used by the CTU. A sample of consent forms from all sites will be monitored at the CTU to make sure they are properly completed. In addition, data management and statistical checks of data (central statistical monitoring) will be done to ensure that trial participants meet the inclusion criteria and trial treatment is administered in line with the protocol. Event rates for primary and secondary outcomes will be monitored. Sites with higher or lower than expected event rates will be selected for further monitoring. Quantitative variables (systolic blood pressure (SBP), heart rate (HR), respiratory rate and blood loss) will be monitored to check the accuracy of the data. For example, the coefficient of variation for the data at each site will be examined and those where there is any reason for concern will be selected for further monitoring.

Monitoring at local site

Onsite monitoring will be carried out at any site flagged as high risk on central statistical monitoring and other central monitoring procedures. Source data verification will be done on at least 10% of the trial data. Additionally, site self-monitoring will be carried out where needed. This will involve the PI [Principal investigator]/delegate at a site monitoring themselves against a standardised checklist. The LSHTM [London School of Hygiene and Tropical Medicine] CTU will require investigators and their institutions to provide access to source data and all trial-related documents for monitoring, audits, Ethics Committee review and regulatory inspection. All trial-related and source documents including medical records, original consent forms and original CRFs [case report forms] must be kept safely. Investigators must plan in advance of the trial start where the trial-related documents will be stored and how they will be accessed. All documents must be made available when required for monitoring/audit/inspection during the course of the trial and for up for 5 years after the end of the overall trial.”^463^

#### Explanation

Clinical trial monitoring involves periodic review of core study processes, data, and documents by individuals not otherwise involved in site activities.^464^ ^465^ Trial monitoring is distinct from routine day-to-day measures to promote data quality (items 25a and 26). The purposes of such monitoring are to support the wellbeing of participants, improve data quality, ensure the trial is conducted as planned, and stimulate corrective action to prevent problems.^466^ The processes reviewed can relate to participant enrolment, consent, eligibility, and allocation to study groups, adherence to trial interventions and procedures to protect participants, including reporting of harms (item 17), and completeness, accuracy, and timeliness of data collection.

Trial monitoring was addressed in 13% to 76% of protocols approved in 2016 or published in 2019-20.^9^ ^10^ ^11^ ^467^

Some types of monitoring have stronger evidence for their benefits and efficiency than others. For example, central statistical monitoring has been shown to improve multiple indicators of data quality.^468^ ^469^ New, decentralised monitoring options are also becoming more widely available.^468^

In multicentre trials, monitoring can be performed using a variety of approaches applied centrally across all sites as well as to each site individually.^470^ Trial data and processes can be reviewed remotely or by performing on-site visits. Rather than reviewing 100% of accumulating data and processes, it is widely recommended that trials adopt a risk based approach to monitoring that focuses on the trial data and processes that are critical to study integrity, data validity, and the wellbeing of participants.^46^ ^467^ Risk based monitoring can also focus on sites that have, for example, the highest enrolment rates, large numbers of withdrawals, or atypical (low or high) numbers of adverse events.

The approaches (eg, central, remote, on-site), scope of procedures, and anticipated frequency of monitoring should be outlined in the protocol, including a description of the staff involved. If procedures are further detailed elsewhere (eg, a monitoring plan), then the protocol should reference where the full details can be accessed. An explanation should be provided if no monitoring is planned.

#### Summary of key elements to address

- Approach for monitoring (eg, central, remote, on-site, risk based)
- Scope of monitoring activities (eg, type and amount of data at each site)
- Anticipated frequency of monitoring activities
- Who will be involved in monitoring
- Reference to where further details can be found (eg, a monitoring plan)
- If no monitoring is planned, this should be stated with reasons.

### Item 30: Plans for seeking research ethics committee/institutional review board approval

#### Example

“The protocol, informed consent forms, and all other study material have been approved by the Ottawa Health Science Network - Research Ethics Board (20180452-01H). Each participating hospital’s Research Ethics Board (REB) has also approved the trial protocol. Consent material is available in French and English . . . PRICE-2 will be conducted according to Good Clinical Practice and based on the principles of the second version of the Tri-Council Policy Statement.”^344^

#### Explanation

Before participants are enrolled, a universal requirement for the ethical conduct of clinical trials is the review and approval of the protocol by qualified individuals who are not associated with the research team and have no disqualifying conflicts of interest as reviewers.^46^ ^471^ The review is typically conducted by a formal research ethics board or committee (also called an institutional review board) in accordance with jurisdictional policy.

The protocol should document where approval has been obtained or outline plans to seek such approval at all sites.

#### Summary of key elements to address

- Plans to obtain research ethics committee or institutional review board approval.

### Item 31: Plans for communicating important protocol modifications to relevant parties

#### Example

“The Sponsor is authorized to amend the protocol. All important protocol modifications will be first discussed within the Steering committee and then communicated to the relevant parties (local investigators, EC [ethics committee], trial registry) by the Sponsor. Substantial amendments will only be implemented after approval by the EC, whereas non-substantial amendments are communicated by the Sponsor to the EC within the annual safety report . . . Amended protocols will be sent to the study sites for filling in the Investigator Site File, and training on new documents will be documented on site.”^472^

#### Explanation

The trial protocol that is initially approved by the research ethics committee undergoes subsequent modification in 34% to 59% of trials.^35^ ^216^ ^249^ ^473^ ^474^ Numerous studies have shown substantive changes between prespecified methods (eg, as stated in approved protocols, registries, or regulatory agency submissions) and those reported in trial publications, including changes to primary outcomes,^254^ ^256^ ^257^ ^475^ sample size calculations,^200^ eligibility criteria,^215^ ^216^ ^476^ and interventions,^477^ as well as methods of allocation concealment,^329^ blinding,^5^ and statistical analysis.^200^ ^478^ ^479^ These substantive modifications are rarely acknowledged in the final trial reports.^200^ ^249^ ^250^ ^480^

While some amendments may be unavoidable, reviews of pharmaceutical industry trials found that a third to a half of amendments could have been avoided with greater attention to key problems in the protocol.^35^ ^474^ Substantive amendments can create challenges to data analysis and interpretation if they occur part way through the trial (eg, changes in eligibility criteria),^481^ and they can introduce bias if the changes are made based on the trial data.^256^ ^257^ ^475^ The implementation and communication of amendments is also burdensome and potentially costly.^474^

It is important that substantive amendments to the protocol are reviewed by an independent party, such as the research ethics committee, and transparently described in trial reports. The notion of “substantive” is variably defined by authorities, but in general it refers to a protocol amendment that can affect the safety of trial participants or the scientific validity, scope, or ethical rigour of the trial.^482^ ^483^

In a sample of trial protocols approved in 2016, 66% to 77% reported how amendments would be handled.^9^ ^10^

The protocol should describe the process for making amendments, including who will be responsible for the decision to amend the protocol and how substantive changes will be communicated to relevant parties (eg, research ethics committee, trial registries, regulatory agencies). Early consideration of extenuating circumstances that might be encountered during a trial can help ensure that appropriate mitigating strategies are approved in advance.^73^ Version control using protocol identifiers and dates (item 2), as well as a list of amendments, can help to track the history of amendments and identify the most recent protocol version.

#### Summary of key elements to address

- Process for making protocol amendments, including:
  - decision making authority for protocol amendments; and
  - how substantive changes will be communicated to relevant parties (eg, research ethics committees or institutional review boards, trial registries, regulatory agencies).

### Item 32a: Who will obtain informed consent or assent from potential trial participants or authorised proxies, and how

#### Examples

“Each child has an audiometry assessment and a clinical assessment (both routine procedures for those attending these clinics) before they are assessed for eligibility to enter the trial. The participating clinician further assesses eligibility, provides the potential participant’s parent/legal guardian with a verbal description of the trial and, if they are interested, provides a comprehensive Participant Information Sheet (PIS). All potential participants’ parents/legal guardians are given sufficient time to read the PIS, ask questions and consider participation before being asked to provide written informed consent if they are willing for their child to participate. Age-appropriate pictorial information sheets are also be provided for children who are old enough to use them.

Parents/legal guardians who consent to take part are asked to sign a consent form, which is also signed by the clinician who is taking consent. Parents/legal guardians are informed that they have the right to withdraw consent from participation in the OSTRICH trial at any time, and that the clinical care of their child is not affected at any time by declining to participate or withdrawing from the trial. Assent may be given by children who are able to understand the age-appropriate information provided and express an opinion regarding their participation.”^484^

“An investigator team composed mainly of nurses has been trained in all study procedures . . . Written informed consent will be obtained from each participant before any trial-related procedures are carried out.”^485^

#### Explanation

The notion of acquiring informed consent involves the presentation of understandable and comprehensible information about the research to potential participants, confirmation that they understand the research, and assurance that their agreement to participate is voluntary. The process typically involves discussion between the potential participant and an individual knowledgeable about the research, the presentation of written material (eg, an information leaflet or consent document), and the opportunity for potential participants to ask questions. Assent represents a minor’s affirmative agreement to participate in the trial, which typically involves signing a document that provides information about the study that is age appropriate.

The content, quantity, and mode of delivery of consent information can affect trial recruitment; participant comprehension, anxiety, and retention rates; and costs.^207^ ^486^ ^487^ ^488^ ^489^

Reviews of two samples of 108 and 292 trial protocols from 2016 found that 15% to 18% of trials protocols approved in 2016 did not describe the process of obtaining informed consent.^9^ ^10^

The protocol should include details of the consent process as well as the role, experience, and training (if applicable) of the individuals obtaining consent or dissent. In paediatric trials, where applicable regulations might stipulate obtaining affirmative assent for participation from children above a certain age,^490^ the protocol should describe how pertinent information will be provided to potential participants and how their understanding and assent will be ascertained. When potential participants lack decisional capacity for reasons other than young age (eg, mental status), and proxy consent can be obtained from a legally authorised representative, the protocol should describe who will determine an individual’s decisional capacity, whether a formal capacity instrument will be utilised, and how the individual’s informed agreement to continue participation will be secured should they regain decisional capacity.^491^ For certain trials, such as cluster randomised trials, it may not be possible to acquire individual informed consent from participants before randomisation, and the consent process may be modified accordingly. In these instances, an explanation should be provided in the protocol.^492^

#### Summary of key elements to address

- Role, experience, and training of individuals obtaining consent.
- How consent will be obtained from potential participants.
- If applicable, how assent will be obtained from paediatric participants who are too young to consent, including:
  - how information will be provided to potential participants; and
  - how their understanding and assent will be ascertained.
- Any plans to obtain proxy consent from potential adult participants who lack decisional capacity, including:
  - who will determine the individual’s decisional capacity;
  - any formal capacity instrument to be used; and
  - any plan for securing informed agreement to continue participation once decisional capacity is regained.

### Item 32b: Additional consent provisions for collection and use of participant data and biological specimens in ancillary studies, if applicable

#### Example

“In order to maximise site participation and limit barriers to trial enrolment, the core protocol of ASCOT ADAPT does not involve any mandatory collection of biological samples. Nonetheless, there are many important research questions related both to specific therapeutic interventions and the cohort of participants enrolled in ASCOT ADAPT, and sites and participants can elect to contribute samples towards a COVID-19 biobank. Biological specimens collected include respiratory viral swabs, peripheral blood mononuclear cells, serum and plasma, collected at timepoints from baseline to 14 days after enrolment. A tiered consent from participants may allow samples or information to be used for any approved research projects and may also allow for host genomic studies to be undertaken with stored samples.

Biological specimens collected for ASCOT ADAPT are stored in participating biobank facilities, with a central ‘virtual biobank’ catalogue of samples and consent. Samples are collected, processed and stored according to central laboratory procedures. Access to samples for ethics-approved protocols is considered through a central biobank committee, where guiding principles for access are equity and maximising scientific value. Storage of specimens is provided by the ASCOT ADAPT study, with retrieval and transport costs funded by external researchers accessing specimens.”^493^

#### Explanation

Ancillary studies involve the collection or derivation of data and biological specimens for purposes that are separate from those of the main trial. The acquisition and storage of data and biological specimens for ancillary studies are increasingly common in the context of clinical trials. Data and specimens may be used for a specified subset of studies or for unspecified future research.

Ancillary studies have additional considerations relating to consent. Guidance for the creation of a simplified informed consent document for biobanking is available.^494^ ^495^ Participants can be given several options to consider for their participation in ancillary research: consent for the use of their data and specimens in existing studies, consent for the use of their data and specimens in future research unrelated or related to the clinical condition under study, consent for sharing of their data and specimens with other institutions, and consent to be contacted by trial investigators for future studies. This is commonly referred to as tiered consent. Participants should also be informed about whether their withdrawal from the ancillary research is possible (eg, the data and specimens are coded and identifiable), what withdrawal means in this context (eg, utilised specimens and data derived from them cannot be withdrawn), and what information derived from the specimen related research will be provided to them, if any.

If data or biological specimens collected from trial participants may be used for ancillary studies, the protocol should outline how the data and specimens will be collected and stored and how consent will be obtained for their use.

#### Summary of key elements to address

- How consent will be obtained for using participant data and biological specimens in specified or unspecified ancillary studies
- How the data and specimens will be collected and stored for ancillary studies.

### Item 33: How personal information about potential and enrolled participants will be collected, shared, and maintained in order to protect confidentiality before, during, and after the trial

#### Examples

“Participant confidentiality is strictly held in trust by the Site Principal Investigator, participating investigators, research staff, and the Murdoch Children’s Research Institute (MCRI) and their agents. This confidentiality is extended to cover testing of biological samples in addition to the clinical information relating to participating participants.

To preserve confidentiality and reduce the risk of identification during collection, analysis and storage of data and information, the following will be undertaken:

(1) The number of private/confidential variables collected for each individual has been minimised. The data collected will be limited to that required to address the primary and secondary objectives

(2) Participant data and samples will be identified through use of a unique participant study number assigned to the study participant (“re-identifiable”).

The Site Principal Investigator is responsible for the storage of a master-file of names and other identifiable data with the participant ID; access to this document will be restricted to members of the research team and authorised persons as listed previously. The master file will be stored securely, and separately, from study data in locked/ password-protected databases with passwords kept separately.

(3) Separation of the roles responsible for management of identifiers and those responsible for analysing content. The data will be analysed by members of the research team, who will be provided with anonymised data identified only by the unique participant study ID . . .”^496^

“To ensure confidentiality, any data dispersed to investigators will be blinded of any identifying participant information.”^85^

#### Explanation

Personal information about trial participants is acquired during the process of recruitment, eligibility screening, and data collection. Much of this information consists of private details of which many people wish to maintain control, such as their health status, personal genotype, and social and family history.

Researchers need to safeguard confidential participant data from potential data breaches,^497^ sometimes while simultaneously implementing appropriate procedures for data sharing (item 6).^498^ ^499^

Reviews of two samples of 108 and 292 trial protocols approved in 2016 found that 76% and 88%, respectively, considered confidentiality of data.^9^ ^10^

The protocol should describe the means whereby personal information is collected, kept secure, and maintained. In general, this involves the creation of coded, deidentified data where the participant’s identifying information is replaced by an unrelated sequence of characters; secure maintenance of the data and the linking code in separate locations using encrypted digital files within password protected folders and storage media; and limiting access to the minimum number of individuals necessary for quality control, audit, and analysis. The protocol should also describe how the confidentiality of data will be preserved when the data are transmitted to sponsors, co-investigators, and external parties (item 6).

#### Summary of key elements to address

- How confidentiality will be preserved when:
  - collecting and maintaining personal information before, during, and after the trial; and
  - transmitting data to sponsors, co-investigators, and external parties.

### Item 34: Provisions, if any, for ancillary and post-trial care, and for compensation to those who suffer harm from trial participation

#### Example

“Participants will receive ancillary care beyond the scope of the trial as required. Any adverse events or complications arising from trial participation will be promptly addressed, and necessary medical interventions will be provided at no cost to the participants. Ancillary care will extend to managing conditions unrelated to the trial that may arise during the study period, ensuring the overall well-being of the participants . . .

Post-trial care will be provided to participants, particularly for any ongoing effects or complications related to the trial interventions. Participants will have access to appropriate medical care and follow-up visits, ensuring continuity of care beyond the trial’s conclusion . . .

Provisions for compensation will be in place for participants who may suffer harm from trial participation. In the event of an adverse event directly attributable to the study interventions, compensation will cover medical expenses, additional treatments, and any other related costs. The compensation process will be transparent, fair, and in compliance with local regulations and ethical guidelines.”^500^

#### Explanation

The provision of ancillary care refers to care provided beyond that immediately required for the proper and safe conduct of the trial, and the treatment of immediate trial procedure related adverse events. It is generally agreed that trial sponsors and investigators have an ethical obligation to plan to provide care for participants’ healthcare needs that arise as a direct consequence of participation in a trial.^2^ ^501^ ^502^ It is also important to consider whether care should be provided for certain ancillary needs that may otherwise arise during trial participation. Provision of care for ancillary needs reflects the fact that participants implicitly, but unavoidably, entrust certain aspects of their health to the research team.

The scope of entrustment will vary depending on the nature of the trial (eg, setting, health condition under study, investigations performed). Additional factors that influence the strength of the responsibility to provide ancillary care include participants’ vulnerabilities, uncompensated burdens and harms, the intensity and duration of the participant-researcher relationship, and the degree to which participants are uniquely dependent on the research team for healthcare.

The Declaration of Helsinki states that “appropriate compensation and treatment for subjects who are harmed as a result of participating in research must be ensured . . . In clinical trials, the protocol must also describe appropriate arrangements for post-trial provisions.”^2^ This principle is particularly relevant when research enabling the development and regulatory approval of interventions is performed in countries where subsequent access to the interventions is limited by cost or lack of availability.^501^ ^502^ ^503^

Reviews of two samples of 108 and 292 trial protocols from 2016 found that 56% and 32%, respectively, addressed ancillary and post-trial care.^9^ ^10^

The protocol should describe any plans to provide or pay for ancillary care during the trial and identify any interventions, benefits, or other care that the trial organisers will continue to provide to participants and host communities after completion of the trial. Any plans to compensate participants for trial related harms should also be outlined. It should be clearly stated if no plans for ancillary and post-trial care exist, with a rationale for why not.

#### Summary of key elements to address

- Any plans to provide or pay for ancillary care during the trial
- Any care or benefits that will be provided to participants or host communities after trial completion
- Any plans to compensate participants for trial related harms
- If no plans for ancillary and post-trial care, this should be stated with reasons.

## Discussion

This explanation and elaboration document describes the rationale and scientific background for the 34 items included in the SPIRIT 2025 checklist, presents examples of good reporting in protocols, and provides references to key empirical and theoretical evidence. The document serves as a resource for trialists and others who are interested in the background for the selection and formulation of each of the checklist items and who seek more detailed guidance for reporting.

SPIRIT 2025 has several strengths: its systematic and transparent development methods, the participation of a wide range of interested parties, including patients and members of the public; use of empirical evidence to support its recommendations; availability of detailed guidance, including model examples from protocols; and its broad implementation strategy. Developing SPIRIT 2025 and CONSORT 2025 together was an opportunity to align both checklists and provide users with consistent guidance in the reporting of trial design, conduct, and analysis, from trial protocol to final publication. Streamlining and harmonising the reporting recommendations might improve usability and adherence, leading to more complete reporting across key trial documents.

A challenge with SPIRIT 2025, as with any reporting guideline, is to achieve a reasonable balance between brevity and comprehensiveness. We stress that the guideline is intended to specify the minimum issues to address in a protocol and we have striven to keep the checklist as short as possible. We developed an expanded checklist incorporating the core content of the explanation and elaboration paper in short format (see supplementary appendix), which may be helpful during the writing phase of a protocol. This SPIRIT 2025 explanation and elaboration paper, including its comprehensive list of references, serves to fulfil the needs of those seeking more detail and context.

Another challenge is the balance between generalisability and specificity. The ambition of SPIRIT is to serve as a general reporting guideline applicable to protocols for most randomised trials. We have focused on the most prevalent design, the parallel group randomised trial, although most items are also applicable to other designs (eg, crossover trials). Still, each randomised trial is different, depending on its setting, aim, methods, outcomes, and other features. This variation has prompted several extensions to SPIRIT, aimed at detailing reporting of factorial trials,^193^ n-of-1 trials,^504^ and early phase dose finding trials,^505^ as well as the reporting of different types of trial outcomes,^22^ ^23^ ^506^ pathology content,^507^ specific types of interventions,^21^ ^508^ and modifications in response to extenuating circumstances.^73^ The user friendliness of related protocol reporting guidelines would be improved were there less overlap and better coordination. We follow with interest an attempt to solve a similar challenge for the PRISMA (Preferred Reporting Items for Systematic Reviews and Meta-Analyses) guideline.^509^ We will engage with the leaders of SPIRIT extensions to implement a process for aligning them with the updated SPIRIT 2025 statement. In the meantime, we recommend that authors of trial protocols use the existing version of the relevant SPIRIT extensions.

Researchers planning a trial must report various components of a trial protocol to different audiences, such as local research ethics committees, multiple funders, and trial registries. The specific format and order of SPIRIT items is not essential for adherence to the guideline. Online tools for protocol authors, such as the SPIRIT Electronic Protocol Tool and Resource (SEPTRE), enable automated transfer of relevant information to trial registries (eg, ClinicalTrials.gov).^67^

Public access to trial protocols (in addition to trial publications) enables readers (eg, patients, peer reviewers, editors, healthcare providers, funders, researchers conducting systematic reviews, and guideline developers) to compare what was planned in a protocol with what was reported in a publication. Comparisons between protocols and trial reports have identified considerable problems with selective reporting of outcomes and trial results.^64^ ^249^ We strongly suggest that any trial protocol should be made publicly available,^75^ ^77^ ^78^ preferably before randomisation of the first participant. This could be achieved by posting the protocol on a trial registry or preprint server (eg, OSF, Open Science Framework).

The combined SPIRIT-CONSORT website provides more information about both statements, including additional resources and training materials aimed at patients and the public, researchers, research trainees, journal editors, and peer reviewers.^8^ We invite interested parties to assist in the evaluation of the SPIRIT 2025 statement and explanation and elaboration paper by using the documents and providing feedback to inform future revisions.

We hope this explanation and elaboration paper can facilitate uptake and implementation of the SPIRIT 2025 statement and help to improve the completeness and transparency of protocols for randomised trials.

## Web extra

Extra material supplied by authors

Web appendix 1: SPIRIT 2025 expanded checklist detailing items to address in randomized trial protocols

## Notes

Contributors: AH, A-WC, IB, SH, DM, and KFS were responsible for the concept. AH, A-WC, IB, GSC, SH, DM, KFS, and RT drafted the text addressing individual reporting items. All authors then critically revised the manuscript for important intellectual content and approved the final version. AH is the guarantor. The corresponding author attests that all listed authors meet authorship criteria and that no others meeting the criteria have been omitted.

Patient and public involvement: The SPIRIT 2025 checklist items and the explanations here were developed using input from an international Delphi survey and consensus meeting. The Delphi survey was advertised through established patient and public networks, and 17 respondents self-identified as a patient or public representative and completed the Delphi survey. In addition, three participants in the expert consensus meeting were patient or public representatives who were leaders in advancing patient and public involvement.

Dissemination to participants and related patient and public communities: SPIRIT 2025 will be disseminated through a new website (www.consort-spirit.org), which will include materials designed for patients and the public.

Provenance and peer review: Not commissioned; externally peer reviewed.
