# Supplementary material for: SPIRIT 2025 explanation and elaboration: updated guideline for protocols of randomised trials
Source: BMJ. 2025 Apr 28;389:e081660. doi: 10.1136/bmj-2024-081660 (PMC12128891; doi:10.1136/bmj-2024-081660)
Supplement: Supplementary file 1 — Web appendix 1: SPIRIT 2025 expanded checklist detailing items to address in randomized trial protocols [file hroa081660.ww1.pdf]

Appendix

SPIRIT 2025 expanded checklist detailing items to address in randomized trial protocols

Cite this as: Hróbjartsson A, Boutron I, Hopewell S, et al. SPIRIT 2025 explanation and elaboration: updated guideline for protocols of randomised trials. *BMJ* 2025;389:e081660

**SPIRIT 2025**			**Expanded items to report**
**Section / Topic**	**No**	**SPIRIT 2025 checklist item description**	
**Administrative information**			
Title and structured summary	1a	Title stating the trial design, population, and interventions, with identification as a protocol	• Descriptive title stating:<br>○ Trial design (e.g., parallel group randomised trial)<br>○ Conceptual framework (e.g., superiority, non-inferiority)<br>○ Trial phase (if applicable)<br>○ Population<br>○ Intervention/comparator<br>○ Objective or primary outcome<br>○ "Protocol"
	1b	Structured summary of trial design and methods, including items from the World Health Organization Trial Registration Data Set	• Relevant items from the WHO Trial Registration Data Set:<br>○ Primary Registry and Trial Identifying Number<br>○ Secondary Identifying Numbers<br>○ Source(s) of Monetary or Material Support<br>○ Primary Sponsor<br>○ Contact for Public Queries<br>○ Contact for Scientific Queries<br>○ Public Title<br>○ Scientific title<br>○ Countries of Recruitment<br>○ Health Condition(s) or Problem(s) Studied<br>○ Intervention(s)<br>○ Key Inclusion and Exclusion Criteria<br>○ Study Type<br>○ Date of First Enrolment (planned)<br>○ Sample Size<br>○ Primary outcome(s)

			○ Key Secondary outcome(s)<br>○ Ethics Review<br>○ Individual Trial Participant Data sharing statement
Protocol version	2	Version date and identifier	• Version date<br>• Version identifier (e.g., Version 2.0)<br>• List of changes made relative to the previous protocol version, with reasons
Roles and responsibilities	3a	Names, affiliations, and roles of protocol contributors	For each protocol contributor:<br>• Name<br>• Affiliation<br>• Description of contributions, including use of artificial intelligence technologies, if applicable
	3b	Name and contact information for the trial sponsor	For the trial sponsor (e.g. individual, company, institution, or organization):<br>• Name<br>• Contact information<br>• Regulatory agency identifying number (if applicable)
	3c	Role of trial sponsor and funders in design, conduct, analysis, and reporting of trial; including any authority over these activities	For the Sponsor and funders:<br>• Roles and responsibilities in trial design, conduct, data analysis and interpretation, manuscript writing, and results dissemination<br>• Who will make final decision regarding the above trial aspects<br>• Whether the sponsor or funder will have the right to review or comment on the trial manuscript<br>• Any mechanisms used to mitigate funder influence. If the funder will have no direct involvement in the trial, then this should be explicitly stated.
	3d	Composition, roles, and responsibilities of the coordinating site, steering committee, endpoint adjudication committee, data management team, and other individuals or groups overseeing the trial, if applicable	For each trial committee:<br>• Roles and responsibilities<br>• Relationship to trial sponsor and funders<br>• Outline of membership (e.g., clinician, biostatistician, patient)<br>• Names of Chairs and members, when known<br><br>Examples of committees include:<br>• Trial Steering Committee (executive decisions)<br>• Trial Management Group (day-to-day trial conduct)<br>• Data Monitoring Committee (review of accumulating data)<br>• Endpoint adjudication committee<br>• Data management team<br>• Other individuals or groups overseeing the trial

Open science			
Trial registration	4	Name of trial registry, identifying number (with URL), and date of registration. If not yet registered, name of intended registry	• Name of registry<br>• Trial registry identifying number<br>• URL to registry record<br>• Date of registration
Protocol and statistical analysis plan	5	Where the trial protocol and statistical analysis plan can be accessed	• Where the protocol will be accessible (e.g., publication, repository such as Open Science Framework, trial registry)<br>• Where the full statistical analysis plan will be accessible
Data sharing	6	Where and how the individual de-identified participant data (including data dictionary), statistical code, and any other materials will be accessible	• What data and materials will be shared; for example:<br>○ De-identified participant data, data dictionary, analytical code used to process the data<br>○ Materials associated with the intervention (e.g., handbook or video for non-pharmacological interventions)<br>• How the data and materials will be shared with trial investigators and external parties, including:<br>○ Application process to access the data (if applicable)<br>○ Data transfer process (e.g., via repository or direct transfer to user)<br>○ Any plans to obtain consent from participants<br>• If no sharing is planned, this should be clearly stated
Funding and conflicts of interest	7a	Sources of funding and other support (e.g., supply of drugs)	For each funding source:<br>• Name of funder<br>• Type of funding:<br>○ Direct monetary support<br>○ Indirect support (free trial drugs, equipment, or services such as statistical analysis or use of medical writers)
	7b	Financial and other conflicts of interest for principal investigators and steering committee members	• Conflicts of interests for principal trial investigators and members of key committees involved in the trial (e.g. steering and data monitoring committees), including any of the following support received:<br>○ Financial: Salary support or grants; ownership of stock or options; honoraria (e.g., for advice, authorship, or public speaking); paid consultancy or service on advisory boards; and holders of patents or patents pending<br>○ Non-financial: academic commitments; personal or professional relationships; and other affiliations with special interests or advocacy positions

			• Any procedures planned to reduce the potential influence of conflicts of interest on the trial's design, conduct, analysis, or reporting<br>• If no conflicts of interest, this should be clearly stated
Dissemination policy	8	Plans to communicate trial results to participants, healthcare professionals, the public, and other relevant groups (e.g., reporting in trial registry, plain language summary, publication)	• Plan to disseminate trial results to participants, healthcare professionals, the public, and other relevant groups (e.g., reporting results in trial registry, preprint, plain language summary, publication in open access journal)<br>• Process and timeframe for approving and submitting reports for dissemination<br>• Authorship guidelines
**Introduction**			
Background and rationale	9a	Scientific background and rationale, including summary of relevant studies (published and unpublished) examining benefits and harms for each intervention	• Importance of the research question<br>• Why a new trial is needed in the context of available evidence<br>○ Explanation of how the intervention might work<br>○ Pre-trial evidence of the benefits and harms of the intervention<br>○ Reference to systematic review(s) of relevant trials; if none available, a summary of relevant evidence based on a systematic search
	9b	Explanation for choice of comparator	• Why the particular comparator group was chosen<br>• Whether the comparator represents standard of care
Objectives	10	Specific objectives related to benefits and harms	• Trial objectives related to benefits and harms including:<br>○ Participants<br>○ Intervention<br>○ Comparator<br>○ Primary outcome(s)<br>○ Time point of main interest<br>• Description of trial estimand(s), as appropriate
**Methods: Patient and public involvement, trial design**			
Patient and public involvement	11	Details of, or plans for, patient or public involvement in the design, conduct, and reporting of the trial	• Planned methods of patients and public involvement at different trial stages (e.g., design, conduct, reporting)<br>• Who is involved (e.g., patients, carers, members of the public)<br>• If no patient or public involvement planned, this should be stated
Trial design	12	Description of trial design including type of trial (e.g., parallel group, crossover), allocation ratio, and framework (e.g., superiority, equivalence, non-inferiority, exploratory)	• Type of trial design (e.g., parallel group)<br>• Conceptual framework (e.g., superiority, non-inferiority, or equivalence)<br>• Unit of randomisation (e.g., individual participant)<br>• Allocation ratio (e.g., 1:1)

Methods: Participants, interventions, and outcomes			
Trial setting	13	Settings (e.g., community, hospital) and locations (e.g., countries, sites) where the trial will be conducted	• Setting of participant recruitment (e.g., primary or tertiary care; outpatient community or hospital clinic; inpatient unit)<br>• Location(s) where the trial will be carried out (e.g., country, city)<br>• Planned number of sites
Eligibility criteria	14a	Eligibility criteria for participants	• Specific inclusion and exclusion criteria defining the trial population to be randomised
	14b	If applicable, eligibility criteria for sites and for individuals who will deliver the interventions (e.g., surgeons, physiotherapists)	If applicable:<br>• Eligibility criteria for sites (e.g., site volume for surgical procedure)<br>• Eligibility criteria for individuals delivering the interventions (e.g., surgeons, physiotherapists), such as professional qualifications, years in practice, skills, or validation of specific training before trial initiation
Intervention and comparator	15a	Intervention and comparator with sufficient details to allow replication including how, when, and by whom they will be administered. If relevant, where additional materials describing the intervention and comparator (e.g., intervention manual) can be accessed	• Details of each intervention and comparator to allow replication, including:<br>○ Components of the intervention and comparator<br>○ How they will be administered<br>○ When and for how long they will be administered<br>○ Any procedure for tailoring the intervention to individual participants<br>○ Any physical or informational materials to be used as part of the intervention/comparator (e.g., instruction manual) and where the materials will be made accessible<br>• When comparator group is "usual care":<br>○ Description of usual care and any plans to track and measure it during the trial<br>○ Whether the intervention group will also receive usual care
	15b	Criteria for discontinuing or modifying allocated intervention/comparator for a trial participant (e.g., drug dose change in response to harms, participant request, or improving/worsening disease)	• Criteria to guide modifications to trial intervention/comparator (e.g., drug dose change in response to harms, participant request, or improving/worsening disease)<br>• Criteria to guide discontinuation of trial intervention/comparator
	15c	Strategies to improve adherence to intervention/comparator protocols, if applicable, and any procedures for monitoring	• Strategies for improving fidelity of care providers and adherence of participants to intervention/comparator protocols, if applicable<br>• When and how fidelity of care providers and adherence of participants to intervention/comparator protocols will be assessed, if applicable<br>• Where appropriate, prespecified definition for classifying participants as being treated as planned or not

		adherence (e.g., drug tablet return, sessions attended)	
	15d	Concomitant care that is permitted or prohibited during the trial	• Relevant concomitant care that is allowed (e.g., rescue interventions) or prohibited during the trial<br>• Any plans to record concomitant care received, including “usual care”
Outcomes	16	Primary and secondary outcomes, including the specific measurement variable (e.g., systolic blood pressure), analysis metric (e.g., change from baseline, final value, time to event), method of aggregation (e.g., median, proportion), and time point for each outcome	• Specification of which outcomes are primary and secondary<br>• Rationale for the choice of trial outcomes and whether they are part of a core outcome set<br>• For each outcome:<br>○ Specific variable to be measured (e.g., Beck Depression Inventory score, all-cause mortality), with definition where relevant<br>○ Analysis metric for each participant (e.g., change from baseline, end value, time-to-event)<br>○ Summary measure for each study group (e.g., mean, proportion with score > 2)<br>○ Time point of interest for analysis (e.g., 3 months)
Harms	17	How harms are defined and will be assessed (e.g., systematically, non-systematically)	For each systematically assessed harm (active/targeted surveillance):<br>• Definition and measurement (e.g., name of validated questionnaire)<br>• Where appropriate, the metrics, method of aggregation, and time point of interest for analysis<br>• Procedures for harms assessment, including:<br>Who will do the assessment, and whether they will be blinded to the allocated trial group<br>○ Assessment time points and overall time period for recording harms<br><br>For each non-systematically assessed harm (passive surveillance):<br>• How data will be collected<br>• Assessment time points and overall time period for recording harms<br>• Process for coding each adverse event and grading its severity, including:<br>○ Who will do the coding and severity grading, and whether they will be blinded to the allocated trial group<br>○ Which coding and severity grading systems will be used, if any<br><br>For grouping of harms by seriousness, severity, body system, discontinuation of intervention (due to harms), and causality:<br>• Definitions of grouping categories

			• Who will do the grouping, and whether they will be blinded to the allocated trial group<br><br>If relevant:<br>• Process of reporting important adverse events to applicable groups (e.g., sponsor, regulator, data monitoring committee)
Participant timeline	18	Time schedule of enrolment, interventions (including any run-ins and washouts), assessments, and visits for participants. A schematic diagram is highly recommended (see Figure).	• Schematic diagram outlining the schedule and time commitment for trial participants, including:<br>○ Timeline of trial visits starting from eligibility screening to trial close-out<br>○ Timeline of interventions including any run-in and washout periods<br>○ Procedures and assessments performed at each visit, referencing specific data collection forms, if relevant
Sample size	19	How sample size was determined, including all assumptions supporting the sample size calculation	For sample size calculations:<br>• Primary outcome (and any other outcome) on which the calculations are based<br>• Outcome values (e.g., proportion) assumed for each group, with rationale<br>• Target difference in outcome values between trial groups (including common standard deviation for continuous outcomes), with rationale<br>• Statistical significance level or α (type I) error<br>• Statistical power or β (type II) error<br>• Any upward adjustments (e.g., accounting for missing data or non-adherence)<br>• Target sample size per trial group<br>• Any software used
Recruitment	20	Strategies for achieving adequate participant enrolment to reach target sample size	• Planned strategies to promote adequate enrolment (e.g., advertisements, pre-screening of health records, reducing participant burden)<br>• Where participants will be recruited (e.g., primary care clinic, community), by whom (e.g., surgeon), and when (e.g., time period after diagnosis)
**Methods: Assignment of interventions**			
Randomization:			
Sequence generation	21a	Who will generate the random allocation sequence and the method used	• Who will generate the allocation sequence<br>• Method of sequence generation (e.g., computerized random number generator)<br>• Any software used
	21b	Type of randomization (simple or restricted) and details of any factors for stratification. To reduce predictability of a	• Type of randomization: simple versus restricted (e.g., blocked); fixed versus adaptive (e.g., minimization); and where relevant, the reasons for such choices<br>• If applicable, factors (e.g., trial site, sex, disease stage) to be used for stratification, including categories and relevant cut-off boundaries.

		random sequence, other details of any planned restriction (e.g., blocking) should be provided in a separate document that is unavailable to those who enroll participants or assign interventions	• For restricted randomization: aside from the above, all other details on restriction (including minimization) should be provided in a separate document in order to reduce predictability of the random sequence
Allocation concealment mechanism	22	Mechanism used to implement the random allocation sequence (e.g., central computer/telephone; sequentially numbered, opaque, sealed containers), describing any steps to conceal the sequence until interventions are assigned	• How the individuals enrolling participants will be kept unaware of the next trial group assignment in the random sequence (not to be confused with blinding)
Implementation	23	Whether the personnel who will enroll and those who will assign participants to the interventions will have access to the random allocation sequence	• Who will have access to the random allocation sequence<br>• Who will enroll participants<br>• Who will assign participants to interventions<br>• Whether the personnel enrolling and assigning participants will have no access to the random allocation sequence<br><br>When individuals involved in sequence generation and allocation concealment are the same individuals involved in the implementation of assignment:<br>• How and where the random allocation list will be securely stored<br>• Any mechanisms to prevent those enrolling and assigning participants from accessing the list
Blinding	24a	Who will be blinded after assignment to interventions (e.g., participants, care providers, outcome assessors, data analysts)	• Who will be blinded to treatment assignments:<br>○ Trial participants<br>○ Care providers (i.e., those administering the intervention)<br>○ Outcome assessors (i.e., those who determine if a participant experienced the outcome of interest). e.g., the participant (for patient-reported outcomes), care provider, or independent observer<br>○ Data analysts performing the statistical analysis
	24b	If blinded, how blinding will be achieved and description of the similarity of interventions	For blinded trials:<br>• Mechanism to establish blinding (e.g., identical placebo, double-dummy)

			• Any similarities or differences in characteristics (e.g. appearance, taste) of the interventions being compared<br>• Any procedures intended to maintain blinding and reduce risk of accidental unblinding<br>• Any procedures intended to evaluate blinding procedures (e.g., pre-trial testing of blinding procedures)
	24c	If blinded, circumstances under which unblinding is permissible, and procedure for revealing a participant's allocated intervention during the trial	For blinded trials:<br>• Circumstances under which unblinding is permissible during the trial (e.g., to reduce immediate risk for a participant)<br>• Procedure for revealing a participant's allocated intervention during the trial
**Methods: Data collection, management, and analysis**			
Data collection methods	25a	Plans for assessment and collection of trial data, including any related processes to promote data quality (e.g., duplicate measurements, training of assessors) and a description of trial instruments (e.g., questionnaires, laboratory tests) along with their reliability and validity, if known. Reference to where data collection forms can be accessed, if not in the protocol	• Who will assess the outcome (e.g., participant, doctor, nurse, caregiver)<br>• Who will collect the data (e.g., participant, doctor, nurse, caregiver)<br>• Mode of data collection (e.g., paper-based data collection, mobile devices)<br>• Description of data collection instruments (e.g. validated questionnaires, laboratory instruments), including reliability and validity<br>• Processes to promote quality of data collection (e.g., duplicate measurements, training of assessors)<br>• Where the data collection form can be accessed (e.g., appendix, link to repository)<br>• Any pilot testing and assessment of reliability and validity of the forms, if performed
	25b	Plans to promote participant retention and complete follow-up, including list of any outcome data to be collected for participants who discontinue or deviate from intervention protocols	• Retention strategies to promote complete follow-up and prevent missing data<br>• List of outcome data that will be collected for participants who discontinue or deviate from intervention protocols<br>• Any plans to record the reasons for:<br>○ Non-adherence (e.g., discontinuation of intervention due to harms versus lack of efficacy)<br>○ Non-retention (withdrawal from trial, lost to follow-up)
Data management	26	Plans for data entry, coding, security, and storage, including any related processes to promote data quality (e.g., double data entry; range checks	• Processes for data management, including:<br>○ Data entry and coding, including measures to reduce errors (e.g., double data entry; range checks for data values):<br>○ Data security<br>○ Data storage, including time frame

		for data values). Reference to where details of data management procedures can be accessed, if not in the protocol	• Reference to where full information can be found (e.g., Data Management Plan), if not in the protocol
Statistical methods	27a	Statistical methods used to compare groups for primary and secondary outcomes, including harms	• Statistical methods for each analysis<br>○ Main analysis method for statistical comparison<br>○ Effect measure (e.g., absolute risk) with confidence intervals<br>○ Statistical significance level<br>○ For Bayesian analysis: choices of priors, computational choices, details of any modelling, and effect measure with credible intervals<br>• For adjusted analyses (if applicable):<br>○ Rationale for adjusted analyses<br>○ List of covariates for adjustment<br>○ Statistical methods<br>○ If both adjusted and non-adjusted analyses are planned, which will be the main analysis<br>• Methods to account for multiplicity, if applicable<br>• Reference to the full statistical analysis plan, if a separate document exists
	27b	Definition of who will be included in each analysis (e.g., all randomized participants), and in which group	• Who will be included in the primary and other analyses (e.g. all randomized participants with either observed or imputed outcome data)<br>○ Any exclusions due to missing data or other reasons<br>• Trial group in which participants will be analyzed (e.g., as-randomized)
	27c	How missing data will be handled in the analysis	For each analysis:<br>• Assumption about the missing data mechanism (e.g., missing at random), with justification<br>• How missing data will be handled (e.g., multiple imputation, model-based approaches), with justification
	27d	Methods for any additional analyses (e.g., subgroup and sensitivity analyses)	For any planned subgroup analyses:<br>• Baseline variables to be explored<br>• Rationale<br>• Statistical methods (e.g., test of interaction)<br>• Cut-points and rationale for categorization of continuous baseline variables (if applicable)<br>For any planned sensitivity analyses:<br>• Rationale<br>• Statistical methods

Methods: Monitoring			
Data monitoring committee	28a	Composition of data monitoring committee (DMC); summary of its role and reporting structure; statement of whether it is independent from the sponsor and funder; conflicts of interest and reference to where further details about its charter can be found, if not in the protocol. Alternatively, an explanation of why a DMC is not needed	• Whether a DMC is planned, with rationale<br><br>If DMC is planned:<br>• Composition of DMC<br>○ Size and characteristics of membership (e.g., type of expertise)<br>○ Chair and member names (if known)<br>• Roles and responsibilities<br>• Reporting structure<br>• Method of operation (e.g., meeting format and frequency)<br>• Degree of independence from those conducting, sponsoring, or funding the trial<br>• Reference to DMC charter where further details can be found
	28b	Explanation of any interim analyses and stopping guidelines, including who will have access to these interim results and make the final decision to terminate the trial	Interim analyses:<br>• When they will be conducted (timing and indications), and by whom<br>• Statistical methods<br>• Who will have access to interim results, and whether they will be blinded<br><br>Stopping guidelines:<br>• Any criteria (statistical or non-statistical) that will be used to inform decisions about early stopping or other adaptations (e.g., sample size re-estimation)<br>• Who will make the decision to continue, stop, or modify the trial
Trial monitoring	29	Frequency and procedures for monitoring trial conduct. If there is no monitoring, give explanation	• Approach for monitoring (e.g., central, remote, on-site, risk-based)<br>• Scope of monitoring activities (e.g., type and amount of data at each site)<br>• Anticipated frequency of monitoring activities<br>• Who will be involved in monitoring<br>• Reference where further details can be found (e.g., monitoring plan)<br>• If no monitoring is planned, this should be stated with reasons
**Ethics**			
Research ethics approval	30	Plans for seeking research ethics committee/institutional review board approval	• Plans to obtain research ethics committee/institutional review board approval
Protocol amendments	31	Plans for communicating important protocol modifications to relevant parties	• Process for making protocol amendments, including:<br>○ Decision-making authority for protocol amendments<br>○ How substantive changes will be communicated to relevant parties (e.g., REC/IRBs, trial registries, regulatory agencies)

Consent or assent	32a	Who will obtain informed consent or assent from potential trial participants or authorized proxies, and how	• Role, experience, and training of individuals obtaining consent<br>• How consent will be obtained from potential participants<br>• If applicable, how assent will be obtained from paediatric participants who are too young to consent, including:<br>○ How information will be provided to potential participants<br>○ How their understanding and assent will be ascertained<br>• Any plans to obtain proxy consent from potential adult participants who lack decisional capacity, including:<br>○ Who will determine the individual's decisional capacity<br>○ Any formal capacity instrument to be used<br>○ Any plan for securing informed agreement to continue participation once decisional capacity is regained
	32b	Additional consent provisions for collection and use of participant data and biological specimens in ancillary studies, if applicable	• How consent will be obtained for using participant data and biologic specimens in specified or unspecified ancillary studies<br>• How the data and specimens will be collected and stored for ancillary studies
Confidentiality	33	How personal information about potential and enrolled participants will be collected, shared, and maintained in order to protect confidentiality before, during, and after the trial	• How confidentiality will be preserved when:<br>○ Collecting and maintaining personal information before, during, and after the trial<br>○ Transmitting data to sponsors, co-investigators, and external parties
Ancillary and post-trial care	34	Provisions, if any, for ancillary and post-trial care, and for compensation to those who suffer harm from trial participation	• Any plans to provide or pay for ancillary care during the trial<br>• Any care or benefits that will be provided to participants or host communities after trial completion<br>• Any plans to compensate participants for trial-related harms<br>• If no plans for ancillary and post-trial care, this should be stated with reasons

SPIRIT–CONSORT website

www.consort-spirit.org
